# Discovery of anaerobic lithoheterotrophic haloarchaea, ubiquitous in hypersaline habitats

**DOI:** 10.1038/ismej.2016.203

**Published:** 2017-01-20

**Authors:** Dimitry Y Sorokin, Enzo Messina, Francesco Smedile, Pawel Roman, Jaap S Sinninghe Damsté, Sergio Ciordia, Maria Carmen Mena, Manuel Ferrer, Peter N Golyshin, Ilya V Kublanov, Nazar I Samarov, Stepan V Toshchakov, Violetta La Cono, Michail M Yakimov

**Affiliations:** 1 Winogradsky Institute of Microbiology, Research Centre of Biotechnology, Russian Academy of Sciences, Moscow, Russia; 2 Department of Biotechnology, Delft University of Technology, Delft, The Netherlands; 3 Institute for Coastal Marine Environment, CNR, Messina, Italy; 4 Sub-department of Environmental Technology, Wageningen University, Wageningen, The Netherlands; 5 Wetsus, Centre of Excellence for Sustainable Water Technology, Leeuwarden, The Netherlands; 6 Department of Marine Organic Biogeochemistry, NIOZ Royal Netherlands Institute for Sea Research, Den Burg, The Netherlands; 7 Proteomics Unit, National Center for Biotechnology, CSIC, Madrid, Spain; 8 Institute of Catalysis, CSIC, Madrid, Spain; 9 School of Biological Sciences, Bangor University, Bangor, UK; 10 Immanuel Kant Baltic Federal University, Kaliningrad, Russia

## Abstract

Hypersaline anoxic habitats harbour numerous novel uncultured archaea whose metabolic and ecological roles remain to be elucidated. Until recently, it was believed that energy generation via dissimilatory reduction of sulfur compounds is not functional at salt saturation conditions. Recent discovery of the strictly anaerobic acetotrophic *Halanaeroarchaeum* compels to change both this assumption and the traditional view on haloarchaea as aerobic heterotrophs. Here we report on isolation and characterization of a novel group of strictly anaerobic lithoheterotrophic haloarchaea, which we propose to classify as a new genus *Halodesulfurarchaeum*. Members of this previously unknown physiological group are capable of utilising formate or hydrogen as electron donors and elemental sulfur, thiosulfate or dimethylsulfoxide as electron acceptors. Using genome-wide proteomic analysis we have detected the full set of enzymes required for anaerobic respiration and analysed their substrate-specific expression. Such advanced metabolic plasticity and type of respiration, never seen before in haloarchaea, empower the wide distribution of *Halodesulfurarchaeum* in hypersaline inland lakes, solar salterns, lagoons and deep submarine anoxic brines. The discovery of this novel functional group of sulfur-respiring haloarchaea strengthens the evidence of their possible role in biogeochemical sulfur cycling linked to the terminal anaerobic carbon mineralisation in so far overlooked hypersaline anoxic habitats.

## Introduction

Extremely halophilic archaea of the class *Halobacteria* represent a unique branch of *Euryarchaeota* thriving in salt-saturating brines (Andrei *et al.*, 2012) thanks to an energetically favourable ‘salt-in’ osmoprotection strategy (Becker *et al.*, 2014). The emergence of the dominant aerobic heterotrophic haloarchaeal lifestyle is likely the result of a large influx of genes from aerobic bacterium to the common halophile ancestor, which transformed an ancient methanogen into an oxygen-respiring heterotroph (Rhodes *et al.*, 2011; Nelson-Sathi *et al.*, 2012, 2015; Wolf and Koonin, 2013; Sousa *et al.*, 2016). Corroborating with this hypothesis, most of the cultivated haloarchaea are aerobic heterotrophs with the exception of few examples of facultative anaerobes (Oren and Trüper, 1990; Oren, 1991; Antunes *et al.*, 2008; Bonete *et al.*, 2008; Andrei *et al.*, 2012; Werner *et al.*, 2014). At the same time, the molecular ecology studies based on SSU rRNA phylogeny demonstrated that highly reduced hypersaline environments are inhabited by a variety of unknown haloarchaea with no cultured representatives (Walsh *et al.*, 2005; Youssef *et al.*, 2011; Lamarche-Gagnon *et al.*, 2015), which could be involved in anaerobic sulfur and carbon cycling, as it was proposed in the past (Grant and Ross, 1986; Tindall and Trüper, 1986; Elshahed *et al.*, 2004a, 2004b). However, until recently, no conclusive evidence for that has been found, thus leaving unknown their metabolic capabilities and hence ecological roles. This has changed with the latest discovery of a strictly anaerobic acetate-oxidizing and S^0^-reducing haloarchaeon *Halanaeroarchaeum sulfurireducens* (HAA; Sorokin *et al.*, 2016a). The in-depth characterisation of cultivated representatives demonstrated that aerobic respiration is not any longer a universal feature in the haloarchaea (Messina *et al.*, 2016; Sorokin *et al.*, 2016a,b). Moreover, this previously overlooked metabolic type underscores the ongoing metabolic diversification within haloarchaea (Sousa *et al.*, 2016) and strengthens the evidence for involvement of this euryarchaeal branch in biogeochemical sulfur cycling linked to terminal anaerobic carbon mineralisation in hypersaline anoxic habitats. Further research into this direction yielded another ecotype of obligate anaerobic haloarchaea, which can be considered as lithoheterotrophic. Organisms grew with formate or hydrogen as the electron donors and sulfur compounds (elemental sulfur, thiosulfate and dimethylsulfoxide [DMSO]) as the electron acceptors, while yeast extract served as the carbon source. We propose to classify this novel group as a new genus and species *Halodesulfurarchaeum formicicum* (HDA). Noteworthy, this novel ecotype of haloarchaea was found in the same hypersaline ecosystems, where HAA was detected, suggesting the apparent functional niche diversification and eventual sympatric speciation. In the present study, we performed in-depth physiological and genomic characterisation of two HDA strains and assessed their functional respiratory properties through genome-wide proteomic studies of cultures grown on different electron acceptors and donors. A focus was put on the elucidation of features in HDA that promote its metabolic versatility.

## Materials and methods

### Sampling and establishment of enrichment cultures

Anoxic sediments were obtained from the hypersaline lakes of Kulunda Steppe, lakes Elton and Baskunchak and solar salterns of Eupatoria (Russia) and Bari (Italy). Additionally, anoxic brine was collected in the deep-sea hypersaline lake Medee from approx. 3,100 m depth (Yakimov *et al.*, 2013). Enrichment cultures were initiated by inoculating 1–10 ml of collected material into 90 ml of the mineral medium after Sorokin *et al.* (2016a). Elemental sulfur was added directly into each flask as a wet paste sterilised at 110 °C for 30 min at final concentrations of~50 mM. 2 M sodium thiosulfate (Sigma) and 1M DMSO stock solutions were filter-sterilized and added at 20 and 10 mM final concentrations, respectively. Other tested electron donors/acceptors were added with a syringe from sterile anaerobic 1M stock solutions at final concentrations 5–10 mM. Formate was supplied at the final concentration 30 mM. Routine cultivation was performed at 37 °C in 120 ml serum bottles with butyl rubber stoppers filled with the medium to 90% in case of formate and 50% in case of H_2_. Hydrogen was added through sterile gas filters at 0.5 bar overpressure on the top of argon atmosphere. The cultures were incubated at 37 °C with periodic shaking of the flasks. Growth in enrichments was monitored by measuring of HS^−^ formation. Since growth in the solid medium was not achieved, pure cultures were obtained by serial dilutions of subcultures to the extinction (up to 10^−10^) in 4–6 consecutive series and the final purity was verified microscopically and by 16S rRNA gene sequencing. Phase contrast microphotographs were obtained with a Zeiss Axioplan Imaging 2 microscope (Jena, Germany). For electron microscopy (JEOL-100, Japan), the cells were fixed with paraformaldehyde (3% w/v final) and stained with 1% (w/v) uranyl acetate.

### Chemical analyses

Sulfide formation was measured by using standard methylene blue method (Trüper and Schlegel, 1964) after fixing 10 μl culture supernatant in 0.5 ml 10% Zn acetate. Thiosulfate and sulfite were determined by iodimetry after removal of sulfide as ZnS. Sulfite was blocked by formaldehyde (3% final). Formate consumption was analyzed by HPLC (BioRad HPX-87H column at 60 °C; eluent 1.5 mM H_3_PO_4_, 0.6 ml min^−1^; UV/RI detector Waters 2489) after cell removal and fivefold diluting of samples with distilled water. The cell protein was determined by the Lowry method in 1–4 ml culture samples after centrifugation 13,000 rpm for 20 min. The cell pellets were washed with 4M NaCl solution at pH 5 to remove the cell-bound FeS. Polysulfides were analyzed after methylation, in the form of dimethyl polysulfides as described previously (Roman *et al.*, 2014). Volatile sulfur compounds in the gas phase were analysed by GC (Thermo Scientific TM Trace GC Ultra with Trace GC Ultra valve oven, Interscience, Breda, the Netherlands) equipped with FPD (150 °C), Restek column (RT-U-Bond, 30 m × 0.53 mM di × 20 μM df) as described previously (Roman *et al.*, 2015). Core membrane lipids and polar phospholipids were analyzed according to Weijers *et al.* (2009) and Sinninghe Damsté *et al.* (2011), respectively (see Supplementary Methods for details). Cytochrome oxidase activity was measured spectrophotometrically in sonicated cells in 4M NaCl buffered with 0.05 M K-P buffer using 1 mM reduced TMPD (tetramethyl-*p*-phenylendiamine hydrochloride) as substrate. Cytochrome spectra were recorded on the UV-Visible diode-array HP 8453 spectrophotometer (Hewlett Packard, Amsterdam, The Netherlands) with sodium ascorbate and sodium dithionite as reductants.

### Sequencing, assembly and annotation of genomes of strains HSR6 and HTSR1

We succeeded with isolation of 8 different strains, and genomes of the strains HTSR1 and HSR6 were sequenced. The HTSR1 genome was sequenced with MiSeq Personal Sequencing System technology of Illumina Inc. (San Diego, CA, USA) using paired-end 250-bp reads. For sequencing of HSR6 genome, both paired-end and mate-paired DNA libraries were used. Detailed descriptions of all methodological procedures used in this study can be found in the Supplementary Methods. Obtained reads were assembled with both ALLPATHS-LG (Butler *et al.*, 2008) and SPADES 3.7.0 (Nurk *et al.*, 2013) assemblers and refined by Geneious 7.1 software (Biomatters Ltd, New Zealand), resulting in fully closed circular chromosomes. Genes were predicted by Glimmer 3.02 (Delcher *et al.*, 2007), rRNA genes was predicted by RNAmmer 1.2 Server online tool (Lagesen *et al.*, 2007), while tRNA-coding sequences were predicted by tRNAscan-SE 1.21 online tool (Lowe and Eddy, 1997). Operon prediction was performed by using the FgenesB online tool (http://linux1.softberry.com/berry.phtml?topic=fgenesb&group=programs&subgroup=gfindb). For each predicted gene, the similarity search was performed by Geneious 7.1 BLAST embedded tool against public amino acid sequence databases (nr) and conserved domains families databases (COG, KEGG). Finally, annotations were manually curated using the Artemis 16.0 program (Rutherford *et al.*, 2001), and refined for each gene with NCBI blastx against nr and KEGG database (Altschul *et al.*, 1997; http://www.genome.jp/tools/blast/). The Average Nucleotide Identity (ANI) index was used to estimate the average nucleotide identity between HTSR1 and HSR6 (Goris *et al.*, 2007). 16S rRNA gene phylogeny of the HDA strains was inferred from a 16S rRNA gene sequence alignment with PAUP*4.b10 using a LogDet/paralinear distance method as it described elsewhere (Sorokin *et al.*, 2016a).

### Proteomic analyses

Shotgun proteomic analyses were conducted using the HTSR1 cells grown on formate/S^0^, formate/thiosulfate and formate/DMSO couples. Detailed descriptions of all methodological procedures used in this study (protein extraction, protein concentration, in-gel trypsin digestion and nano-liquid chromatography tandem mass spectrometry) can be found in the Supplementary Methods. Abundance of each detected protein was treated separately with a custom C++ Linux-Shell program in order to be accepted as User graph from the DNAPlotter tool inside Artemis 16.0 program (Rutherford *et al.*, 2001). Data normalisation was performed automatically by the program.

### Data and strains deposition

16S rRNA gene sequences were deposited in the GenBank database (accession no. from KX664089 to KX664094). The genome sequences of strains HSR6^T^ and HTSR1 have been submitted to the GenBank with accession numbers CP016804 and CP016070. All HSR formate-oxidizing isolates have been deposited in the UNIQEM culture collection (Collection of Unique Extremophilic Microorganisms, Russian Academy of Sciences, Moscow, Russia). The type strain HSR6^T^ (UNIQEM U983^T^) was additionally deposited in Japan Collection of Microorganisms under the number JCM 30662^T^.

## Results and discussion

### Enrichment and isolation of lithoheterotrophic sulfur-respiring haloarchaea

The eight novel sulfidogenic haloarchaeal isolates described in this study were obtained from anoxic sediment/brine samples taken from hypersaline circumneutral habitats at different geographical locations (Table 1). Most of the strains were enriched with a combination of formate as electron donor and elemental sulfur as electron acceptor, with a supplementation of yeast extract (10 mg l^−1^). Sulfide formation was registered after 2–4 weeks of incubation at 37 °C with maximal accumulation up to 5 mM within 2 months in samples from Kulunda Steppe. Addition of a mixture of streptomycin, ampicillin and vancomycin (100 mg l^−1^ each) did not affect the sulfidogenesis but shortened the isolation procedure. The active sediment slurry incubations were further used as an inoculum (1% vol/vol) in artificial medium containing 4M NaCl. However, the cell growth was extremely weak and after the third transfer the growth of cultures ceased. Increasing the concentration of yeast extract to 100 mg l^−1^ allowed the full recovery of the cultures. Apparently, these haloarchaea were heterotrophic and required yeast extract as the carbon source. Consecutive dilutions to extinction series produced six pure cultures of formate-dependent sulfur reducers designated as HSR6, HSR8, HSR9, HSR15, Bari-SA6 and Medee-SA6. Using thiosulfate as an electron acceptor instead of elemental sulfur resulted in isolation from the Kulunda Steppe salt lake sediments of an additional formate-oxidising sulfidogenic strain HTSR1. A combination of hydrogen as an electron donor and elemental sulfur as an acceptor resulted in isolation of a strain HSR14 from the Kulunda Steppe salt lake sediments. The characteristic property of this hydrogenotrophic isolate was a higher sulfide production at slower growth and lower biomass yield in comparison with the formate-oxidising cultures (Table 1 and Supplementary Figure S1). The tolerance to oxygen was checked by exposing the HDA cultures (10% [vol / vol] liquid to gas ratio) to the gas phase containing from 0.1 to 5% O_2_. No growth was observed in any trials. The tests for cytochrome oxidase were also negative.

**Figure 1:**
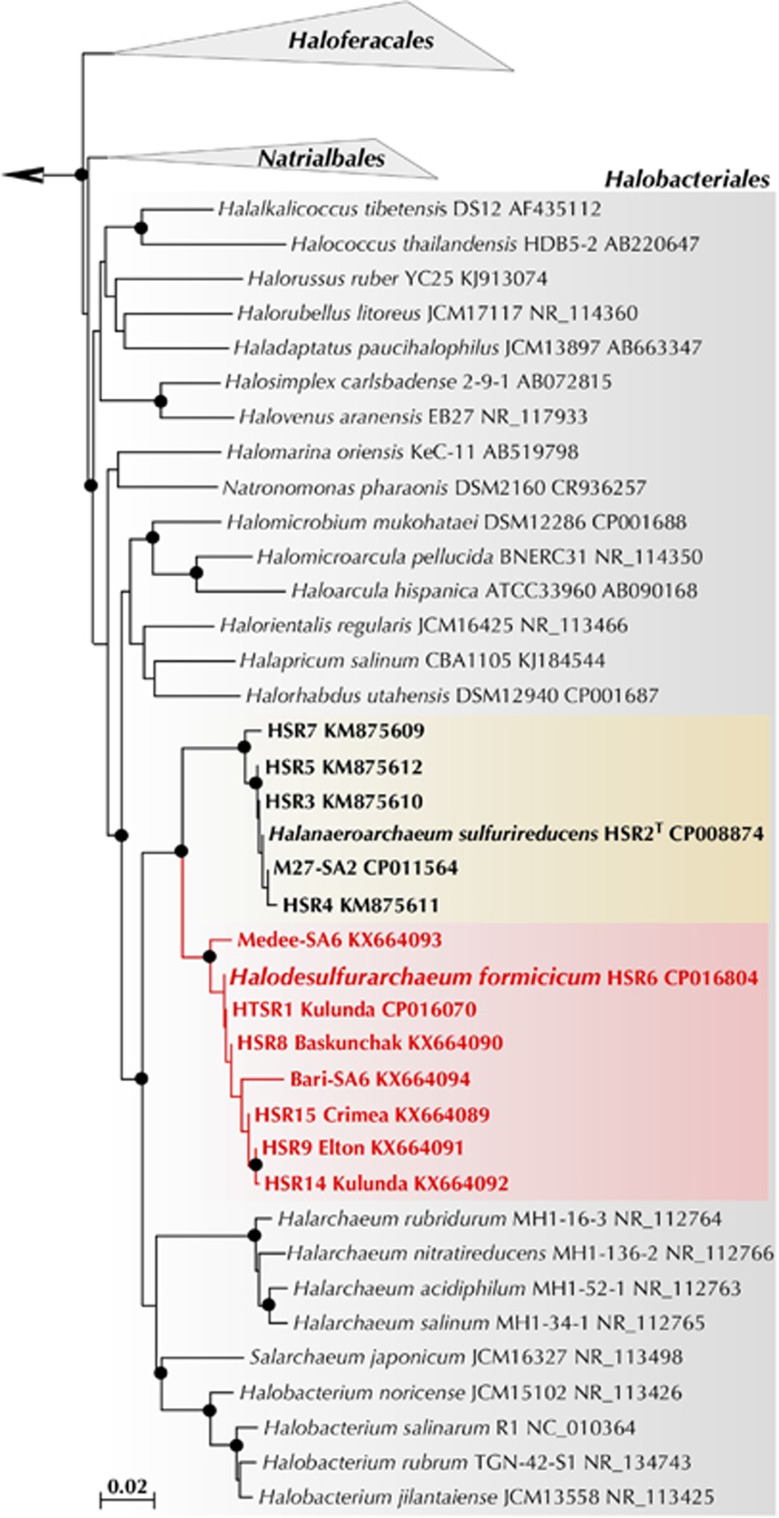
Phylogenetic position of the proposed genus *Halodesulfurarchaeum* within the order *Halobacteriales* inferred from a 16 S rRNA gene sequence alignment with PAUP*4.b10 using a LogDet/paralinear distance method as it described elsewhere (Sorokin *et al.*, 2016a). A phylogenetic tree based on 16 S rRNA gene sequences from members of the class *Halobacteria* covering all known genera (Gupta *et al.*, 2016). The members of orders *Natrialbales*, and *Haloferacales* are collapsed. The members of sulfur-respiring genera *Halanaeroarchaeum* and *Halodesulfurarchaeum* are highlighted in yellow and orange, respectively. 16 S rRNA gene phylogeny of the HDA strains was Support for nodes in this tree corresponds to bootstrap values for 1000 pseudo-replicates. Only bootstrap values at nodes greater than 75% are displayed as solid circles. The tree has been arbitrarily rooted with the sequences from *Methanohalophilus halophilus* (FN870068) used for out-grouping.

Phylogenetic analysis revealed that all 8 isolates are closely related to each other (98.4–100% 16S rRNA gene identity) and form a novel genus-level branch within the order *Halobacteriales* (Gupta *et al.*, 2016). We propose to classify this novel group as a new genus and species *Halodesulfurarchaeum formicicum* (HDA). The members of this genus clustered with the acetate-oxidising *Halanaeroarchaeum* strains (HAA) (95.3–95.5% identity), forming a separate clade of obligate anaerobic sulfur-respiring haloarchaea (Figure 1). This novel clade seems to be widely distributed across the globe and can be found in numerous microbially-explored anoxic hypersaline environments: solar salterns, high-altitude and flatland salt lakes of America, Central Asia and Europe and in the deep-sea anoxic brine lakes of Mediterranean Sea and Gulf of Mexico (Supplementary Figure S2). Noteworthy, sulfur-respiring haloarchaea are likely of a significant ecological importance since they represent a predominant group (up to 20% of the total population) in a variety of hypersaline ecosystems worldwide (Supplementary Table S1).

### Cell morphology and physiological characterisation

**Figure 2:**
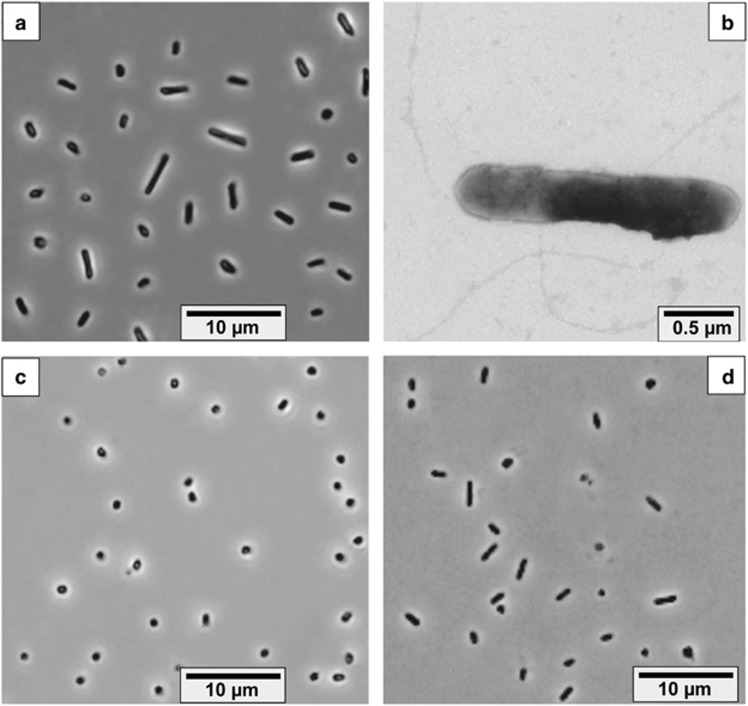
Cell morphology of four different *Halodesulfurarchaeum* isolates. Phase contrast microphotographs: (**a**) strain HSR6 (formate+S^0^); (**c**) strain HTSR1 (formate+thiosulfate); (**d**) strain HTSR14 (hydrogen+S^0^). Transmission electron microscopy (**b)** shows flagellation of cells of the strain HSR6.

Cells of the HDA strains isolated on formate and elemental sulfur had very similar cell morphologies, predominantly long flattened rods, which were actively motile with peritrichous archaella. Two strains, HTSR1 and HSR14, while growing on formate+thiosulfate and hydrogen+S^0^, respectively, had much smaller cells (0.6 × 1.0 μM) than the cells grown on formate+S^0^ (1.0–1.3 × 1.5–5.0 μM) (Figure 2).

Growth tests including other electron donors (acetate, ethanol, pyruvate, lactate, propionate, butyrate, butanol, succinate, glucose, fructose, ribose, glutamate and yeast extract) with sulfur as electron acceptor were negative for all isolates. No growth was obtained for all cultures while using arsenate, ferrihydrite, nitrate, nitrite, manganese dioxide, selenate, selenite, sulfate, sulfite and tetrathionate as alternative electron acceptors with formate as electron donor. Disproportionation of sulfur, thiosulfate and sulfite was negative. All strains were able to use DMSO as the electron acceptor with formate as the electron donor reducing it to DMS. DMSO was toxic to all strains at concentrations above 10 mM. A cross check of all isolates for the capability of hydrogenotrophy demonstrated that only HSR14 and HTSR1 strains were able to use H_2_ with sulfur as the electron acceptor. Only HTSR1, HSR8 and HSR9 strains could use thiosulfate as electron acceptor with formate as electron donor. Therefore, it might be concluded, that despite their close phylogenetic relation, the obtained isolates possess slightly different (but functionally important) phenotypes and are likely adapted to specific catabolic conversions.

We performed the further in-depth characterisation of the HSR6 and HTSR1 isolates, which, within the group of lithoheterotrophic haloarchaea, represent two metabolic extremes, the narrowest and broadest, correspondingly. The NaCl range for growth and sulfidogenic activity was characteristic for extreme halophiles with the growth range from 2.5 to 5.0 M (optimum at 3.5–4.0 M) and sulfidogenic activity range from 1.5 to 5.0 M (optimum at 3.5–4.5 M). Both strains grew with the couple formate/S^0^, but there was a substantial difference in growth dynamics and maximum sulfide formation between them (Supplementary Figure S3). Maximum amount of sulfide recovered in HSR6 culture was 14 mM with a concomitant consumption of 15 mM formate from 30 mM supplied, well corresponding to the 2 electron reduction of zero-valent sulfur. No further growth of HSR6 was observed likely due to the inhibition by accumulating sulfide. Apart from sulfide, a trace presence of volatile organic sulfur compounds, methanethiol (CH_3_SH) and carbon disulfide (CS_2_), at concentrations of 81 and 11.5 ppmv, respectively, were detected in the gas phase of the HSR6 culture at the stationary growth phase. In a control medium with 10 mM sulfide added initially, CH_3_SH and CS_2_ concentrations were also detected but at significantly lower concentrations (13 and 2 ppmv, respectively). This allows to conclude on the biological origin of, at least, CH_3_SH.

The anaerobic growth rate and biomass yield of HTSR1 with thiosulfate and formate, a type of respiration never seen before in haloarchaea, was comparable to that of HSR6 on elemental sulfur (Supplementary Figure S3). Although, the sulfide production by HTSR1 was significantly lower during growth on thiosulfate. Judging from a nearly 1:1 stoichiometry between the consumed formate and thiosulfate and produced sulfide,


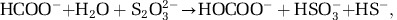


equimolar amount of thiosulfate was reduced to sulfide and sulfite. Sulfite formation was confirmed analytically. The experiments with washed cells (Supplementary Table S2) demonstrated that the cells of HSR6 and HTSR1 grown with S^0^, were active only with sulfur as electron acceptor, while the HTSR1 cells grown on thiosulfate were equally active both with thiosulfate and sulfur as electron acceptors. The cells of both strains grown with DMSO were active with DMSO and elemental sulfur, while no respiration with thiosulfate was observed.

### General genomic features of HSR6 and HTSR1 strains

We determined complete genome sequences of the HSR6 and HTSR1 strains (most of the data are present in Supplementary Tables S3–S9 and Supplementary Figures S4–S5). Both genomes were single circular replicons of 1,972,283 bp (HTSR1) and 2,085,946 bp (HSR6), with GC molar content of 63.76 and 63.62%, respectively. Both genomes harbour a single rRNA operon and 45 tRNA genes. Genomes exhibited the average nucleotide identity (ANI) 98.58%, while containing several strain-specific genomic regions: one in HTSR1 and four in HSR6. The unique island ‘D’ in HSR6 genome encodes CRISPR and CRISPR-associated proteins (Deveau *et al.*, 2010). Following the current classification, HSR6 CRISPR-Cas was affiliated to I-B (*E. coli*) or CASS7 (Makarova *et al.*, 2011). Using the ACLAME database (Leplae *et al.*, 2010), spacer sequences were blasted against Plasmid, Virus and Prophages databases using ACLAME web site tool (http://aclame.ulb.ac.be/) with default parameters. Only spacer #38 was found distantly related (1e-03) to phage-related DNA polymerase (NC_004556). Remarkably, HSR6 and HSR2 showed no homology between the spacer sequences, likely implying a different history of phage interaction for the strains, regardless of their isolation from the same environments.

### Energy generation and proton-translocation machinery

In addition to the use of elemental sulfur, the HDA strains demonstrated their capability to utilise DMSO and thiosulfate as terminal electron acceptors, which are the predominant products of corresponding oxidation of dimethylsulfoniopropionate and sulfide in saline environments (Jørgensen, 1990; Matsuzaki *et al.*, 2006). The HTSR1 genome encodes 10 molybdopterin oxidoreductases from CISM superfamily (Duval *et al.*, 2008) that may be potentially involved in the central catabolic reactions (Supplementary Table S9–S11). To facilitate comparison of these enzymes in various strains, we established their sequential numeration, based on appearance in the HTSR1 genome. This set exceeds by 2.5-fold the numbers of corresponding enzymes in the acetate-oxidising sulfur-reducing HAA (Sorokin *et al.*, 2016a), which coheres with the advanced metabolic versatility of the novel group of anaerobic haloarchaea. Noteworthy, HSR6 differs from HTSR1 by the absence of only one molybdopterin oxidoreductase, HTSR_0625-0630, which, taking into account the inability of HSR6 to grow on thiosulfate, seems to play a pivotal role in the utilisation of this compound as an electron acceptor.

**Figure 3:**
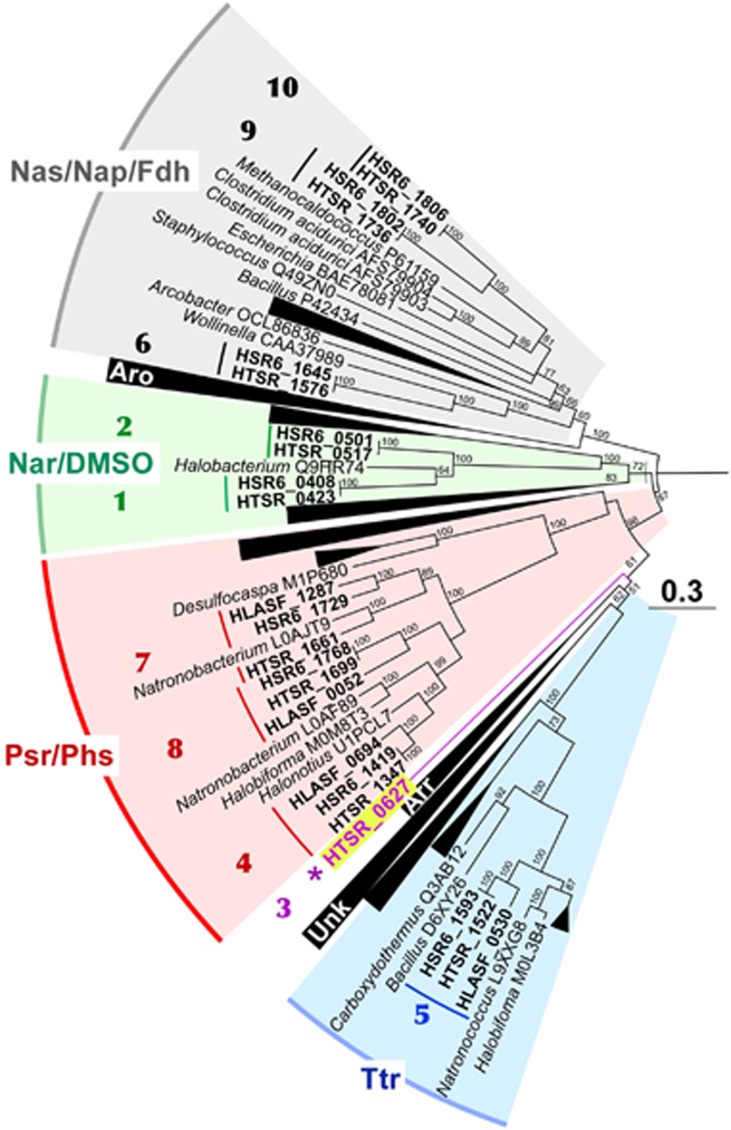
Maximum Likelihood phylogenetic tree of CISM catalytic subunits A. Totally 168 sequences were taken for the analysis. The tree with the highest log likelihood (-132625.7976) is shown. The bootstrap values (100 replicates) are shown next to the branches. All positions with less than 95% site coverage were eliminated. There were a total of 580 positions in the final dataset. The tree was constructed in MEGA6 (Tamura *et al.*, 2013). CISM proteins of three sulfur-reducing haloarchaea *Halanaeroarchaeum sulfurireducens* HSR2^T^ (HLASF) and *Halodesulfurarchaeum formicicum* strains HTSR1 and HSR6 are highlighted in bold (locus tag prefixes are HLASF, HTSR and HSR6, respectively). Sequential numeration of all HDA CISMs is used as in Table S10. Abbreviations used: Aro, arsenite oxidases family; Arr, arsenate reductase family; Nar/DMSO, nitrate/DMSO reductase family; Nas/Nap/Fdh, assimilatory (periplasmic) nitrate reductase/formate dehydrogenase family; Ttr, tetrathionate reductase family; Psr/Phs, polysulfide/thiosulfate reductase family; Unk, unknown family. Bar is 0.3 aminoacid substitutions per site.

The phylogenetic analysis of detected CISMs suggested they can exhibit various activities (Figure 3). To improve the inference, we tried to assign the activity of detected molybdopterin oxidoreductases by using the physiological data. Molecular basis of formate-dependent respiration with sulfur compounds likely relies upon three respiratory dehydrogenases and seven terminal reductases. All strains of sulfur-reducing haloarchaea with currently completed genomes (HSR2, M27-SA2, HSR6 and HTSR1) possess four common CISM oxidoreductases. One of them belongs to the haloarchaeal branch of tetrathionate (Ttr) reductase family (Duval *et al.*, 2008), while three others are the members of polysulfide/thiosulfate (Psr/Phs) reductases (Figure 3). HAA strains do not grow on DMSO as electron acceptor, obviously due to lack of two enzymatic complexes present in both HDA genomes, which belong to DMSO/trimethylamine-N-oxide (TMAO)/nitrate reductases (NAR) family. The nearest characterised enzyme, (Q9HR74), is a DMSO/TMAO reductase from *Halobacterium salinarum* (Müller and DasSarma, 2005), which makes the assignment of their metabolic function more reliable. Noteworthy, all these DMSO reductases form a deep branch within nitrate reductases cluster Nar and likely represent an ancient form of DMSO-reducing enzymes. HTSR1 has a unique deeply branched CISM oxidoreductase (HTSR_0627), missing in all genomes of S^0^-reducing haloarchaea. We could not affiliate this enzyme with any known CISM family. However, despite that it does not fall into the Psr/Phs family, its function seems to be related with thiosulfate reduction since this enzyme was not found in other genomes and the capability to grow on thiosulfate as an electron acceptor is the key physiological feature of HTSR1.

Inspection of the HDA genomes revealed that formate metabolism in *Halodesulfurarchaeum* is considerably diversified, pointing at a great importance of formate for these organisms in sustaining the life. These haloarchaea are evolved to exploit the low reduction potential of formate (E_0_’ [CO_2_/HCOO^−^]=−430 mV (Thauer *et al.*, 1977) to derive the energy by coupling its oxidation to the reduction of various electron acceptors. To facilitate these physiological roles, different types of formate dehydrogenase (Fdh) enzymes, both membrane-anchored and cytoplasmic, are present in the HDA genomes. The first type of Fdh coded by an operon of membrane-bound peripherally oriented formate dehydrogenase (HTSR_1573-1576), resembled that of a thoroughly investigated bacterial analogue *Wolinella succinogenes* (Figure 3). In *W. succinogenes*, membrane-bound Fdh is suggested to be involved in the periplasmic dissimilatory reduction of nitrite to ammonia and elemental sulfur to sulfide, catalysed by corresponding reductases Nrf and Psr, respectively (Simon, 2002; Simon and Klotz, 2013). By analogy, the electron transfer chain of the HDA strains has to possess membrane menaquinone pool that mediate transfer of electrons between membrane-bound respiratory Fdh dehydrogenase and terminal reductases. It is very likely that this transfer is coupled to the consumption of protons from the inside and to proton release on the outside of the membrane, thus contributing to the proton motive force generation. The genomes of HDA strains contain 10 (methyl)menaquinone biosynthetic genes located in four loci: two separate *ubiE* genes (HTSR_1082 and 1583), the *menA*-*ubiE* (HTSR_1105-1106) and the *menFDBACE* (HTSR_1290-1295) clusters. Such gene array unambiguously points at the occurrence of the classical menaquinone biosynthetic pathway via isochorismate, o-succinylbenzoate and 1,4-dihydroxy-2-naphthoate (Chen *et al.*, 2013; Zhi *et al.*, 2014). All predicted HDA menaquinone biosynthetic proteins were remarkably similar (42–75% of identity) with the corresponding proteins of *Natronobacterium gregoryi*, whose menaquinone composition is known. This haloarchaeon possesses four major respiratory quinones corresponding to unsaturated and VIII-dihydrogenated menaquinones and methylmenaquinones with 8 isoprene units [MK-8, MK-8(VIII-H_2_), MMK-8 and MK-8(VIII-H_2_)] (Collins and Tindall, 1987).

Cytoplasmic type of Fdh is coded by two copies of genes for FdhA catalytic subunits (HTSR_1736 and 1740), that are located in close vicinity. As it is typical for cytoplasmatic Fdh (Maia *et al.*, 2015), we did not find genes, encoding other subunits present in membrane-bound CISM oxidoreductases complexes. Phylogenetic analysis revealed their similarity to cytoplasmic catalytic subunits FdhA of archaeal (*Methanococcus*) and bacterial (Clos*tridium acidurici*) lineages (Figure 3). As it was proposed for some formate-utilising methanogens (Wood *et al.*, 2003), cytoplasmic FdhA is required to oxidise formic acid to CO_2_ and to generate reduced electron carriers for energy conservation. To interact with the electron acceptors, cytoplasmic FdhAs of HDA need an appropriate ‘interface’ to use ferredoxins and NAD. The genes located next to the cytoplasmic *fdh*A operon could encode this putative ‘interface’, namely, the electron transfer flavoprotein EtfAB (HTSR_1748-49). Apparently, this flavin-containing module can be combined with the FdhA to perform energy conservation by coupled reduction of ferredoxin and NAD^+^ via mechanism of flavin-based electron bifurcation (Buckel and Thauer, 2013):


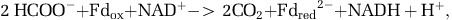


just as it was documented for the first bifurcating formate dehydrogenase of *Clostridium acidurici* (Wang *et al.*, 2013).

**Figure 4:**
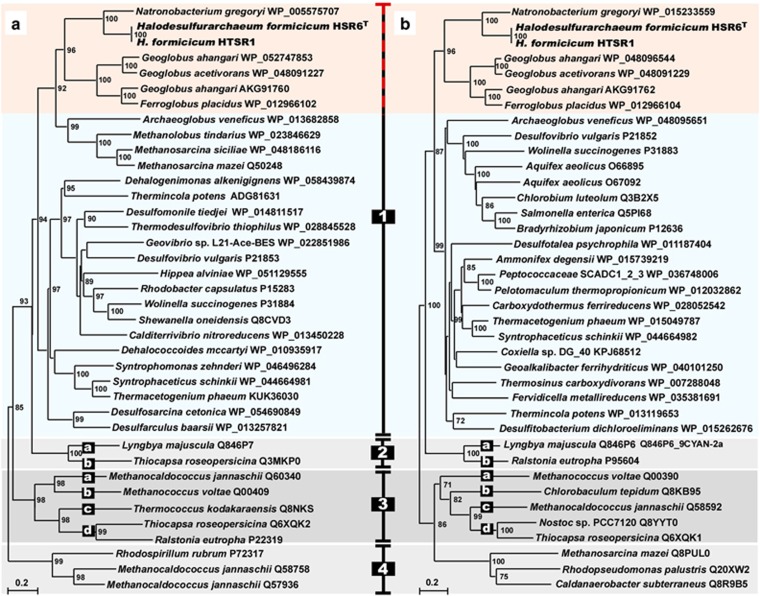
Phylogenetic tree of [NiFe]-hydrogenases constructed with full-length enzymes from small HydA (**a**) and large HydB (**b**) subunits of subgroup representatives. Based on the report Vignais and Billoud (2007), the alignment was made with Clustal W584 and MacVector 11.1.2. Trees were computed with PhyML586 using the bootstrap procedure with 1000 replicates and bootstrap values of more than 700 (70%) are displayed as percentages close to the corresponding nodes. The nodes are displayed so that the corresponding small and large subunits can be read in the same top-down order. Branch lengths along the horizontal axis reflect the degree of relatedness of the sequences (20%).

**Figure 5:**
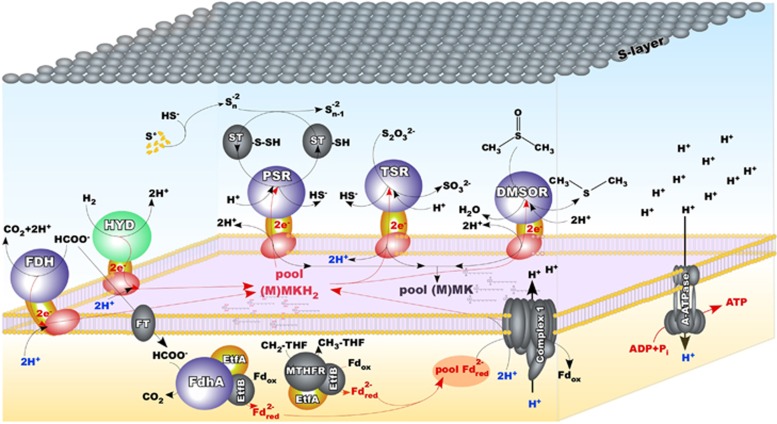
Proposed pathways for energy generation and proton-translocation machinery in *Halodesulfurarchaeum*. Molybdopterin- and [Ni-Fe]-containing catalytic subunits of respiratory complexes are shown in blue and green, correspondingly. Subunits, that transfer electrons and predicted to possess four iron-sulfur centers, are shown in yellow, while integral membrane subunits, that anchor the other two subunits to the membrane and predicted to contain the site for MH_2_ oxidation and two heme cofactors, are shown in red. Abbreviations: A-ATPase, archaeal ATP synthase; CH_2_-THF, methylene-tetrahydrofolate; CH_3_-THF, methyl-tetrahydrofolate; DMSOR, DMSO reductase; Etf, electron transfer flavoprtein; FDH, formate dehydrogenase; FT, formate transporter; HYD, uptake hydrogenase; (M)MK, oxidised (methyl)menaquinone; (M)MKH_2_, reduced (methyl)menaquinone; MTHFR, 5,10-methylene-tetrahydrofolate reductase; PSR, polysulfide reductase; ST, sulfurtransferase; TSR, thiosulfate reductase.

Aforementioned physiological studies revealed, that besides formate HTSR1 can use hydrogen, a well-known electron donor utilised for microbial litho(auto)trophic growth. Before our findings, utilisation of formate and H_2_ as electron donors was not observed in any known species of the class *Halobacteria*. Strain HTSR1 has a gene cluster HTSR_0658-0657 encoding [NiFe]-hydrogenase, consisting of three subunits, HydA (39.3 KDa), HydB (55.3 KDa) and HydC (37.4 KDa). Noteworthy, the HSR6 strain possesses identical [NiFe]-hydrogenase gene cluster, but fails to use hydrogen as the electron donor. Phylogenetic analysis of full-length subunits HydA and HydB of HDA revealed that they belong to Group 1 of the [NiFe]-hydrogenases (Figure 4). The hydrogenases in Group 1 are known as membrane-bound, respiratory uptake hydrogenases capable of supporting growth with H_2_ as an energy source (Vignais *et al.*, 2001; Vignais and Billoud, 2007). Although the exact mechanism for the generation of the electrochemical proton gradient with formate or H_2_ as electron donors is yet to be elucidated in HDA, both membrane-bound Fdh and Hyd could reduce menaquinone with formate/H_2_ with concomitant transfer of electrons to terminal reductases and outward proton pumping (Figure 5).

Besides aforementioned enzymes, the Complex 1-like oxidoreductase (HTSR_1171-1181) is the supplementary component of proton-translocation machinery in HDA. Similar to HAA and other obligate anaerobes (Castelle *et al.*, 2013; Probst *et al.*, 2014; Sorokin *et al.*, 2016a), this complex lacks the NADH-binding module and is hypothesized to use reduced ferredoxin as the electron donor for the generation of the proton gradient (Battchikova *et al.*, 2011). We propose, that one of the sources of reduced ferredoxins is the cytoplasmic oxidation of formate by the monomeric FdhA. The proton gradient can be further utilised by the V-type archaeal H+-ATP synthase complex (HTSR_1802-1811) for the generation of ATP, thus providing an attractive mechanism for efficient energy conservation in *Halodesulfurarchaeum* (Figure 5).

### One-carbon metabolism

**Figure 6:**
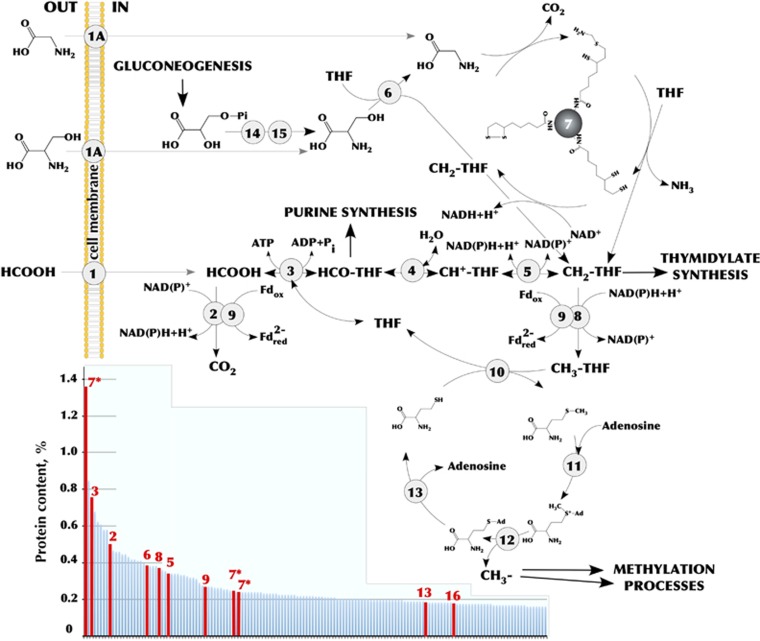
Summary of C_1_ metabolism in *Halodesulfurarchaeum formicicum* HTSR1 representing ancestral routes of glycine, serine and methyl group chemistry (Braakman and Smith, 2012). Pathways shown in the model were deduced on the basis of genome annotation and genome-wide proteomic analysis of cells grown on different electron acceptors. Enzymes involved: (1) formate transporter (HTSR_0446) or formate/oxalate antiporter (HTSR_1713); (1 A) numerous amino acid permeases; (2) formate dehydrogenase subunit alpha (HTSR_1736 [A], 1740 [B]); (3) formate—tetrahydrofolate ligase (HTSR_1739); (4) 5,10-methylenetetrahydrofolate reductase (HTSR_1746); (5) methylenetetrahydrofolate dehydrogenase (NADP+) (HTSR_1747); (6) serine hydroxymethyltransferase (HTSR_0671); (7) glycine cleavage system proteins H, P and T (HTSR_0503-0506, 1750); (8) methylenetetrahydrofolate reductase (NADPH) (HTSR_1220); (9) electron transfer flavoprotein (HTSR_1748-1749); (10) methionine synthase (HTSR_1805); (11) S-adenosylmethionine hydroxide adenosyltransferase (HTSR_1447); (12) S-adenosylmethionine-dependent methyltransferase (HTSR_1450); (13) adenosylhomocysteinase (HTSR_0160); (14) D-3-phosphoglycerate dehydrogenase (HTSR_0539), (15) phosphoserine phosphatase (HTSR_1388). The data for the 150 most abundant proteins from proteomic analysis are outlined in the small nested box. The proteins are sorted according to their relative abundance in cells grown on formate+thiosulfate. Proteins involved in C_1_ metabolism are indicated in red. Abbreviations: Fd, ferrodoxin; THF, tetrahydrofolate.

The extra- and intracellular oxidation of formate is the key catabolic property of novel haloarchaea and of utmost interest since it had never been observed in any known haloarchaeal species. Apart from three CISM formate dehydrogenases, both HDA genomes harbour the full set of genes encoding the tetrahydrofolate (THF)-dependent enzymes involved in the reversible conversion of formate to methyl-THF (Figure 6). The corresponding enzymes likely provide C_1_-units for purine and thymidylate synthesis, similarly to the euryarchaeon SM1 and *Methanosarcina barkeri* (Buchenau and Thauer, 2004; Probst *et al.*, 2014). The other major requirement for C_1_-units comes from the provision of methyl groups for multiple biosynthetic methylation reactions (Brosnan *et al.*, 2015). The HDA genomes encode the full set of enzymes needed to catabolise methionine via its conversion to *S*-adenosylmethionine (SAM) with the transfer of SAM methyl group to a substrate for methylation, producing *S*-adenosylhomocysteine (SAH) and the methylated substrate (Figure 6). In accordance with this route, we found more than 35 different methyltransferases including two SAM-dependent methyltransferases (HTSR_1450, 1509). It seems that beside these two canonical biosynthetic functions of one-carbon metabolism (methylation reactions and purine/thymidylate synthesis), the third metabolic function is also operative in HDA cells: serine and glycine metabolism via glycine cleavage system (Figure 6). In case these substrates can provide HDA cells with more one-carbon groups than they need, the oxidative conversion of methylene-THF back to formate and ultimately to carbon dioxide might be a mechanism for their disposal. As proposed by Brosnan *et al.* (2015), this process in addition to oxidizing the excess of C_1_-units can reduce substantial quantities of NADP^+^ to NADPH and produce ATP.

Noteworthy, before entering in SAM-cycle, methylene-THF is reduced to methyl-THF, which is highly exergonic reaction using NADH. This reductant produced by cytoplasmic oxidation of formate can be used by methylenetetrahydrofolate reductase, which was suggested to be involved in energy conservation by reducing ferredoxin via electron bifurcation (Hess *et al.*, 2013). Thus, the one-carbon metabolism in HDA likely has the fourth metabolic function—to fuel the bioenergetic coupling site via NADH-dependent methylene-THF reduction (Figure 6).

### Heterotrophy

Anoxic sediments of hypersaline lakes and salterns receive a variety of forms of detrital organic matter from the overlying compartments, which provide carbon and nitrogen to anaerobic microbial communities. Consistently with current insight (Oren, 2011; Yakimov *et al.*, 2013), the anaerobic metabolic diversity at the highest salinities is poor due to energetic constraints and is restricted primarily to fermentation, methylotrophic methano- and acetogenesis and recently discovered acetoclastic sulfur reduction (Sorokin *et al.*, 2016a). HDA strains represent a novel type of haloarchaeal anaerobic metabolism, which is operative at the highest salinities, i.e. hydrogen/formate-dependent lithoheterotrophy. As mentioned above, the presence of yeast extract is essential for growth of all HDA strains. According to this, two oligopeptide/dipeptide ABC transporters and 9 transporters for amino acids were found along with 22 cytoplasmic and membrane-associated proteases and peptidases (Supplementary Table S9). The genome inspection of HDA strains revealed the synthesis pathway for lysine was incomplete, pointing at the dependence of HDA on external sources of this amino acid. In accordance with the cultivation tests, sugars cannot be used by HDA, likely due to the lack in the genomes of hexokinase and phosphofructokinase, the enzymes initiating the glycolysis. The presence of an unidirectional fructose-1,6-biphosphate aldolase/phosphatase suggests that the metabolic fluxes are oriented in gluconeogenetic direction (Say and Fuchs, 2010) from pyruvate to phosphoenolpyruvate (via phosphoenolpyruvate synthase) and further to fructose-6-phosphate. At the same time, the well-developed routes for amino acids degradation are encoded by both HDA genomes confirming their capacity for using these compounds as the sole carbon sources, ultimately catabolising those via the tricarboxylic acid (TCA) cycle. Most proteins involved in the canonical oxidative TCA cycle are encoded in HDA genomes, except for 2-oxoglutarate dehydrogenase, which is, as in case with HAA, replaced by 2-oxoglutarate:ferredoxin oxidoreductase.

Each of the HDA genomes possessed genes for two ADP-forming acetyl-CoA synthetases that have been proven to catalyse the acetate production in various archaea (Glasemacher *et al.*, 1997; Musfeld *et al.*, 1999). The presence of this enzyme suggests that acetyl-CoA, produced via deamination and subsequent oxidation of amino acids, aside from entering in TCA cycle, could be converted to acetate, thus generating one ATP by substrate level phosphorylation. We are aware of this assumption and despite the apparent advantage of this reaction, the production of acetate in traceable amount was not detected in formate-growing HDA cultures (data not shown). Unlike the acetoclastic HAA, none of the genes encoding for enzymes of the glyoxylate shunt, which allows acetate to be used as the sole carbon source, were identified in HDA genomes. Additionally, none of the genes associated with the methyaspartate cycle, an alternative pathway of acetate assimilation in certain haloarchaea (Khomyakova *et al.*, 2011), were found. Therefore, we believe that if acetate is produced, it is likely excreted as an end-metabolite by formate/oxalate antiporter, which is simultaneously involved in uptake of formate (Probst *et al.*, 2014). As far as acetate is the key substrate for acetoclastic *Haloanaeroarchaeum* (Sorokin *et al.*, 2016a), it can be an important link between these haloarchaea. Hence the potential ability of HDA to generate acetate could greatly influence the terminal anaerobic degradation cascade of organic matter in hypersaline ecosystems.

### Energy metabolism confirmed by comparative proteome analysis

**Figure 7:**
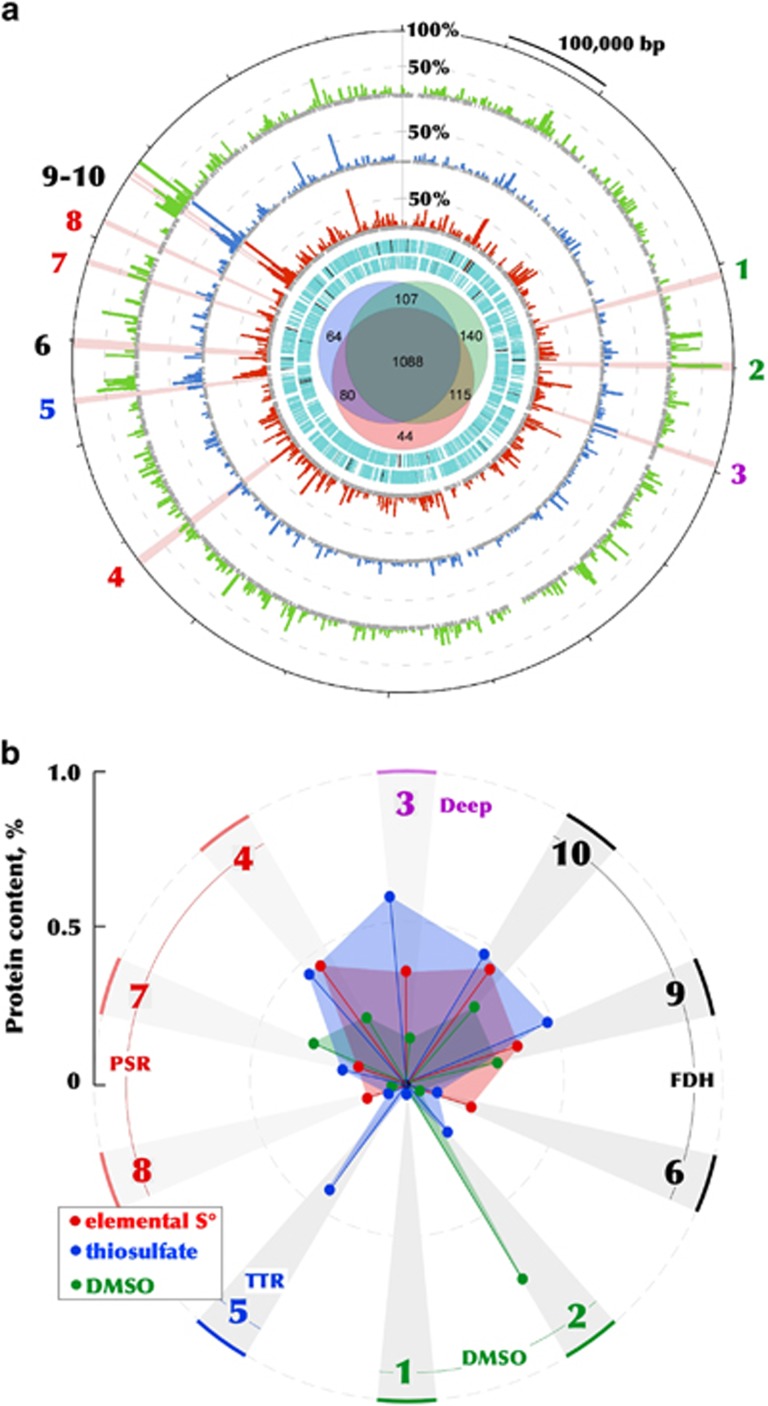
The 1.97-Mbp genome and differential proteome of *Halodesulfurarchaeum formicicum* HTSR1. (**a**) The outermost ring indicates the position on the genome map of the 10 sequentially numbered CISM enzymatic complexes (as in Figure 3 and Table S10), including two DMSO reductases DMSOR (1, 2), one unaffiliated CISM complex ‘Deep’ (3), three polysulfide reductases PSR (4, 7, 8), one thiosulfate reductase TSR (5) and three formate dehydrogenases FDH (6, 9-10). The second, third and fourth rings (histograms) are the relative abundances of proteins detected in corresponding proteomes, normalized versus the most abundant protein in all three proteomes, glycine cleavage system protein T (HTSR_1750, 100%). Two innermost cyan rings indicate predicted ORFs on the plus and minus strands, respectively. The Venn diagram in the centre shows the numbers of proteins detected in sulfur- (red), thiosulfate- (blue) and DMSO-respiring (green) cells. (**b**) Relative abundances of the 10 sequentially numbered CISM enzymatic complexes identified in corresponding proteomes. Key to protein annotations: 1. DMSOR (catalytic subunit HTSR_0423); 2. DMSOR (catalytic subunit HTSR_0517); 3. Deeply branched CISM (catalytic subunit HTSR_0627); 4. PSR (catalytic subunit HTSR_1347); 5. TSR (catalytic subunit HTSR_1522); 6. FDH (catalytic subunit HTSR_1576); 7. PSR (catalytic subunit HTSR_1661); 8. PSR (catalytic subunit HTSR_1699); 9. FDH (catalytic subunit HTSR_1736); 10. FDH (catalytic subunit HTSR_1740). Relative abundances of all proteins identified in the global proteome is provided in Supplementary Table S11.

In the present work, the proposed metabolic pathways during respiration with different terminal acceptors, were analysed through the proteome assays (Figure 7). Although we did not aim here to perform the detailed comparative analysis of HTSR1 proteomes, the description of the peptide-level scoring metrics is provided in Supplementary Table S11 and in Supplementary Discussion. It must be specified that inspection of the proteome revealed that the proposed C_1_ metabolism in HDA is active, since eleven enzymes of the pathways depicted on Figure 6 were among the most abundant proteins.

The experiments with washed cells demonstrated that the formate/S^0^ grown HTSR1 cells were active only with sulfur, while no reduction of either thiosulfate or DMSO was detected (Supplementary Table S2). Consistently with this observation, the analysis of expressed CISM complexes revealed that neither DMSO reductases (HTSR_0423 and 0517), nor the putative thiosulfate reductase (HTSR_1522) were induced during the growth on elemental sulfur (Figure 7b). In contrary, all three polysulfide reductases, together with the ‘Deep’ CISM were induced. This observation confirms that they are essential components of the energy production machinery during sulfur respiration with formate. Among the last group of enzymes, the PsrA HTSR_1347, co-transcribed together with periplasmatic sulfurtransferase/rhodanese-like protein HTSR_1348, was the most abundant, i.e. was 3–4 times higher than the other Psr reductases. This finding corroborates with the significance of sulfurtransferase in transformation of practically insoluble S^0^ into soluble polysulfide, thus functioning as the sulfur supplier for the catalytic subunit of Psr reductases (Klimmek *et al.*, 1998; Hedderich *et al.*, 1999; Campbell *et al.*, 2009; Aussignargues *et al.*, 2012), which was also the case in HAA (Sorokin *et al.*, 2016a).

Washed cells of HTSR1 grown with formate/thiosulfate were equally active with both thiosulfate and sulfur as terminal electron acceptors, but not with DMSO (Supplementary Table S2). Correspondingly, the total protein expression profile of thiosulfate-grown cells was very similar to that of sulfur-respiring cells, with only few differences observed. First of all, thiosulfate induced the expression of HTSR_1522 reductase of the Ttr family, repressed in other HTSR1 proteomes, and significantly increased (68%) the abundance of unaffiliated CISM ‘Deep’ reductase (HTSR_0627). Another remarkable difference with S^0^-grown cells is that the utilisation of thiosulfate as terminal electron acceptor was accompanied by a 2.5-fold decrease in the abundance of membrane-bound formate dehydrogenase HTSR_1576 with a simultaneous increase in the abundance of cytoplasmic FdhA dehydrogenases HTSR_1736 and HTSR_1740. A notable aspect of the 2-electron reduction of thiosulfate is that under standard conditions, the reduction potential (E°’) of the S_2_O_3_^2−^/(HS^−^+SO_3_^2−^) electron acceptor couple is -402 mV, which is considerably lower than that of the MK/MKH_2_ electron donor couple (-74 mV), resulting in an unfavourable *Δ*E°= of -328 mV for the reaction. The principle, that the reduction potentials of cleavage reaction are considerably higher under physiological conditions (Stoffels *et al.*, 2012), can diminish unfavorable *Δ*E°, but anyhow the two-electron reduction catalysed by thiosulfate reductase has to be linked to an exergonic process in order to operate in the endergonic direction. Therefore, we assumed that, similarly to formate-dependent thiosulfate reduction in *Salmonella enterica* (Stoffels *et al.*, 2012), the proton motive force (PMF) is only just sufficient to drive the thiosulfate reductase reaction. Above we suggested a mechanism in which formate is oxidised in the cytoplasm to produce the additional Fd_red_. This reductant is further used by the ferredoxin:menaquinone Complex 1-like oxidoreductase (HTSR_1171-1181) to generate the extra PMF, which is likely necessary to overcome the unfavourable red-ox conditions of respiration with thiosulfate (Figure 5).

When the HTSR1 cells were grown with DMSO as the electron acceptor, they were active also with elemental sulfur, while thiosulfate was not used as the sulfidogenic substrate (Supplementary Table S2). This may indicate that the sulfur reduction is rather a constitutive phenotype of the HTSR1 strain. Proteomic data corroborates with physiological testing, whereby the strong induction of DMSO reductase HTSR_0517 was observed (Figure 7b). Noteworthy, the second DMSO reductase (HTSR_0423) was not found among the expressed proteins, indicating that this reductase did not contribute to the respiration activity of the HTSR1 strain. During the growth with DMSO, the proton-translocation machinery in HDA does not seem to require a significant amount of the membrane-bound Fdh reductase, judging from its decreased expression (6.5-fold lower than in sulfur-respiring cells).

Taken together, the results of differential proteome analyses and respiration experiments revealed that polysulfide reductases are constitutively expressed in HDA cells, indicating that elemental sulfur, despite being one of the energetically least favourable, serves as the preferential electron acceptor. The growth with other electron acceptors requires the induction of corresponding oxidoreductases. Thus, *Halodesulfurarchaeum* strains possess a remarkable adaptation machinery to thrive in hypersaline anoxic habitats, while exhibiting capacity of utilising low-potential electron acceptors at the thermodynamic edge of life.

### Classification

On the basis of phylogenetic and phenotypic properties we propose to classify the novel group of anaerobic haloarchaea described above as a novel genus and species *Halodesulfurarchaeum formicicum* gen. nov., sp. nov. within the family *Halobacteriaceae*.

#### Description of *Halodesulfurarchaeum* gen. nov

[hal.o.de.sul'fur. Gr.n. *hals*, *halos* salt of the sea; L. pref. *de*-, from; L.n. *sulfur*, sulfur; N.L. neut. n. *archaeum* archaeon from Gr. adj. archaios-ê-on ancient; N.L. neut. n. *Halodesulfurarchaeum* sulfur-reducing haloarchaeon].

Extremely halophilic, neutrophilic, obligately anaerobic euryarchaea growing by sulfur-dependent respiration with formate or hydrogen as electron donor, thereby representing a first example of haloarchaea with the lithotrophic metabolism. A member of the family *Halobacteriaceae*. Found in hypersaline chloride brines of terrestrial and marine origin. Recommended three-letter abbreviation: *Hda*. The type species is *Halodesulfurarchaeum formicicum*.

#### Description of *Halodesulfurarchaeum formicicum* sp. nov

[for.mi'ci.cum. N.L. neut. n. acidum formicicum, formic acid; L. neut. suffixicum, suffix used with the sense of belonging to, pertaining to; N.L. neut. adj. *formicicum*, pertaining to formic acid].

Cells are variable in shape and size at different growth conditions: from flattened motile rods 1.0–5.0 × 0.6–0.8 μM (growth on sulfur and DMSO) to small nonmotile cocci 0.6–0.8 × 1.0 μM (with thiosulfate). The cell wall consists of a thin proteinaceous layer. The cells lyse at salt concentration below 2M. Carotenoids are absent. The core membrane lipid analysis demonstrated a presence of two dominant components: archaeol (C_20_-C_20_ diglycerol ether [DGE], 40% of the total) and extended archaeol (C_20_-C_25_ DGE, 59% of the total). Trace presence (1.2% in total) of the monoglycerol ether (MGE) lipids (2-C_20_ MGE, 1-C_20_ MGE and 2-C_25_ MGE) was also detected. The phospholipids were dominated by phosphatidylglycerophosphate methylester, while phosphatidylglycerol, phosphatidylethanolamine and two unidentifyed C_45_/C_40_ lipid species were less abundant. Obligately anaerobic, growing by elemental sulfur and DMSO (all strains) or thiosulfate (some strains) respiration with either formate (all strains) or hydrogen (some strains) as electron donor. Sulfur is reduced to sulfide with intermediate formation of polysulfides and traces of organic sulfides, such as methanethiol and CS_2_. Polysulfides_(S4-S7)_ are formed at concentrations up to 1mM. Some strains are capable of incomplete thiosulfate reduction to sulfide and sulfite, while DMSO is reduced to DMS. Yeast extract can serve as carbon source, but not as energy source. Ammonium is utilised as N-source. Optimum growth temperature is 37 °C (maximum at 50 °C). Extremely halophilic, with the range of NaCl for growth from 2.5 to 5M (optimum at 3.5–4 M), and neutrophilic, with the pH range for from 6.5 to 8 (optimum at 7.0–7.2). The G+C content of genomic DNA in the type strain HSR6 is 63.62 mol%. Habitats: hypersaline lakes and solar salterns. The type strain (HSR6^T^=JCM 30662 ^T^=UNIQEM U983^T^) was isolated from mixed anaerobic sediments of hypersaline chloride-sulfate lakes in Kulunda Steppe (Altai, Russia).

## Conclusions

This study has demonstrated that even the well-studied microbial habitats could reveal a significant new knowledge on novel microbial taxa and their metabolism, by applying a hypothesis-driven combination of cultivation, physiological and in-depth ‘omic’ analyses. We have discovered and characterised a novel lifestyle of haloarchaea, prevalent in anoxic hypersaline systems worldwide, yet very different from that of all previously described members of the class *Halobacteria*. We proposed the new genus, *Halodesulfurarchaeum,* within family *Halobacteriaceae* to accommodate this new lineage. We further propose that, along with recently described genus *Halanaeroarchaeum*, this new genus partitions the class *Halobacteria* into distinct phenotypes, consisting of aerobic (with the exception of few facultative anaerobes) and obligate anaerobic organisms. Evidence supporting last proposal includes: (i) lineage-specific features, such as acetotrophy and lithoheterotrophy coupled with the previously overlooked type of sulfur-dependent respiration and (ii) significant intra-lineage diversity and abundance within geographically distinct hypersaline habitats worldwide. The sister grouping of ‘anaerobic’ and ‘aerobic’ haloarchaea reflects their plausible derivation from an ancient common aerobic halophilic ancestor. The ongoing metabolic diversification then resulted in subsequent divergence along separate evolutionary paths. A second possible scenario implies a consideration, that obligate anaerobic sulphur-reducing haloarchaea have a different evolutionary history than the aerobic counterparts and their ancestors avoided the massive lateral gene transfer event from aerobic bacteria.

In addition to the metabolic peculiarities, lineage–specific characteristics of ‘anaerobic’ haloarchaea, attributed to adaptation to anoxic habitats at the thermodynamic edge of life, include their compact genome and single-copy rRNA operon, rarely seen among haloarchaea. These features have been proposed to minimise metabolic costs in energy-limited habitats where neither broad metabolic repertoire nor high numbers of paralogous proteins are needed. The sporadic identification of sulfur-respiring ‘anaerobic’ haloarchaea (up to 20% of the total archaeal communities in Lake Tirez) in microbial surveys of hypersaline communities worldwide (Supplementary Figure S2 and Table S1) suggests that they represent so far overlooked but significant fraction of the biomass and diversity in these habitats. The inability of earlier studies to recognise their significant contribution to anaerobic part of sulfur and carbon cycling in hypersaline habitats is likely due to limitations in cultivation methods routinely used to assess the diversity of extreme halophiles. It is therefore not surprising that before our studies the list of hypersaline archaeal isolates described to date did not include any obligate anaerobes.

## Figures and Tables

**Table 1:** Growth characteristics of isolated lithoheterotrophic sulfur-respiring haloarchaea

*Strain*	*Isolated from*	*Isolated on*	*Maximum HS*^-^ *production, mM*	*e-Donors (with sulfur)*	*e-Acceptors (with formate)*
HSR6	Kulunda Steppe	Formate+S_8_	14	Formate	S_8_, DMSO
HSR8	Lake Elton		11		S_8_, S_2_O_3_^2−^, DMSO
HSR9	Lake Baskunchak		10		S_8_, S_2_O_3_^2−^, DMSO
HSR15	Eupatoria		14		S_8_, DMSO
Medee-SA6	Lake Medee		16		S_8_, DMSO
Bari-SA6	Solar saltern Bari		12		S_8_, DMSO
HTSR1	Kulunda Steppe	Formate+S_2_O_3_^2−^	10	Formate, H_2_	S_8_, S_2_O_3_^2−^, DMSO
HSR14	Kulunda Steppe	H_2_+S_8_	22	Formate, H_2_	S_8_, DMSO
