# Supplementary material for: Discovery of anaerobic lithoheterotrophic haloarchaea, ubiquitous in hypersaline habitats
Source: ISME J. 2017 Jan 20;11(5):1245–60. doi: 10.1038/ismej.2016.203 (PMC5437934; doi:10.1038/ismej.2016.203)
Supplement: Supplementary Information [file ismej2016203x1.doc]

**SUPPLEMENTARY INFORMATION**

**Sorokin et al.**

**Discovery of first anaerobic lithoheterotrophic haloarchaea, ubiquitous in hypersaline habitats.**

**Contents Page**

**Supplementary Figures S1 to S5 2-6**

**Supplementary Tables S1 to S10 7-13**

**Supplementary Discussion 14**

**Supplementary Methods 15-20**

**References 21-22**

**SUPPLEMENTARY FIGURES**


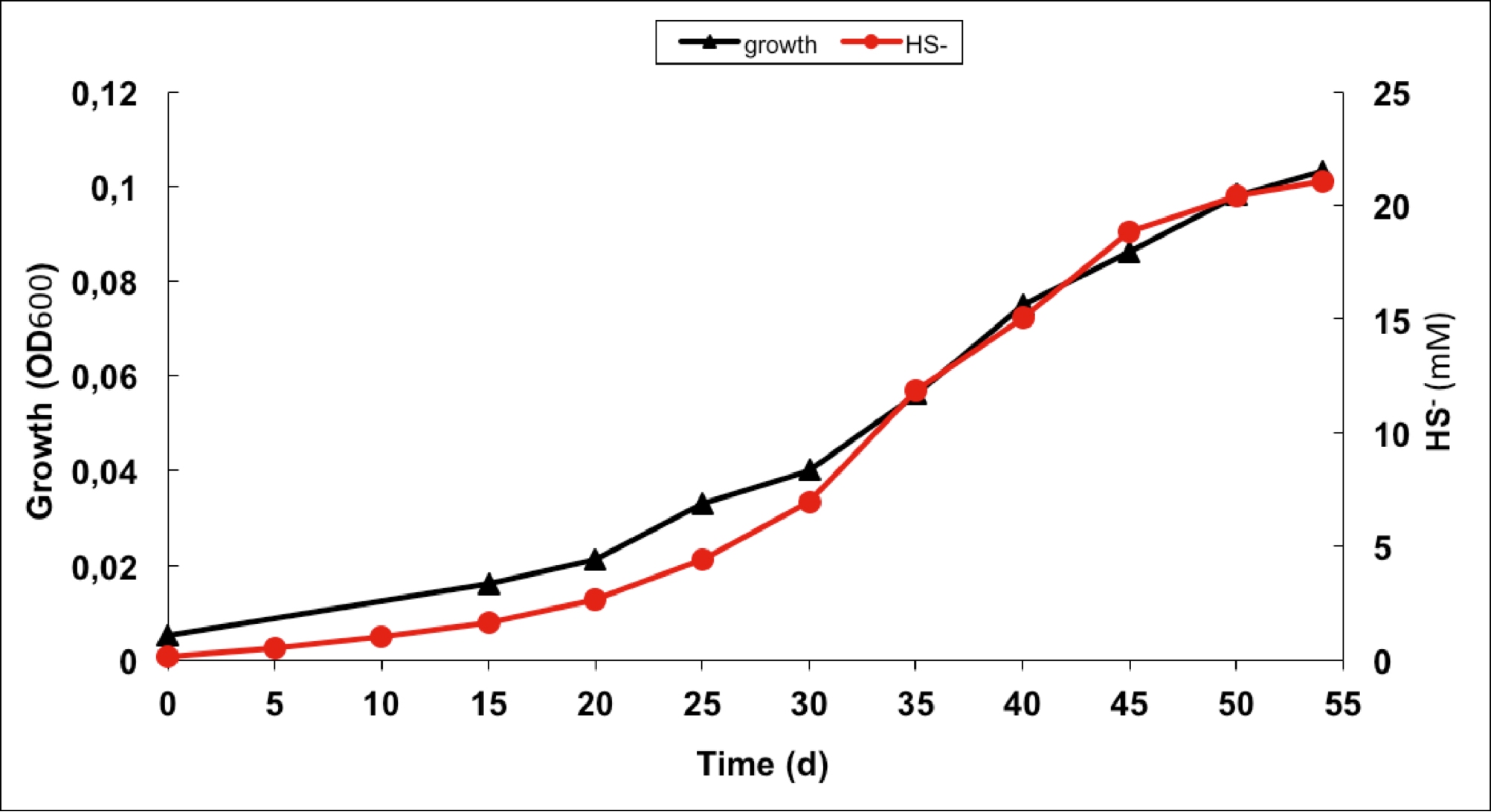


**Figure S1** Sulfidogenic growth kinetics with hydrogen as electron donor and elemental sulfur as electron acceptor of strain HSR14 in 4 M NaCl, pH 7.0 and 37^o^C.


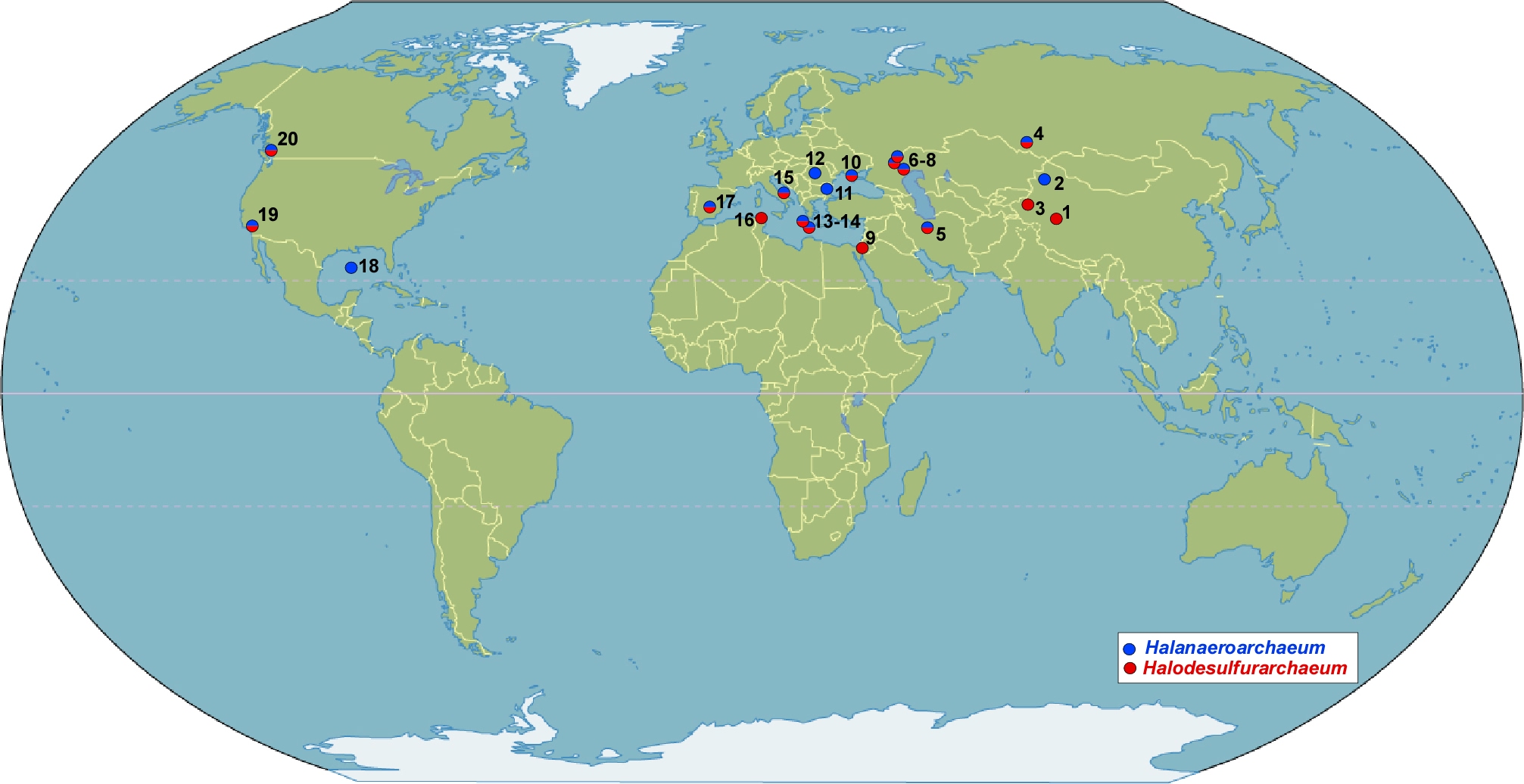


**Figure S2** World map showing the locations of hypersaline habitats where *Halodesulfurarchaeum*- (red circles) *Halanaeroarchaeum*-related (blue circles) organisms were either isolated or detected by molecular phylogenetic techniques: 1. Lake Chaka, Tibet (China) 3200masl (36°41N and 99°09E; Jiang *et al*., 2007); 2. Lake Ebinur, China; (44°53N and 83°00E; KT165165); 3. Lake Xiao’erkule, China; 1555masl (40°06N and 77°16E; JN714413); 4. hypersaline lakes of Kulunda Steppe, Altai, Russia (51°42-56N and 79°46E; Sorokin *et al*. 2016a and present study); 5. Lake Aran-o-Bidgol, Iran (34°63N and 51°90E; Makhdoumi-Kakhi *et al*., 2012); 6. Lake Kasin, Russia (47°36N and 47°27E; Emmerich *et al*., 2012); 7. Lake Elton, Russia (49°09N and 46°40E; Sorokin *et al*. 2016a and present study); 8. Lake Baskunchak, South Russia (48°11N and 46°53E; Sorokin *et al*. 2016a and present study); 9. solar saltern of Eilat, Israele (29°33N and 34°58E, Sørensen *et al*., 2005); 10. solar saltern of Eupatoria, Crimea, Russia (45°11N and 33°27E; present study); 11. solar saltern of Pomorie, Bulgaria (42°34N and 27°37E; LN865045); 12. meromictic Lake Ocnei, Romania; (46°35N and 23°47E; Baricz *et al*., 2014); 13. deep-sea hypersaline Lake Medee, Eastern Mediterranean Sea, 3000 mbsl (34°20N and 22°33E; Sorokin *et al*., 2016a and present study); 14. deep-sea hypersaline Lake Urania, Eastern Mediterranean Sea, 3500 mbsl (35°14N and 21°29E; KM278678); 15. solar saltern of Bari (Italy) (41°23N and 16°06E; Sorokin *et al*., 2016a and present study); 16. Solar saltern (Tunisia) (34°43N and 10°45E; Baati *et al*., 2010); 17. Lake Tirez, Spain (39°32N and 03°21E; Montoya *et al*., 2013); 18. deep-sea hypersaline lake Orca Basin, Gulf of Mexico, 2000mbsl (27°00N and 91°28W); 19. Davis-Schrimpf seep field, Salton Sea, California, USA (33°20N and 115°59W; KC465610); 20. Salt Spring, British Columbia, Canada (48°51N and 123°30W; Walsh *et al*., 2005).


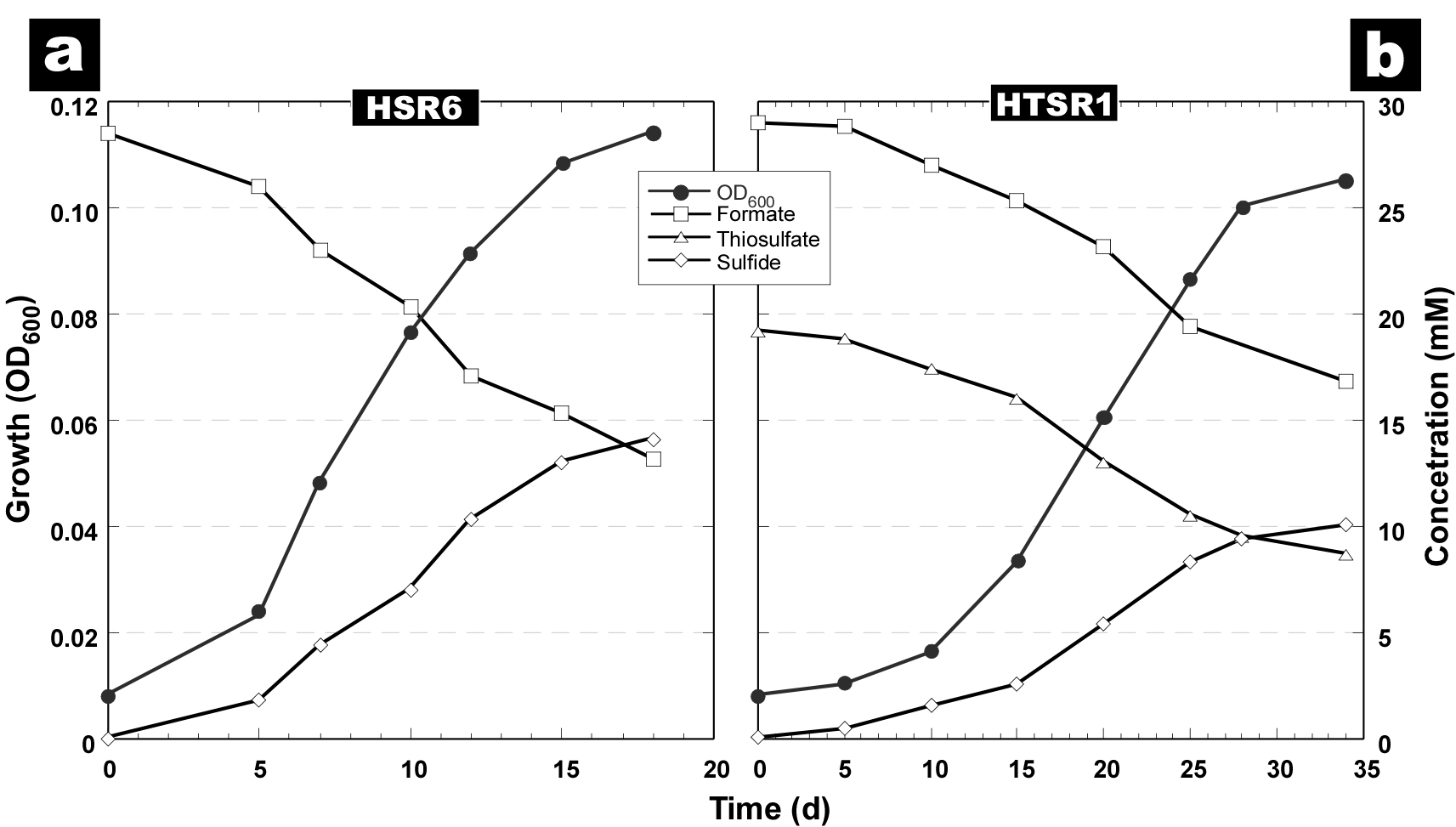


**Figure S3** Sulfidogenic growth kinetics of HSR6 and HTSR1 strains in 4 M NaCl, pH 7.0 and 37^o^C: (**a**) growth of HSR6 with formate as electron donor and elemental sulfur as electron acceptor; (**b**) growth of HTSR1 with formate as electron donor and thiosulfate as electron acceptor. The data are mean values from 3 replicate cultures, the standard deviations were between 2 and 12%.

**Figure S4.** Blast dot-plot (a) of HSR6^T^ genome *vs* HTSR1 genome, obtained by direct protein sequences comparison using the RAST Prokaryotic Genome Annotation Server with default parameters.

Unique genomic regions (b) identified in both *Halodesulfurarchaeum formicicum* genomes by using IslandViewer 3, a computational tool that integrates three different genomic island prediction methods: IslandPick, IslandPath-DIMOB, and SIGI-HMM.

Structures of unique genomic regions (c) in HTSR1 (A) or in HSR6 genomes (B-D). Islands A, B and C started with phage integrase (evidenced as red arrows); island D contains a CRISPR-Cas system, whereas island E (not shown) contains a 51kbp long region that includes 40 proteins present in HSR6, mainly hypothetical proteins and only 12 complements in HTSR1, with <50% of similarity (see Supplementary Table S9 for further details).


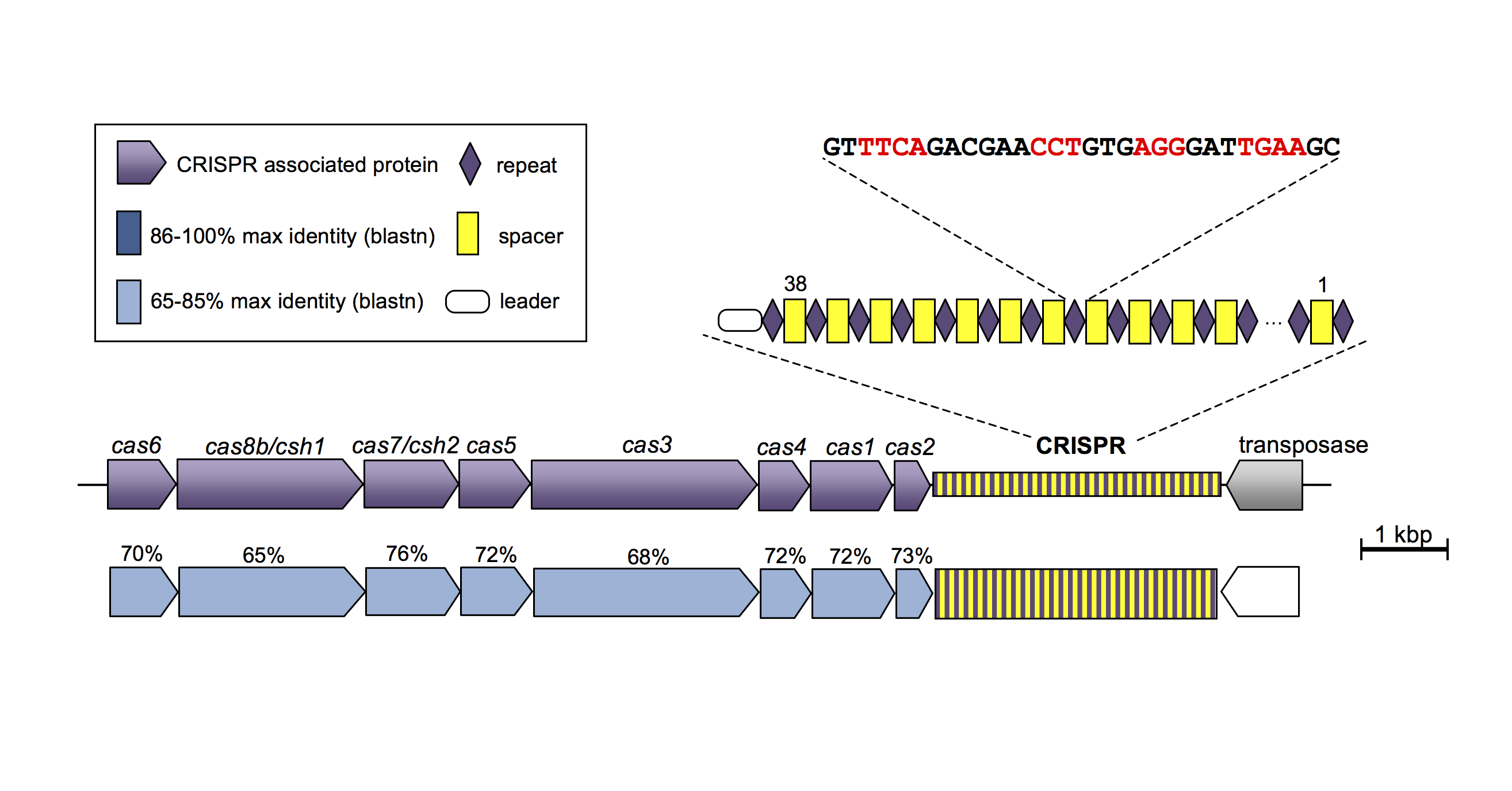


**Figure S5** Structure of CRISPR/Cas system identified inside the genome of *Halodesulfurarchaeum formicicum* HSR6^T^. Percentages of identity were referred to NCBI blastn in nt/nr database (see Supplementary Table S8 for details).

**SUPPLEMENTARY TABLES**

**Table S1** Ecological significance of *Haloanaeroarchaeum* (HAA) and *Halodesulfurarchaeum* (HDA) organisms detected by molecular phylogenetic techniques in variety of hypersaline ecosystems worldwide given as percentage of the total number of analysed clones

| *Site* | *No. of clones* | *Phylogenetic Group^a^* | |
| --- | --- | --- | --- |
|  |  | HAA | HDA |
|  |  |  |  |
| Lake Chaka (3200masl), China; 36°41N99°09E^b^ | 120 | 0 | 5.8 |
| Lake Aran-o-Bidgol, Iran; 34°63N51°90E^c^ | 101 | 3 | 2 |
| Lake Kasin, Russia; 47°36N47°27E^d^ | 231 | 9.1 | 4.3 |
| Lake Elton, Russia; 49°09N46°40E^e^ | 96 | 1 | 3.1 |
| Lake Baskunchak, Russia; 48°11N46°53E^e^ | 96 | 2 | 3.1 |
| Eilat saltern, Israel; 29°33N34°58E^f^ | 54 | 0 | 2 |
| Lake Ocnei, Romania; 46°35N23°47E^g^ | ≈380 | 12-18 | 0 |
| Lake Tirez, Spain; 39°32N03°21E^h^ | 34 | 20.6 | 3 |
| Salt Spring, Canada; 48°51N123°30W; 115cm^i^ | 130 | 14.6 | 0 |
| Salt Spring, Canada; 48°51N123°30W; 300cm^i^ | 114 | 2.6 | 7 |
|  |  |  |  |

^a^Accessory of environmental clones to HAA and HDA phylogenetic groups was performed at similarity level ≥96%

^b^ Jiang *et al*., 2007.

^c^ Makhdoumi-Kakhki *et al*., 2012.

^d^ Emmerich *et al*., 2012.

^e^ our unpublished data.

^f^ Sørensen *et al*., 2005.

^g^ Baricz *et al*., 2014.

^h^ Montoya *et al*., 2013.

^i^ Walsh *et al*., 2005

**Table S2** Induction of sulfidogenic activity (VHS^-^) under different growth conditions in strains HSR6 and HTSR1. Washed cells were incubated at 37°C, 4M NaCl. Cell protein was 0.10-012 mg ml^-1^, incubation time from 48 to 96 h.

| **Substrates** | **VHS^-^ nmol (mg protein h)^-1^** | | | | | |
| --- | --- | --- | --- | --- | --- | --- |
|  | **HSR6. Growth with** | | **HTSR1. Growth with** | | | |
|  | **formate+ S^0^** | **formate+DMSO** |  | **H_2_+S^0^** | **formate+ S_2_O_3_^2-^** | **formate+DMSO** |
|  |  |  |  |  |  |  |
| **formate+S^0^** | 456 | 156 |  | 366 | 115 | 138 |
| **H_2_ + S^0^** | 0 | 0 |  | 341 | 0 | ND |
| **formate+S_2_O_3_^2-^** | 0 | 0 |  | 0 | 102 | 0 |
| **H_2_ + S_2_O_3_^2-^** | ND | ND |  | 0 | 0 | ND |
| **formate+DMSO** | ND | 57 |  | ND | 0 | 32 |
|  |  |  |  |  |  |  |

ND, not determined

**Table S3** Classification and general features of *Halodesulfurarchaeum formicicum* HSR6^T^ according to recommendations on the minimum information about a genome sequence (MIGS) specification

| *MIGS ID* | *Characteristic* | *Details* | *Evidence code* |
| --- | --- | --- | --- |
|  |  |  |  |
|  | Current classification | Domain *Archaea*  Phylum *Euryarchaeota*  Class *Halobacteria*  Order *Halobacteriales*  Family *Halobacteriaceae*  Genus *Halodesulfurarchaeum*  Species *Halodesulfurarchaeum formicicum*  Type strain or HSR6 | TAS  TAS  TAS  TAS  TAS  TAS  TAS  TAS |
|  | Cell shape | Pleomorphic | TAS |
|  | Motility | Motile | TAS |
|  | Sporulation | Non-sporulating | NAS |
|  | Temperature range | 15-50°C | TAS |
|  | Optimum temperature | 40°C | TAS |
|  | Salinity | 3.0-5.0 M NaCl | TAS |
|  | pH range; Optimum | 6.0-8.0; 7.2 | TAS |
| MIGS-22 | Oxygen requirement  Carbon source  Energy source | Strictly anaerobic  Yeast Extract  Formate/H_2_ + Elemental sulfur/thiosulfate | TAS  TAS  TAS |
| MIGS-6 | Habitat | Anoxic hypersaline sediments | TAS |
| MIGS-15 | Biotic relationship | Free-living | TAS |
| MIGS-4 | Geographic location | Hummocky Lake, Kulunda Steppe (Altai, Russia) | TAS |
| MIGS-5 | Sample collection time | July 2011 | TAS |
| MIGS-4.1  MIGS-4.2 | Latitude  Longitude | 51°42’N  79°46’E | TAS  TAS |
| MIGS-4.4 | Altitude | 153 m | TAS |
|  |  |  |  |

Evidence codes –TAS: Traceable Author Statement (i.e., a direct report exists in the literature).

**Table S4** Genome sequencing project information for *Halodesulfurarchaeum formicicum* HSR6^T^ and HTSR1

| *MIGS ID* | *Characteristic* | *HTSR1* | *HSR6* |
| --- | --- | --- | --- |
|  |  |  |  |
| MIGS-28 | Libraries used | Illumina standard library, Miseq Reagent kit v2 | Illumina standard library, Miseq Reagent kit v2 |
| MIGS-29 | Sequencing platform | Illumina MiSeq System | Illumina MiSeq System |
| MIGS-31.2 | Sequencing coverage | 282x | 1284x |
| MIGS-31 | Finishing quality | Finished | Finished |
| MIGS-30 | Assembler | Velvet 1.2.10, Geneious 7.1 | ALLPATHs-LG, Spades 3.7.0, Geneious 7.1 |
| MIGS-32 | Gene calling method  GenBank ID  GenBank date of release  NCBI project ID | Geneious 7.1, Glimmer 3.02, tRNAScan-SE  CP016070  Released  PRJNA323694 | Geneious 7.1, Glimmer 3.02, tRNAScan-SE  CP016804  Released  PRJNA328078 |
| MIGS-13 | Source material identifier | Isolated from anoxic sediments at 5-10 cm from surface of Hummocky Lake, Kulunda Steppe (Altai, Russia). Salinity: 330 g/l; pH: 8.2. Coordinates 51°42'00"N 79°46'00"E. | Isolated from anoxic sediments at 5-10 cm from surface of Hummocky Lake, Kulunda Steppe (Altai, Russia). Salinity: 330 g/l; pH: 8.2. Coordinates 51°42'00"N 79°46'00"E. |
|  | Project relevance | Extremophile hypersaline environments | Extremophile hypersaline environments |
|  |  |  |  |

TBD, to be detrmined

**Table S5** Genome statistics for *Halodesulfurarchaeum formicicum* HTSR1 and HSR6^T^

| *Attribute* | *HTSR1* | *% of Total* | *HSR6* | *% of Total* |
| --- | --- | --- | --- | --- |
|  |  |  |  |  |
| Chromosome size (bp) | 1,972,283 |  | 2,085,946 |  |
| DNA coding region (bp) | 1,789,176 | 90.72% | 1,878,279 | 90.04% |
| DNA G+C content (bp) | 1,257,600 | 63.76% | 1,327,103 | 63.62% |
| Total genes | 2,071 |  | 2,148 |  |
| tRNA genes | 45 | 2.17% | 45 | 2.09% |
| rRNA genes (5S-16S-23S) | 3 | 0.14% | 3 | 0.14% |
| Protein-coding genes | 2,023 | 97.68% | 2,100 | 97.77% |
| Genes assigned to COGs | 1,310 | 64.76% | 1,318 | 62.76% |
| Average length (bp) | 884 |  | 894 |  |
| Max length (bp) | 4,941 |  | 7,731 |  |
| ATG initiation codon proteins | 1,617 | 79.93% | 1,749 | 83.29% |
| GTG initiation codon proteins | 343 | 16.96% | 301 | 14.33% |
| TTG initiation codon proteins | 63 | 3.11% | 50 | 2.38% |
| CRISPR repeats | - |  | 1 |  |
|  |  |  |  |  |

**Table S6** Number of genes associated with the general COG functional categories for *Halodesulfurarchaeum formicicum* HTSR1 and HSR6

| *Code* | *HTSR1* | *%age* | *HSR6* | *%age* | *COG category* |
| --- | --- | --- | --- | --- | --- |
|  |  |  |  |  |  |
| J | 130 | 6.43% | 129 | 6.14% | Translation, ribosomal structure and biogenesis |
| A | 1 | 0.05% | 1 | 0.05% | RNA processing and modification |
| K | 68 | 3.36% | 65 | 3.10% | Transcription |
| L | 71 | 3.51% | 79 | 3.76% | Replication, recombination and repair |
| B | 2 | 0.10% | 2 | 0.10% | Chromatin structure and dynamics |
| D | 9 | 0.44% | 9 | 0.43% | Cell cycle control, cell division, chromosome partitioning |
| V | 9 | 0.44% | 9 | 0.43% | Defence mechanisms |
| T | 32 | 1.58% | 32 | 1.52% | Signal transduction mechanisms |
| M | 52 | 2.57% | 53 | 2.52% | Cell wall/membrane/envelope biogenesis |
| N | 26 | 1.29% | 26 | 1.24% | Cell motility |
| U | 8 | 0.40% | 8 | 0.38% | Intracellular trafficking, secretion, and vesicular transport |
| O | 61 | 3.02% | 60 | 2.86% | Posttranslational modification, protein turnover, chaperones |
| C | 114 | 5.64% | 113 | 5.38% | Energy production and conversion |
| G | 25 | 1.23% | 25 | 1.19% | Carbohydrate transport and metabolism |
| E | 129 | 6.38% | 127 | 6.05% | Amino acid transport and metabolism |
| F | 55 | 2.72% | 57 | 2.71% | Nucleotide transport and metabolism |
| H | 97 | 4.79% | 95 | 4.52% | Coenzyme transport and metabolism |
| I | 19 | 0.94% | 18 | 0.86% | Lipid transport and metabolism |
| P | 72 | 3.56% | 76 | 3.62% | Inorganic ion transport and metabolism |
| Q | 2 | 0.10% | 3 | 0.14% | Secondary metabolites biosynthesis, transport and catabolism |
| R | 192 | 9.49% | 195 | 9.29% | General function prediction only |
| S | 136 | 6.72% | 136 | 6.48% | Function unknown |
| - | 713 | 35.24% | 782 | 37.24% | Not in COGs |
|  |  |  |  |  |  |

**Table S7** NCBI blastx results for CRISPR associated proteins in *Halodesulfurarchaeum formicicum* HSR6^T^

| *locus_tag* | *Gene* | *Blastx best hit (nr)* | *Max score* | *Identity* | *E value* | *Accession* |
| --- | --- | --- | --- | --- | --- | --- |
|  |  |  |  |  |  |  |
| HSR6_1252 | Cas6 | *Halobiforma lacisalsi* AJ5 | 355 | 84% | 3e-122 | EMA30171.1 |
| HSR6_1253 | Cas8b/Csh1 | *Haloterrigena* sp. H13 | 918 | 66% | 0 | OAQ51094.1 |
| HSR6_1254 | Cas7/Csh2 | *Halobiforma lacisalsi* AJ5 | 589 | 87% | 0 | EMA30169.1 |
| HSR6_1255 | Cas5 | *Haladaptatus* sp. R4 | 447 | 80% | 9e-158 | KZN26058.1 |
| HSR6_1256 | Cas3 | *Haloterrigena* sp. H13 | 1239 | 69% | 0 | OAQ51091.1 |
| HSR6_1257 | Cas4 | *Haloterrigena* sp. H13 | 255 | 83% | 6e-85 | WP_066304144.1 |
| HSR6_1258 | Cas1 | *Haloterrigena* sp. H13 | 597 | 84% | 0 | OAQ51090.1 |
| HSR6_1259 | Cas2 | *Haloterrigena* sp. H13 | 151 | 80% | 2e-46 | WP_066304139.1 |
|  |  |  |  |  |  |  |

**Table S10** The CISM enzymatic complexes locations found in HTSR1 and HSR6 genomes.

| *#CISM* | *Name* | *HTSR1 locus_tag* | *HSR6 locus_tag* |
| --- | --- | --- | --- |
|  |  |  |  |
| 1 | DMSOR | HTSR_0420-HTSR_0427 | HSR6_0405-HSR6_0412 |
| 2 | DMSOR | HTSR_0521-HTSR_0514 | HSR6_0505-HSR6_0498 |
| **3** | **Deep Unaffiliated** | **HTSR_0625-HTSR_0630** | - |
| 4 | PSR | HTSR_1351-HTSR_1343 | HSR6_1423-HSR6_1415 |
| 5 | TSR | HTSR_1524-HTSR_1521 | HSR6_1595-HSR6_1592 |
| 6 | FDH | HTSR_1577-HTSR_1571 | HSR6_1646-HSR6_1640 |
| 7 | PSR | HTSR_1664-HTSR_1658 | HSR6_1732-HSR6_1726 |
| 8 | PSR | HTSR_1701-HTSR_1697 | HSR6_1770-HSR6_1766 |
| 9-10 | FDH | HTSR_1736-HTSR_1741 | HSR6_1802-HSR6_1807 |
|  |  |  |  |

Sequentially numbered CISM enzymatic complexes (as in Figure 3 and Figure 7), including two DMSO reductases DMSOR (1, 2), one unaffiliated CISM complex ‘Deep’ (3) highlighted in bold, three polysulfide reductases PSR (4, 7, 8), one thiosulfate reductase TSR (5) and three formate dehydrogenases FDH (6, 9-10).

**SUPPLEMENTARY DISCUSSION**

*Differential proteome analysis*

To validate the proposed metabolic pathways during respiration with different terminal acceptors, the global protein expression was assayed by 1D-nano LC ESI-MSMS shotgun proteomic analysis. Protein extracts were obtained from the strain HTSR1 cultures grown on either elemental sulfur, thiosulfate or DMSO as the terminal electron acceptors with formate as the electron donor. The analysis had confidentially verified the quantitative expression of 1,638 proteins, representing ~79% of the theoretical proteome (2,074 protein-coding genes), which is far above the values typically obtained in quantitative proteomic analyses of haloarchaea (Klein *et al*., 2005). A detailed description of the peptide*-*level scoring metrics is provided in Supplementary Table S10. Although we did not aim here to perform the detailed comparative analysis of HTSR1 proteomes, it must be specified that more than two-thirds of all detected proteins, including those identified by a single peptide fragment, were expressed in any conditions (Figure 9A). Among very few proteins, solely found in one growth experiment, only one enzymatic complex (thiosulfate reductase, HTSR_1522-1524) was involved in the energy and carbon metabolism. Inspection of the proteome revealed that the proposed C_1_ metabolism in HDA is active, since eleven enzymes of the pathways depicted on Figure 8 were among the most abundant proteins.

**SUPPLEMENTARY METHODS**

*Lipid analyses*

The core membrane lipids were extracted from freeze-dried cells using dichloromethane (DCM): methanol MeOH (9 : 1, vol / vol). The extracts were further purified after concentration by separation into a apolar and relatively polar fraction over an activated Al_2_O_3_ column using DCM : MeOH (199 : 1, vol / vol) and DCM : MeOH (1 : 1, vol / vol), respectively. After ultrasonically driven dissolution in hexane, the polar fraction, containing the archaeol, MGE and DGE, was analysed using HPLC/atmospheric pressure chemical ionisation – mass spectrometry (HPLC/APCI-MS) with Agilent 1100/Hewlett Packard 1100 MSD instrument equipped with automatic injector and HP-Chemstation software. Separation was achieved in normal phase with a Prevail Cyano column (150mm × 2.1 mm; 3 µm) with a flow rate of the hexane:propanol (99:1, v/v) eluent of 0.2 ml min−1, isocratically for the first 5 min and thereafter with a linear gradient to 1.8% propanol after 45 min. Injection volume was 10 µl. The intact polar lipids were extracted from the lyophilized cells following the procedure described by Pitcher *et al*. (2009). An aliquot of the extract obtained was dissolved in hexane:2-propanol:water (72:27:1), filtered through a 0.45-μm regenerated cellulose filter, and analyzed by HPLC-electrospray ionization (ESI) MS using the Agilent 1200 series liquid chromatograph equipped with a thermostat-controlled autoinjector and a column oven and coupled to a Thermo LTQ XL linear ion trap with an Ion Max source with an ESI probe (Thermo Scientific, Waltham, MA). The separation was achieved with a Lichrosphere diol column (250 mm by 2.1mm, 5 µm particles; Alltech Associates Inc.) at 30^o^C.

*Sequencing, assembly and annotation of genomes of strains HSR6 and HTSR1*

The HTSR1 genome was sequenced with MiSeq^TM^ Personal Sequencing System technology of Illumina Inc. (San Diego, CA, USA) using paired-end 250-bp reads. The library was prepared from 1 µg of genomic DNA with NEBNext Ultra DNA library preparation kit (NewEngland Biolabs, Ipswich, USA) according to manufacturer’s instructions. Sequencing run resulted in 2,596,978 paired-end reads with an average read length of 250 bp, yielding 649.2 Mbp. These reads were assembled using both Velvet 1.2.10 (Zerbino and Birney, 2008) and Geneious 7.1 software (Biomatters Ltd, New Zealand). Gaps between contigs were closed with a conventional PCR-based gap closure approach and supported by manual refining with Geneious 7.1 embedded tools, resulting in a fully closed circular chromosome of 1,972,283 bp with 282x of sequence coverage. For sequencing of HSR6 genome, both paired-end and mate-paired DNA libraries were used. Paired end library was prepared from 1 µg of genomic DNA with NEBNext Ultra DNA library preparation kit (New England Biolabs, Ipswich, USA) according to manufacturer’s instructions to obtain mean library size of 500 bp. Mate-paired libraries were prepared with Nextera™ Mate Pair Library Prep Kit (Illumina Inc., San Diego, CA, USA). During procedure after initial DNA fragmentation step gel-based size selection were used to obtain libraries with insert size ranges of 2-3, 4-6 and 8-10 kb. Finally, one paired-end and three mate-paired libraries were sequenced with 2x250 bp reads with MiSeq™ Personal Sequencing System (Illumina Inc., San Diego, CA, USA). After sequencing all reads were subjected to stringent quality filtering with CLC Genomics Workbench 8.5 (Qiagen, Germany). After filtering, overlapping paired-end library reads were merged with SeqPrep tool (<https://github.com/jstjohn/SeqPrep>) resulting in 881,557 single reads and 696388 read pairs. Mate paired reads were treated with NextClip tool (Leggett *et al*., 2014), resulting in 2,379,876, 1,821,129 and 1,352,861 read pairs with mean insert size of 2670, 5019 and 9745 bp, respectively. Reads were assembled with both ALLPATHS-LG (Butler *et al*., 2008) and SPADES 3.7.0 (Nurk *et al*., 2013) assemblers and refined by Geneious 7.1 software (Biomatters Ltd, New Zealand), resulting in a fully closed circular chromosome of 2,085,946 bp with 1284x of sequence coverage.

*Proteomic analyses*

Cell pellets were dissolved in lysis buffer (8M urea, 2M thiourea, 5% (w/w) CHAPS, 2mM TCEP-HCl and protease inhibitor). Homogenization of the cells was achieved by ultrasonication on ice (10 strokes, low amplitude). After homogenization, the lysed cells were centrifuged at 20000 × g for 10 min at 4 °C and the supernatant containing the solubilized proteins was used for LC-MS/MS experiment. Total protein concentration was determined using Pierce 660nm protein assay (Thermo). An aliquot or every sample was diluted with loading sample buffer and applied onto 1.2-cm wide wells of a conventional sodium dodecyl sulfate polyacrylamide gel electrophoresis SDS-PAGE gel (1mm-thick, 4% stacking, and 12% resolving). Then run was stopped as soon as the front entered 1 cm into the resolving gel, so that the whole proteome became concentrated in the stacking/resolving gel interface. The unseparated protein bands were visualized by Coomassie staining, excised, cut into cubes (1 mm^2^), deposited in 96-well plates and processed automatically in a Proteineer DP (Bruker Daltonics, Bremen, Germany). The digestion protocol used was based on Schevchenko *et al*. (1996) with minor variations: gel plugs were washed firstly with 50 mM ammonium bicarbonate and secondly with acetonitrile (ACN) prior to reduction with 10 mM dithiothreitol (DTT) in 25 mM ammonium bicarbonate solution, and alkylation was carried out with 55 mM iodoacetamide (IAA) in 50 mM ammonium bicarbonate solution. Gel pieces were then rinsed firstly with 50 mM ammonium bicarbonate and secondly with ACN, and then were dried under a stream of nitrogen. Proteomics Grade Trypsin (Sigma Aldrich) at a final concentration of 16 ng/μl in 25% ACN/50 mM ammonium bicarbonate solution was added and the digestion took place at 37°C for 4h. The reaction was stopped by adding 50% ACN / 0.5% trifluoracetic acid (TFA) for peptide extraction. The tryptic eluted peptides were dried by speed-vacuum centrifugation and then desalted onto StageTip C18 Pipette tips (Thermo Scientific) until the mass spectrometric analysis.

A 1 µg aliquot of each sample was subjected to 1D-nano liquid chromatography-electrospray ionization-tandem mass spectrometry (LC ESI-MSMS) analysis using a nano liquid chromatography system (Eksigent Technologies nanoLC Ultra 1D plus, AB SCIEX, Foster City, CA) coupled to high speed Triple quadrupole time-of-flight (TOF) 5600 mass spectrometer (AB SCIEX , Foster City, CA) with a Nanospray III source. The analytical column used was a silica-based reversed phase Acquity UPLC® M-Class Peptide BEH C18 Column, 75 µm × 150 mm, 1.7 µm particle size and 130 Å pore size (Waters). The trap column was a C18 Acclaim PepMap^TM^ 100 (Thermo Scientific), 100 µm × 2 cm, 5 µm particle diameter, 100 Å pore size, switched on-line with the analytical column. The loading pump delivered a solution of 0.1% formic acid in water at 2 µl/min. The nano-pump provided a flow-rate of 250 nl/min and was operated under gradient elution conditions. Peptides were separated using a 250 minutes gradient ranging from 2% to 90% mobile phase B (mobile phase A: 2% acetonitrile, 0.1% formic acid; mobile phase B: 100% acetonitrile, 0.1% formic acid). Injection volume was 5 µl.

Data acquisition was performed with a TripleTOF 5600 System (AB SCIEX, Concord, ON). Data was acquired using an ionspray voltage floating (ISVF) 2300 V, curtain gas (CUR) 35, interface heater temperature (IHT) 150, ion source gas 1 (GS1) 25, declustering potential (DP) 100 V. All data was acquired using information-dependent acquisition (IDA) mode with Analyst TF 1.7 software (SCIEX, USA). For IDA parameters, 0.25s MS survey scan in the mass range of 350–1250 Da were followed by 35 MS/MS scans of 100ms in the mass range of 100–1800 (total cycle time: 4 s). Switching criteria were set to ions greater than mass to charge ratio (m/z) 350 and smaller than m/z 1250 with charge state of 2–5 and an abundance threshold of more than 90 counts (cps). Former target ions were excluded for 15s. IDA rolling collision energy (CE) parameters script was used for automatically controlling the CE.

MS and MS/MS data obtained for individual samples were acquired with a TripleTOF 5600 System (AB SCIEX, Concord, ON) and processed using information-dependent acquisition (IDA) mode with Analyst TF 1.7 software (SCIEX, USA). The reconstituted HTSR1 chromosome sequence was used to generate the database for protein identification using the Mascot Server v. 2.5.1 (Matrix Science, London, UK). Search parameters were set as follows: carbamidomethyl cysteine as fixed modification and acetyl (Protein N-term), Gln to pyro-Glu (N-term Q), Glu to pyro-Glu (N-term E) and methionine oxidation as variable modifications. Peptide mass tolerance was set to 25 ppm and 0.05 Da for fragment masses, also 2 missed cleavages were allowed. False Discovery Rates (FDR ≤ 1% at PSM level) for peptide identification were manually calculated. The threshold of only one identified peptide per protein identification was used because FDR controlled experiments counter intuitively suffer from the two‐peptide rule (Gupta and Pevzner, 2009). The number of peptides observed per protein provides an estimate of the protein's abundance (Lu *et al*., 2007). Therefore, this value was used in the present study to estimate the relative abundance of proteins: (N_protein_ / N_total_) x 100%, where N_protein_ corresponds to a number of peptides, attributed to a single protein, and N_total_ is the total number of peptides analysed in each proteome. The normalized protein content value was calculated as a percentage of each exponentially modified protein abundance index in the summation of all identified proteins. The circular HTSR1 proteomic map was built based on information present in Supplementary Table S10.

**Supplementary References**

Baati H, Guermazi S, Gharsallah N, Sghir A, Ammar E. (2010). Novel prokaryotic diversity in sediments of Tunisian multipond solar saltern. *Res Microbiol* **161**:573-582.

Baricz A, Coman C, Andrei AŞ, Muntean V, Keresztes ZG, Păuşan M, Alexe M, Banciu HL. (2014). Spatial and temporal distribution of archaeal diversity in meromictic, hypersaline Ocnei Lake (Transylvanian Basin, Romania). *Extremophiles* **18**:399-413.

Emmerich M, Bhansali A, Lösekann-Behrens T, Schröder C, Kappler A, Behrens S. (2012). Abundance, distribution, and activity of Fe (II)-oxidizing and Fe (III)-reducing microorganisms in hypersaline sediments of Lake Kasin, southern Russia. *Appl Environ Microbiol* **78**: 4386-4399.

Jiang H, Dong H, Yu B, Liu X, Li Y, Ji S, Zhang CL. (2007) Microbial response to salinity change in Lake Chaka, a hypersaline lake on Tibetan plateau. Environmental Microbiology. 2007 Oct 1;9(10):2603-21.

Khomyakova M, Bükmez Ö, Thomas LK, Erb TJ, Berg IA. (2011). A methylaspartate cycle in haloarchaea. *Science* **331**: 334–337.

Klein C, Garcia-Rizo C, Bisle B, Scheffer B, Zischka H, Pfeiffer F *et al.* (2005). The membrane proteome of *Halobacterium salinarum*. *Proteomics* **5**: 180–197.

Leggett RM, Clavijo BJ, Clissold L, Clark MD, Caccamo M. (2014). NextClip: an analysis and read preparation tool for Nextera Long Mate Pair libraries. *Bioinformatics* **30**: 566-568.

Makhdoumi-Kakhki A, Amoozegar MA, Kazemi B, PaiC L, Ventosa A. (2012) Prokaryotic diversity in Aran-Bidgol salt lake, the largest hypersaline playa in Iran. *Microbes and Environment*s **27**: 87-93.

Montoya L, Vizioli C, Rodríguez N, Rastoll MJ, Amils R, Marin I. (2013) Microbial community composition of Tirez lagoon (Spain), a highly sulfated athalassohaline environment. *Aquatic biosystems* **9**: 19.

Oren A. (2011). Thermodynamic limits to microbial life at high salt concentrations. *Environ Microbiol* **13**: 1908–1923.

Pitcher A, Hopmans EC, Schouten S, Sinninghe Damste´ JS. (2009). Separation of core and intact polar archaeal tetraether lipids using silica columns: insights into living and fossil biomass contributions. *Org Geochem* **40:** 12–19.

Say RF, Fuchs G. (2010). Fructose 1,6-bisphosphate aldolase/phosphatase may be an ancestral gluconeogenic enzyme. *Nature* **464**: 1077–1081.

Shevchenko A, Wilm M, Vorm O, Mann M. (1996). Mass spectrometric sequencing of proteins silver-stained polyacrylamide gels. *Anal Chem* **68**: 850-858.

Sørensen KB, Canfield DE, Teske AP, Oren A. (2005). Community composition of a hypersaline endoevaporitic microbial mat. *Appl Environ Microbiol* **71**: 7352-7365.

Walsh DA, Papke RT, Doolittle WF. (2005). Archaeal diversity along a soil salinity gradient prone to disturbance. *Environ Microbiol* **7**: 1655-1666.

Zerbino DR, Birney E. (2008). Velvet: algorithms for de novo short read assembly using de Bruijn graphs. *Genome Res* **18**: 821–829.
