# Supplementary material for: Discovery of anaerobic lithoheterotrophic haloarchaea, ubiquitous in hypersaline habitats
Source: ISME J. 2017 Jan 20;11(5):1245–60. doi: 10.1038/ismej.2016.203 (PMC5437934; doi:10.1038/ismej.2016.203)
Supplement: Supplementary Table S8 [file ismej2016203x2.pdf]

Table S8 Detailed structure of the main unique islands detected in HTSR1 (yellow) and HSR6 (orange) genomes. Genes that match with similarity greater than 90% are highlighted in light green.

Length (AA)	Locus tag	Function	Locus tag	AA similarity %	Function
	**HTSR1 UNIQUE ISLAND (A)**		**HTSR6**		
108	HTSR_0929	hypothetical protein	HSR6_0943	99.1	
334	HTSR_0930	phage integrase	HSR6_0952	40.06	phage integrase
192	HTSR_0931	hypothetical protein		0	
78	HTSR_0932	hypothetical protein		0	
247	HTSR_0933	site-specific recombinase, DNA invertase Pin		0	
1142	HTSR_0934	type II restriction enzyme, methylase subunits		0	
124	HTSR_0935	hypothetical protein		0	
222	HTSR_0936	hypothetical protein		0	
321	HTSR_0937	hypothetical protein		0	
69	HTSR_0938	hypothetical protein		0	
605	HTSR_0939	hypothetical protein		0	
588	HTSR_0940	hypothetical protein		0	
474	HTSR_0941	hypothetical protein		0	
597	HTSR_0942	hypothetical protein		0	
120	HTSR_0943	hypothetical protein		0	
292	HTSR_0944	hypothetical protein	HSR6_0945	97.83	hypothetical protein
	**HTSR6 UNIQUE ISLAND (B)**		**HTSR1**		
987	HSR6_0951	PAS domain s-box	HTSR_0951	99.8	
357	HSR6_0952	phage integrase	HTSR_0930	40.06	phage integrase
101	HSR6_0953	hypothetical protein		0	
58	HSR6_0954	hypothetical protein		0	
78	HSR6_0955	hypothetical protein		0	
82	HSR6_0956	phage repressor protein		0	
88	HSR6_0957	hypothetical protein		0	
483	HSR6_0958	competence protein		0	
178	HSR6_0959	hypothetical protein		0	
	**HTSR6 UNIQUE ISLAND (C)**		**HTSR1**		
316	HSR6_0960	XerC/D-like integrase	HTSR_0961	26.49	integrase/recombinase
149	HSR6_0961	cytidine deaminase	HTSR_0786	34.62	Cytidine deaminase (EC 3.5.4.5)
59	HSR6_0962	hypothetical protein		0	
464	HSR6_0963	site-specific DNA-methyltransferase		0	
248	HSR6_0964	hypothetical protein		0	
686	HSR6_0965	hypothetical protein		0	
493	HSR6_0966	dGTPase (EC 3.1.5.1)		0	
86	HSR6_0967	hypothetical protein		0	
449	HSR6_0968	DNA methylase		0	
236	HSR6_0969	hypothetical protein		0	
583	HSR6_0970	hypothetical protein		0	
126	HSR6_0971	hypothetical protein		0	
146	HSR6_0972	hypothetical protein		0	
546	HSR6_0973	hypothetical protein		0	
272	HSR6_0974	hypothetical protein		0	
453	HSR6_0975	hypothetical protein		0	
81	HSR6_0976	hypothetical protein		0	
1034	HSR6_0977	hypothetical protein		0	
98	HSR6_0978	hypothetical protein		0	
431	HSR6_0979	hypothetical protein		0	
62	HSR6_0980	hypothetical protein		0	
104	HSR6_0981	hypothetical protein		0	
79	HSR6_0982	hypothetical protein		0	
483	HSR6_0984	DHH family phosphoesterase	HTSR_0953	100	
	**HSR6 UNIQUE ISLAND (D)**		**HTSR1**		
525	HSR6_1251	ATP-dependent DNA helicase	HTSR_1724	58.44	ATP-dependent DNA helicase pcrA (EC 3.6.1.-)
156	HSR6_1252	CRISPR-associated protein Cas6		0	
716	HSR6_1253	CRISPR-associated protein Cas8b/Csh1		0	
342	HSR6_1254	CRISPR-associated protein Cas7/Csh2		0	
141	HSR6_1255	CRISPR-associated protein Cas5		0	
707	HSR6_1256	CRISPR-associated helicase Cas3		0	
156	HSR6_1257	CRISPR-associated protein Cas4		0	
332	HSR6_1258	CRISPR-associated protein Cas1		0	
87	HSR6_1259	CRISPR-associated protein Cas2		0	
49	HSR6_1260	transposase		0	
90	HSR6_1261	cell division control protein 6	HTSR_0404	60.61	f replication recognition protein @ Cell division control p
233	HSR6_1262	hypothetical protein		0	
92	HSR6_1263	hypothetical protein		0	
706	HSR6_1264	archaeal DNA polymerase I (EC 2.7.7.7)	HTSR_0400	70.88	archaeal DNA polymerase I (EC 2.7.7.7)
71	HSR6_1265	hypothetical protein		0	
301	HSR6_1266	hypothetical protein		0	
275	HSR6_1267	hypothetical protein		0	
415	HSR6_1268	hypothetical protein		0	
1131	HSR6_1269	UvrD/REP helicase domain-containing protein		0	
429	HSR6_1270	integrase		0	
38	HSR6_1271	hypothetical protein		0	
41	HSR6_1272	integrase		0	
173	HSR6_1273	hypothetical protein	HTSR_1203	90.2	hypothetical protein
	**HSR6 UNIQUE-DISCONTINUOUS ISLAND (E)**		**HTSR1**		
451	HSR6_0605	alpha-L-glutamate ligase, RimK family	HTSR_0617	96.57	Ribosomal protein S6 glutaminyl transferase
-	HSR6_0606	tRNA-Glu	HTSR_0618	100	
92	HSR6_0607	hypothetical protein		0	
86	HSR6_0608	hypothetical protein		0	
175	HSR6_0609	hypothetical protein		0	
101	HSR6_0610	hypothetical protein		0	
66	HSR6_0611	copper chaperone CopZ		0	
687	HSR6_0612	copper-translocating P-type ATPase CopB (EC 3.6.3.4)	HTSR_1157	36.38	nsporting ATPase (EC 3.6.3.3) (EC 3.6.3.5); Copper-tran
91	HSR6_0613	hypothetical protein		0	
604	HSR6_0614	nine--fructose-6-phosphate aminotransferase (isomerizing) (EC	HTSR_0882	51.71	e--fructose-6-phosphate aminotransferase [isomerizing] (
227	HSR6_0615	hypothetical protein	HTSR_0910	41.38	hypothetical protein
185	HSR6_0616	hypothetical protein		0	
72	HSR6_0617	hypothetical protein		0	
92	HSR6_0618	transcriptional regulator		0	
50	HSR6_0619	hypothetical protein		0	
68	HSR6_0620	hypothetical protein		0	
239	HSR6_0621	hypothetical protein		0	
84	HSR6_0622	hypothetical protein	HTSR_0788	32.89	hypothetical protein
564	HSR6_0623	ne pyrophosphorylase / glucosamine-1-phosphate N-acetyltran	HTSR_0890	57.22	idyltransferase (EC 2.7.7.23) / Glucosamine-1-phosphat
447	HSR6_0624	phosphoglucosamine mutase (EC 5.4.2.10)	HTSR_0097	35.03	nomutase (EC 5.4.2.8) / Phosphoglucosamine mutase (
115	HSR6_0625	NUDIX hydrolase		0	

88	HSR6_0626	hypothetical protein		0	
376	HSR6_0627	hypothetical protein		0	
456	HSR6_0628	nucleotide sugar dehydrogenase	HTSR_0878	30.23	UDP-glucose dehydrogenase (EC 1.1.1.22)
269	HSR6_0629	oxidoreductase		0	
323	HSR6_0630	polysaccharide deacetylase		0	
400	HSR6_0631	ATP-grasp protein		0	
399	HSR6_0632	dTDP-4-dehydro-6-deoxyglucose aminotransferase		0	
722	HSR6_0633	hypothetical protein		0	
327	HSR6_0634	hypothetical protein	HTSR_2027	31.49	hypothetical protein
489	HSR6_0635	hypothetical protein		0	
278	HSR6_0636	hypothetical protein		0	
506	HSR6_0637	polysaccharide biosynthesis protein		0	
334	HSR6_0638	glycosyl transferase family 2	HTSR_0233	26.72	Mycofactocin system glycosyltransferase
654	HSR6_0639	hypothetical protein		0	
70	HSR6_0640	SigmaK-factor processing regulatory protein BofA	HTSR_0106	35.59	hypothetical protein
217	HSR6_0641	HAD-superfamily hydrolase, subfamily IA, variant1		0	
373	HSR6_0642	carbamoyl phosphate synthase large subunit		0	
388	HSR6_0643	glycosyl transferase family 2	HTSR_1227	36.16	glycosyl transferase, family 2
257	HSR6_0644	hypothetical protein		0	
164	HSR6_0645	hypothetical protein		0	
367	HSR6_0646	dTDP-4-amino-4,6-dideoxygalactose transaminase		0	
187	HSR6_0647	transferase	HTSR_0711	35.58	acetyltransferase homolog
434	HSR6_0648	arylsulfatase	HTSR_2027	34.25	hypothetical protein
311	HSR6_0649	glycosyl transferase family 1		0	
642	HSR6_0650	hypothetical protein		0	
167	HSR6_0651	hypothetical protein		0	
315	HSR6_0652	UDP-glucose 4-epimerase (EC 5.1.3.2)	HTSR_1766	37.14	hypothetical protein
280	HSR6_0653	hypothetical protein	HTSR_0622	90.71	hypothetical protein
61	HSR6_0654	arsenic resistance protein ArsB	HTSR_0623	76.74	Arsenical-resistance protein ACR3
47	HSR6_0655	ArsR family transcriptional regulator	HTSR_0624	86.84	Arsenical resistance operon repressor
141	HSR6_0656	CoA-binding domain-containing protein	HTSR_0631	100	ulfhydrylase (EC 2.5.1.49) / O-succinylhomoserine sulfhy
382	HSR6_0657	permease	HTSR_0632	99.7	
