# Supplementary material for: Discovery of anaerobic lithoheterotrophic haloarchaea, ubiquitous in hypersaline habitats
Source: ISME J. 2017 Jan 20;11(5):1245–60. doi: 10.1038/ismej.2016.203 (PMC5437934; doi:10.1038/ismej.2016.203)
Supplement: Supplementary Table S9 [file ismej2016203x3.pdf]

Table S9 Enzymes and other proteins predicted from the haloarchaeal genomes involved in the main metabolic pathways to be relevant for their ecophysiologies. AA similarity obtained by direct protein sequences comparison using the RAST Prokaryotic Genome Annotation Server.

**Primary transport**	**gene_name**	**locus HTSR1**	**locus HSR6**	**AA similarity**
Oligopeptide transport ATP-binding protein OppF (TC 3.A.1.5.1)		HTSR_0046	HSR6_0046	98.53
Dipeptide-binding ABC transporter, periplasmic substrate-binding component (TC 3.A.1.5.2)		HTSR_0047	HSR6_0047	98.56
Dipeptide transport system permease protein DppB (TC 3.A.1.5.2)		HTSR_0048	HSR6_0048	99.12
Dipeptide transport system permease protein DppC (TC 3.A.1.5.2)		HTSR_0049	HSR6_0049	100
Ferric iron ABC transporter, permease protein		HTSR_0119	HSR6_0118	99.81
Iron(III) ABC transporter, periplasmic-binding protein		HTSR_0120	HSR6_0119	100
ABC transporter ATP-binding protein		HTSR_0121	HSR6_0120	98.41
Amidase clustered with urea ABC transporter and nitrile hydratase functions		HTSR_0209	HSR6_1370	36.74
ABC transporter, periplasmic spermidine putrescine-binding protein PotD (TC 3.A.1.11.1)	potD	HTSR_0210		
Putrescine transport ATP-binding protein PotA (TC 3.A.1.11.1)	potA	HTSR_0211	HSR6_2144	47.44
Spermidine Putrescine ABC transporter permease component PotB (TC 3.A.1.11.1)	potB	HTSR_0212	HSR6_2145	26.72
Spermidine Putrescine ABC transporter permease component potC (TC_3.A.1.11.1)	potC	HTSR_0213	HSR6_1703	29.61
Probable transmembrane protein (transporter component)		HTSR_0371	HSR6_0356	100
Zn-dependent hydroxyacylglutathione hydrolase		HTSR_0372	HSR6_0357	99.5
ABC-type transport system, permease component		HTSR_0373	HSR6_0358	100
ABC transporter, ATP-binding protein		HTSR_0374	HSR6_0359	100
S-layer domain (syalidase)		HTSR_0375	HSR6_0360	99.03
hypothetical protein		HTSR_0641	HSR6_0666	100
ATPase component CbiO of energizing module of cobalt ECF transporter		HTSR_0642	HSR6_0667	100
Transmembrane component CbiQ of energizing module of cobalt ECF transporter		HTSR_0643	HSR6_0668	99.26
Cobalamin transport operon protein		HTSR_0644	HSR6_0669	100
Substrate-specific component NikM of nickel ECF transporter		HTSR_0645	HSR6_0670	100
Vitamin B12 ABC transporter, ATPase component BtuD		HTSR_0646	HSR6_0671	100
Iron(III) dicitrate transport system permease protein		HTSR_0647	HSR6_0672	100
iron (III) ABC transporter, ATP-binding protein (hemV-2)		HTSR_0648	HSR6_0673	100
conserved hypothetical protein (probable surface protein)		HTSR_0692	HSR6_0718	99.79
Cell division transporter, ATP-binding protein FtsE (TC 3.A.5.1.1)		HTSR_0693	HSR6_0719	100
Cell division protein FtsX		HTSR_0694	HSR6_0720	100
Nucleoside ABC transporter, permease protein 2	rbsC2	HTSR_0793	HSR6_0820	99.72
Nucleoside ABC transporter, permease protein 1	rbsC1	HTSR_0794	HSR6_0821	98.32
ABC transporter ATP-binding protein		HTSR_0795	HSR6_0822	96.54
Nucleoside ABC transporter, periplasmic nucleoside-binding protein		HTSR_0796	HSR6_0823	100
Arsenical pump-driving ATPase (EC 3.6.3.16)	arsA	HTSR_0815	HSR6_0842	99.03
Lipid A export ATP-binding/permease protein MsbA		HTSR_0879	HSR6_0894	99.66
ABC-type nitrate/sulfonate/bicarbonate transport systems, periplasmic components		HTSR_0918	HSR6_0932	92.94
hypothetical protein		HTSR_0919	HSR6_0933	88.1
ABC-type nitrate/sulfonate/bicarbonate transport system, permease component		HTSR_0920	HSR6_0934	95.42
ABC-type nitrate/sulfonate/bicarbonate transport system ATPase component		HTSR_0921	HSR6_0935	99.62
Glutamine ABC transporter, periplasmic glutamine-binding protein (TC 3.A.1.3.2)	glnH	HTSR_1007	HSR6_1040	97.78
Polar amino acid ABC transporter, inner membrane subunit	glnP	HTSR_1008	HSR6_1041	96.51
Glutamine transport ATP-binding protein GlnQ (TC 3.A.1.3.2)	glnQ	HTSR_1009	HSR6_1042	95.63
Putative transport system permease protein (amino acid ABC transporter permease)		HTSR_1010	HSR6_1043	99.67
Permease of the drug/metabolite transporter (DMT) superfamily		HTSR_1632	HSR6_1701	100
Tungstate ABC transporter, periplasmic substrate-binding protein WtpA		HTSR_1633	HSR6_1702	94.75
Tungstate ABC transporter, permease protein WtpB		HTSR_1634	HSR6_1703	97.83
Ferric iron ABC transporter, ATP-binding protein	cysA	HTSR_1635	HSR6_1704	98.26
Substrate-specific component PdxU2 of predicted pyridoxin-related ECF transporter		HTSR_1775	HSR6_1841	82.63
Duplicated ATPase component YkoD of energizing module of thiamin-regulated ECF transporter for HydroxyMethylPyrimidine	cbiO1	HTSR_1776	HSR6_1842	93.12
ATPase component NikO of energizing module of nickel ECF transporter	cbiO2	HTSR_1777	HSR6_1843	88.93
Transmembrane component STY3231 of energizing module of queuosine-regulated ECF transporter		HTSR_1778	HSR6_1844	94.09
ABC-type nitrate/sulfonate/bicarbonate transport systems, periplasmic components		HTSR_1851	HSR6_1920	100
ABC-type nitrate/sulfonate/bicarbonate transport system, permease component		HTSR_1852	HSR6_1921	100
ABC-type nitrate/sulfonate/bicarbonate transport system ATPase component; sulfonate ABC transporter ATP binding protein(EC:3.6.3.25)		HTSR_1853	HSR6_1922	98.83
ABC-type tungstate transport system, ATP-binding protein	tupc	HTSR_1924	HSR6_1995	65.06
ABC-type tungstate transport system, permease protein	tupA	HTSR_1925	HSR6_1993	70.14
ABC-type tungstate transport system, periplasmic binding protein	tupB	HTSR_1926	HSR6_1994	70.06
DNA-binding domain of ModE / Molybdate-binding domain of ModE	modE	HTSR_1927	HSR6_1996	83.33
Phosphate ABC transporter, periplasmic phosphate-binding protein PstS (TC 3.A.1.7.1)	pstS	HTSR_1928	HSR6_1997	95.67
Phosphate transport system permease protein PstC (TC 3.A.1.7.1)	pstC	HTSR_1929	HSR6_1998	97.53
Phosphate transport system permease protein PstA (TC 3.A.1.7.1)	pstA	HTSR_1930	HSR6_1999	99.06
Phosphate transport ATP-binding protein PstB (TC 3.A.1.7.1)	pstB	HTSR_1931	HSR6_2000	100
Signal recognition particle, subunit Ffh SRP54 (TC 3.A.5.1.1)		HTSR_2039	HSR6_2115	99.78
Mg/Co/Ni transporter MgtE		HTSR_2040	HSR6_2116	98.49
Mg/Co/Ni transporter MgtE		HTSR_2041	HSR6_2117	99.47
predicted transporter		HTSR_2066	HSR6_2142	97.52
Thiamin ABC transporter, substrate-binding component	thiB	HTSR_2067	HSR6_2143	94.55
Thiamin ABC transporter, transmembrane component	thiP	HTSR_2068	HSR6_2144	96.97
Putrescine transport ATP-binding protein PotA (TC 3.A.1.11.1)	thiQ	HTSR_2069	HSR6_2145	100
ABC transporter permease protein with unknown substrate		HTSR_1549	HSR6_1619	100
Caffeoyl-CoA O-methyltransferase		HTSR_1550	HSR6_1620	99.58
ABC transporter substrate-binding protein		HTSR_1551	HSR6_1621	99.75
ABC-type probable sulfate transporter, ATPase component		HTSR_1552	HSR6_1622	99.64
Alkanesulfonates transport system permease protein		HTSR_1553	HSR6_1623	100
Vitamin B12 ABC transporter, permease component BtuC	btuC	HTSR_1674	HSR6_1742	100
Vitamin B12 ABC transporter, ATPase component BtuD	btuD	HTSR_1675	HSR6_1743	99.77
Trimethylamine methyltransferase family protein		HTSR_1676	HSR6_1744	99.79
Vitamin B12 ABC transporter, B12-binding component BtuF		HTSR_1758	HSR6_1824	99.43
Substrate-specific component CbiM of cobalt ECF transporter	cbiM	HTSR_1836	HSR6_1905	99.09
Additional substrate-specific component CbiN of cobalt ECF transporter	cbiN	HTSR_1837	HSR6_1906	100
Transmembrane component CbiQ of energizing module of cobalt ECF transporter	cbiQ	HTSR_1838	HSR6_1907	98.76
ATPase component CbiO of energizing module of cobalt ECF transporter	cbiO	HTSR_1839	HSR6_1908	99.57
Potassium channel protein		HTSR_1155	HSR6_1190	99.81
Transcriptional regulator, AsnC family		HTSR_1156	HSR6_1191	99.49
Lead, cadmium, zinc and mercury transporting ATPase (EC 3.6.3.3) (EC 3.6.3.5); Copper-translocating P-type ATPase (EC 3.6.3.4)	copB	HTSR_1157	HSR6_1192	99.3
Mercuric transport protein		HTSR_1158	HSR6_1193	100
Lead, cadmium, zinc and mercury transporting ATPase (EC 3.6.3.3) (EC 3.6.3.5); Copper-translocating P-type ATPase (EC 3.6.3.4)		HTSR_0574	HSR6_0562	99.18
**Secondary transport**				
hypothetical protein (Na+/H+ antiporter (sodium:proton antiporter)		HTSR_0020	HSR6_0020	100
amino acid transporter		HTSR_0021	HSR6_0021	98.78
Probable low-affinity inorganic phosphate transporter (PiT)		HTSR_0025	HSR6_0025	100
Probable low-affinity inorganic phosphate transporter (PiT)		HTSR_0026	HSR6_0026	100

cationic amino acid transporter (UspA domain)	uspA2	HTSR_0027	HSR6_0027	95.59
Magnesium and cobalt efflux protein CorC		HTSR_0072	HSR6_0071	99.78
Probable low-affinity inorganic phosphate transporter		HTSR_0073	HSR6_0072	99.23
Probable low-affinity inorganic phosphate transporter		HTSR_0362	HSR6_0252	53.35
Probable low-affinity inorganic phosphate transporter		HTSR_0454	HSR6_0439	97.87
Sodium-and chloride-dependent transporter		HTSR_0253	HSR6_0240	95.99
Trk system potassium uptake protein TrkA (NhaP-type Na+/H+ and K+/H+ antiporter)		HTSR_0255	HSR6_0242	99.51
UPF0098 protein MTH_273 Select (phosphatidylethanolamine-binding protein)		HTSR_0256	HSR6_0243	87.33
Potassium uptake protein TrkH		HTSR_0357	HSR6_0342	98.28
Trk system potassium uptake protein TrkA		HTSR_0358	HSR6_0343	94.27
sodium/pantothenate symporter		HTSR_0394	HSR6_0379	99
Potassium uptake protein TrkH	trkH	HTSR_0461	HSR6_0446	99.81
Trk system potassium uptake protein TrkA	trkA	HTSR_0462	HSR6_0447	97.75
Sodium/calcium exchanger		HTSR_0573	HSR6_0561	96.79
Trk system potassium uptake protein TrkA		HTSR_0925	HSR6_0939	100
Sulfate permease, Trk-type		HTSR_0957	HSR6_0988	99.84
Na+/H+ antiporter NhaC		HTSR_0998	HSR6_1031	99.11
Na+/H+ antiporter	nhaC	HTSR_1314	HSR6_1387	80.08
pH adaptation potassium efflux system protein E; sodium- potassium/hydrogen antiporter subunit E		HTSR_1024	HSR6_1057	99.73
Multiple resistance and pH regulation protein F precursor		HTSR_1025	HSR6_1058	100
pH adaptation potassium efflux system protein G; sodium- potassium/hydrogen antiporter subunit G		HTSR_1026	HSR6_1059	100
pH adaptation potassium efflux system protein B1; sodium- potassium/hydrogen antiporter subunit B1		HTSR_1027	HSR6_1060	100
pH adaptation potassium efflux system protein B2; sodium- potassium/hydrogen antiporter subunit B2		HTSR_1028	HSR6_1061	100
pH adaptation potassium efflux system protein C; sodium- potassium/hydrogen antiporter subunit C		HTSR_1029	HSR6_1062	100
pH adaptation potassium efflux system protein D 1		HTSR_1030	HSR6_1063	100
pH adaptation potassium efflux system protein D 2		HTSR_1031	HSR6_1064	99.82
pH adaptation potassium efflux system protein D 3		HTSR_1032	HSR6_1065	100
TRAP transporter solute receptor, unknown substrate 1 (C4-dicarboxylate ABC transporter substrate-binding protein		HTSR_0198	HSR6_0196	100
DUF1850 domain-containing protein (C4-dicarboxylate ABC transporter)		HTSR_0199	HSR6_0197	100
TRAP transporter, 4TM/12TM fusion protein, unknown substrate 1		HTSR_0200	HSR6_0198	99.11
Sodium-dependent transporter		HTSR_1902	HSR6_1972	100
Sodium-dependent transporter		HTSR_1993	HSR6_2069	100
Glycine betaine transporter OpuD		HTSR_0234	HSR6_0221	100
Potassium voltage-gated channel subfamily KQT; possible potassium channel, VIC family		HTSR_0427	HSR6_0412	98.92
Zinc transporter, ZIP family		HTSR_0428	HSR6_0413	99.55
Magnesium and cobalt efflux protein CorC		HTSR_0429	HSR6_0414	99.1
Amino acid permease (transporter substrate cationic amino acids)		HTSR_0432	HSR6_0417	98.51
Permease of the drug/metabolite transporter (DMT) superfamily		HTSR_0490	HSR6_0474	97.68
putative sugar transporter		HTSR_0521	HSR6_0505	97.68
Mn2+ and Fe2+ transporters of the NRAMP family		HTSR_0605	HSR6_0593	99.77
Transporter (permease)		HTSR_0632	HSR6_0657	99.74
Metal transporter, ZIP family		HTSR_0949	HSR6_0949	98.94
Nitrate/nitrite transporter NarK		HTSR_0976	HSR6_1007	99.51
Permease of the drug/metabolite transporter (DMT) superfamily		HTSR_1073	HSR6_1110	99.41
Transporter		HTSR_1214	HSR6_1284	100
Phosphate transport system regulatory protein PhoU	phoU2	HTSR_1311	HSR6_1384	100
Sugar transporter		HTSR_1459	HSR6_1531	99.75
Permease of the drug/metabolite transporter (DMT) superfamily		HTSR_1632	HSR6_1701	100
Major facilitator superfamily transporter		HTSR_1637	HSR6_1706	100
Na+/solute symporter		HTSR_1708	HSR6_1779	99.6
Nitrate/nitrite transporter		HTSR_1709	HSR6_1780	100
Glycine betaine transporter OpuD		HTSR_1753	HSR6_1819	100
metal transporter family GufA protein	zupT	HTSR_1785	HSR6_1852	100
Transporter		HTSR_1948	HSR6_2023	99.48
Putative preQ0 transporter		HTSR_1987	HSR6_2063	100
Multi antimicrobial extrusion protein (Na(+)/drug antiporter), MATE family of MDR efflux pumps		HTSR_1266	HSR6_1339	100
Cation-transporting ATPase, E1-E2 family		HTSR_1250	HSR6_1322	96.58
Amino acid permease		HTSR_1324	HSR6_1397	100
hypothetical protein (sugar phosphate permease)		HTSR_1108	HSR6_1145	98.81
Amino acid permease		HTSR_0446	HSR6_0431	97.54
Probable Co/Zn/Cd efflux system membrane fusion protein		HTSR_1319	HSR6_1392	100
oxalate/formate antiporter (oxlT-2)		HTSR_1713	HSR6_1784	98.37
**Carbohydrate metabolism**				
**Glycolysis**				
Glucose-6-phosphatase(EC:3.1.3.9)		HTSR_0832	HSR6_0859	97.24
Glucose-6-phosphate isomerase (EC 5.3.1.9)	pgi	HTSR_0138	HSR6_0139	100
Fructose-1,6-bisphosphatase, type I (EC 3.1.3.11)	fbp	HTSR_1162	HSR6_1197	100
Fructose-bisphosphate aldolase, archaeal class I (EC 4.1.2.13)	fba	HTSR_1163	HSR6_1198	100
Triosephosphate isomerase (EC 5.3.1.1)	tpiA	HTSR_0915	HSR6_0929	99.06
NAD(P)-dependent glyceraldehyde 3-phosphate dehydrogenase archaeal (EC 1.2.1.59)	gapB	HTSR_0246	HSR6_0233	100
Phosphoglycerate kinase (EC 2.7.2.3)	pgk	HTSR_0715	HSR6_0741	99.75
2,3-bisphosphoglycerate-independent phosphoglycerate mutase (EC 5.4.2.1)	gpmI	HTSR_1830	HSR6_1899	100
Enolase (EC 4.2.1.11)	eno	HTSR_0767	HSR6_0793	100
Pyruvate kinase (EC 2.7.1.40)	pykF	HTSR_1429	HSR6_1501	100
**Gluconeogenesis**				
Phosphoenolpyruvate synthase (EC 2.7.9.2)	ppsA	HTSR_1423	HSR6_1495	98
Enolase (EC 4.2.1.11)	eno	HTSR_0767	HSR6_0793	100
2,3-bisphosphoglycerate-independent phosphoglycerate mutase (EC 5.4.2.1)	gpmI	HTSR_1830	HSR6_1899	100
Phosphoglycerate kinase (EC 2.7.2.3)	pgk	HTSR_0715	HSR6_0741	99.75
NAD(P)-dependent glyceraldehyde 3-phosphate dehydrogenase archaeal (EC 1.2.1.59)	gapB	HTSR_0246	HSR6_0233	100
Triosephosphate isomerase (EC 5.3.1.1)	tpiA	HTSR_0915	HSR6_0929	99.06
Fructose-bisphosphate aldolase, archaeal class I (EC 4.1.2.13)	fba	HTSR_1163	HSR6_1198	100
Fructose-1,6-bisphosphatase, type I (EC 3.1.3.11)	fbp	HTSR_1162	HSR6_1197	100
**Glycerol metabolism**				
Anaerobic glycerol-3-phosphate dehydrogenase subunit A (EC 1.1.5.3)	glpA	HTSR_0841	HSR6_0869	98.11
Anaerobic glycerol-3-phosphate dehydrogenase subunit C (EC 1.1.5.3)	glpC	HTSR_0842	HSR6_0870	98.56
Glycerol dehydrogenase (EC 1.1.1.6)		HTSR_0114	HSR6_0113	100
Glycerol dehydrogenase (EC 1.1.1.6)	gldA	HTSR_0740	HSR6_0766	77.32
Glycerol-1-phosphate dehydrogenase [NAD(P)] (EC 1.1.1.261)	egsA	HTSR_1341	HSR6_1413	100
**Citric acid Cycle (oxidative)**				
Acetyl-coenzyme A synthetase (EC 6.2.1.1)	acs1	HTSR_1608	HSR6_1676	99.07
Citrate synthase (si) (EC 2.3.3.1)	gltA	HTSR_1879	HSR6_1949	99.74
Aconitate hydratase (EC 4.2.1.3)	acnA	HTSR_1672	HSR6_1740	99.78
Isocitrate dehydrogenase [NADP] (EC 1.1.1.42)	icd	HTSR_1855	HSR6_1924	100
2-oxoglutarate oxidoreductase, alpha subunit (EC 1.2.7.3)	korA	HTSR_0781	HSR6_0808	100
2-oxoglutarate oxidoreductase, beta subunit (EC 1.2.7.3)	korB	HTSR_0782	HSR6_0809	100
Dihydrolipoamide dehydrogenase of acetoin dehydrogenase (EC 1.8.1.4) / Dihydrolipoamide dehydrogenase (EC 1.8.1.4)	lpdA	HTSR_0182	HSR6_0180	87.47
Succinyl-CoA ligase [ADP-forming] alpha chain (EC 6.2.1.5)	sucD	HTSR_0528	HSR6_0512	99.66
Succinyl-CoA ligase [ADP-forming] beta chain (EC 6.2.1.5)	sucC	HTSR_0529	HSR6_0513	98.71
Succinate dehydrogenase flavoprotein subunit (EC 1.3.99.1)	sdhA	HTSR_0612	HSR6_0600	98.68
Succinate dehydrogenase iron-sulfur protein (EC 1.3.99.1)	sdhB	HTSR_0613	HSR6_0601	100
Succinate dehydrogenase hydrophobic membrane anchor protein	sdhD	HTSR_0614	HSR6_0602	98.36
Succinate dehydrogenase cytochrome b subunit	sdhC	HTSR_0615	HSR6_0603	97.2
succinate dehydrogenase subunit		HTSR_0616	HSR6_0604	94.12
Fumarate hydratase class II (EC 4.2.1.2)	fumC	HTSR_1079	HSR6_1116	99.79
Phosphoenolpyruvate carboxylase (EC 4.1.1.31)	ppc	HTSR_1261	HSR6_1334	99.89
NADP-dependent malic enzyme (EC 1.1.1.40)	maeB	HTSR_0493	HSR6_0477	99.74
**Respiration**				
V-type ATP synthase subunit D (EC 3.6.3.14)	atpD	HTSR_1802	HSR6_1871	100
5-methyltetrahydropteroyltriglutamate--homocysteine methyltransferase (EC 2.1.1.14)	metE	HTSR_1803	HSR6_1872	99.43
V-type ATP synthase subunit B (EC 3.6.3.14)	atpB	HTSR_1804	HSR6_1873	99.79
V-type ATP synthase subunit A (EC 3.6.3.14)	atpA	HTSR_1805	HSR6_1874	100
V-type ATP synthase subunit F (EC 3.6.3.14)	atpF	HTSR_1806	HSR6_1875	100
V-type ATP synthase subunit C (EC 3.6.3.14)	atpC	HTSR_1807	HSR6_1876	100

V-type ATP synthase subunit E (EC 3.6.3.14)	atpE	HTSR_1808	HSR6_1877	100
V-type ATP synthase subunit K (EC 3.6.3.14)	atpK	HTSR_1809	HSR6_1878	100
V-type ATP synthase subunit I (EC 3.6.3.14)	atpI	HTSR_1810	HSR6_1879	99.04
V-type ATP synthase subunit G (EC 3.6.3.14)		HTSR_1811	HSR6_1880	100
Cytochrome d ubiquinol oxidase subunit I (EC 1.10.3.-)	cydA	HTSR_0807	HSR6_0834	100
Cytochrome d ubiquinol oxidase subunit II (EC 1.10.3.-)	cydB	HTSR_0808	HSR6_0835	96.68
Transport ATP-binding protein CydC		HTSR_1769	HSR6_1835	99.84
Polysulfide reductase, subunit C, putative		HTSR_1345	HSR6_1417	100
Anaerobic dimethyl sulfoxide reductase chain B (EC 1.8.99.-)		HTSR_1346	HSR6_1418	100
Polysulfide reductase, subunit C, putative		HTSR_1697	HSR6_1766	97.78
Polysulfide reductase, subunit C, putative		HTSR_1523	HSR6_1594	100
Anaerobic dimethyl sulfoxide reductase chain B (EC 1.8.99.-)	dmsB2	HTSR_1698	HSR6_1767	100
4Fe-4S ferredoxin iron-sulfur binding domain protein		HTSR_1700	HSR6_1769	100
Uncharacterized protein DmsE associated with anaerobic dimethyl sulfoxide respiration and trimethylamine-N-oxide respiration		HTSR_0422	HSR6_0407	97.3
Anaerobic dehydrogenases, typically selenocysteine-containing (dimethylsulfoxide reductase)		HTSR_0423	HSR6_0408	99.76
Anaerobic dimethyl sulfoxide reductase chain B (EC 1.8.99.-) @ Respiratory trimethylamine-N-oxide reductase, iron-sulfur subunit		HTSR_0424	HSR6_0409	100
Anaerobic dimethyl sulfoxide reductase, membrane subunit @ Respiratory trimethylamine-N-oxide reductase, membrane subunit		HTSR_0425	HSR6_0410	100
Anaerobic dimethyl sulfoxide reductase chaperone DmsD @ Chaperone protein TorD		HTSR_0426	HSR6_0411	100
Anaerobic dimethyl sulfoxide reductase chaperone DmsD @ Chaperone protein TorD		HTSR_0514	HSR6_0498	99.45
Anaerobic dimethyl sulfoxide reductase chaperone DmsD @ Chaperone protein TorD		HTSR_1344	HSR6_1416	99.09
Anaerobic dimethyl sulfoxide reductase chaperone DmsD @ Chaperone protein TorD		HTSR_1521	HSR6_1592	99.55
Anaerobic dimethyl sulfoxide reductase chaperone DmsD @ Chaperone protein TorD		HTSR_1572	HSR6_1641	100
Polysulphide reductase, NrfD		HTSR_0515	HSR6_0499	100
Anaerobic dimethyl sulfoxide reductase chain B (EC 1.8.99.-) @ Respiratory trimethylamine-N-oxide reductase, iron-sulfur subunit		HTSR_0516	HSR6_0500	100
Anaerobic dehydrogenases, typically selenocysteine-containing (dimethylsulfoxide reductase)		HTSR_0517	HSR6_0501	99.88
Uncharacterized protein DmsE associated with anaerobic dimethyl sulfoxide respiration and trimethylamine-N-oxide respiration		HTSR_0518	HSR6_0502	100
Formate dehydrogenase -O, gamma subunit (EC 1.2.1.2)		HTSR_0751	HSR6_0777	59.49
hypothetical protein (cytochrome b subunit of formate dehydrogenase)		HTSR_1573	HSR6_1642	99.67
Formate dehydrogenase-O, iron-sulfur subunit (EC 1.2.1.2); Putative formate dehydrogenase iron-sulfur subunit (EC 1.2.1.2)	fdoH	HTSR_1575	HSR6_1644	98.91
Formate dehydrogenase-O, major subunit (EC 1.2.1.2)	fdoG	HTSR_1576	HSR6_1645	98.78
Formate dehydrogenase H (EC 1.2.1.2)	fdhA	HTSR_1736	HSR6_1802	98.97
Formate dehydrogenase (EC 1.2.1.2) (molybdopterin oxidoreductase)		HTSR_0627	-	-
Tetrathionate reductase subunit B		HTSR_0628	-	-
Sulfite reduction-associated complex DsrMKJOP protein DsrP (= HmeB)		HTSR_0629	-	-
Anaerobic dimethyl sulfoxide reductase chaperone DmsD @ Chaperone protein TorD		HTSR_0630	-	-
O-acetylhomoserine sulfhydrylase (EC 2.5.1.49) / O-succinylhomoserine sulfhydrylase (EC 2.5.1.48)		HTSR_0631	HSR6_0656	100
Trimethylamine methyltransferase family protein	mttB	HTSR_1738	HSR6_1804	100
Formate--tetrahydrofolate ligase (EC 6.3.4.3)	fhs	HTSR_1739	HSR6_1805	99.65
Formate dehydrogenase-O, major subunit (EC 1.2.1.2)	fdhA2	HTSR_1740	HSR6_1806	100
Arsenate reductase (EC 1.20.4.1)	arsC	HTSR_0173	HSR6_0169	94.08
Fe-S-cluster-containing hydrogenase components 1		HTSR_1524	HSR6_1595	100
Anaerobic dimethyl sulfoxide reductase chain B (EC 1.8.5.3)	dmsB	HTSR_1660	HSR6_1728	77.5
Anaerobic dehydrogenases, typically selenocysteine-containing (molybdopterin oxidoreductase)		HTSR_1347	HSR6_1419	100
Anaerobic dehydrogenases, typically selenocysteine-containing (molybdopterin oxidoreductase)		HTSR_1522	HSR6_1593	
Anaerobic dehydrogenases, typically selenocysteine-containing (molybdopterin oxidoreductase)		HTSR_1661	HSR6_1729	54.23
Anaerobic dehydrogenases, typically selenocysteine-containing (molybdopterin oxidoreductase)		HTSR_1699	HSR6_1768	100
4Fe-4S ferredoxin iron-sulfur binding domain protein		HTSR_1662	HSR6_1730	98.15
NADH-ubiquinone oxidoreductase chain N (EC 1.6.5.3)	nuoN	HTSR_1171	HSR6_1206	100
NADH-ubiquinone oxidoreductase chain M (EC 1.6.5.3)	nuoM	HTSR_1172	HSR6_1207	100
NADH-ubiquinone oxidoreductase chain L (EC 1.6.5.3)	nuoL	HTSR_1173	HSR6_1208	100
NADH-ubiquinone oxidoreductase chain K (EC 1.6.5.3)	nuoK	HTSR_1174	HSR6_1209	100
NADH dehydrogenase-like complex, subunit J2	nuoJ2	HTSR_1175	HSR6_1210	100
NADH-ubiquinone oxidoreductase chain J (EC 1.6.5.3)	nuoJ	HTSR_1176	HSR6_1211	100
NADH-ubiquinone oxidoreductase chain I (EC 1.6.5.3)	nuoI	HTSR_1177	HSR6_1212	100
NADH-ubiquinone oxidoreductase chain H (EC 1.6.5.3)	nuoH	HTSR_1178	HSR6_1213	100
NADH-ubiquinone oxidoreductase chain C (EC 1.6.5.3) / NADH-ubiquinone oxidoreductase chain D (EC 1.6.5.3)	nuoC	HTSR_1179	HSR6_1214	99.46
NADH-ubiquinone oxidoreductase chain B (EC 1.6.5.3)	nuoB	HTSR_1180	HSR6_1215	99.57
NADH ubiquinone oxidoreductase chain A (EC 1.6.5.3)	nuoA	HTSR_1181	HSR6_1216	100
Glycerol dehydrogenase (EC 1.1.1.6)		HTSR_0114	HSR6_0113	100
Glycerol dehydrogenase (EC 1.1.1.6)	gldA	HTSR_0740	HSR6_0766	77.32
NADH dehydrogenase (EC 1.6.99.3)		HTSR_0023	HSR6_0023	100
NADH dehydrogenase (EC 1.6.99.3)	ndh	HTSR_1044	HSR6_1077	99.74
NADH dehydrogenase (EC 1.6.99.3)	fadH2	HTSR_2023	HSR6_2099	100
Pyridine nucleotide-disulfide oxidoreductase; NADH dehydrogenase (EC 1.6.99.3)		HTSR_1094	HSR6_1131	100
Glycerol-1-phosphate dehydrogenase [NAD(P)] (EC 1.1.1.261)	egsA	HTSR_1341	HSR6_1413	100
Anaerobic glycerol-3-phosphate dehydrogenase subunit A (EC 1.1.5.3)	glpA	HTSR_0841	HSR6_0869	98.11
Anaerobic glycerol-3-phosphate dehydrogenase subunit C (EC 1.1.5.3)	glpC	HTSR_0842	HSR6_0870	98.56
[NiFe] hydrogenase metallocenter assembly protein HypF	hypF	HTSR_0649	HSR6_0674	99.87
[NiFe] hydrogenase nickel incorporation-associated protein HypB	hypB	HTSR_0650	HSR6_0675	98.53
[NiFe] hydrogenase nickel incorporation protein HypA	hypA	HTSR_0651	HSR6_0676	98.57
[NiFe] hydrogenase metallocenter assembly protein HypE	hypE	HTSR_0652	HSR6_0677	100
[NiFe] hydrogenase metallocenter assembly protein HypD	hypD	HTSR_0653	HSR6_0678	99.73
[NiFe] hydrogenase metallocenter assembly protein HypC	hypC	HTSR_0654	HSR6_0679	100
Hydrogenase maturation protease (EC 3.4.24.-)		HTSR_0655	HSR6_0681	100
Ni/Fe-hydrogenase large subunit (EC 1.12.99.6)		HTSR_0657	HSR6_0683	99.81
Ni/Fe-hydrogenase small subunit (EC 1.12.5.1)		HTSR_0658	HSR6_0684	100
hypothetical protein		HTSR_0659	HSR6_0685	100
Succinate dehydrogenase flavoprotein subunit (EC 1.3.99.1)	sdhA	HTSR_0612	HSR6_0600	98.68
Succinate dehydrogenase iron-sulfur protein (EC 1.3.99.1)	sdhB	HTSR_0613	HSR6_0601	100
Succinate dehydrogenase hydrophobic membrane anchor protein	sdhD	HTSR_0614	HSR6_0602	98.36
Succinate dehydrogenase cytochrome b subunit	sdhC	HTSR_0615	HSR6_0603	97.2
succinate dehydrogenase subunit		HTSR_0616	HSR6_0604	94.12
Putative oxidoreductase YncB		HTSR_1104	HSR6_1141	99.69
soluble [2Fe-2S] ferredoxin	fdx	HTSR_1312	HSR6_1385	100
soluble [2Fe-2S] ferredoxin	fdx	HTSR_1558	HSR6_1628	99.62
Ferredoxin		HTSR_0081	HSR6_0080	100
Ferredoxin-like protein		HTSR_0167	HSR6_0163	100
Ferredoxin-like protein		HTSR_1571	HSR6_1640	99.85
Ferredoxin	uspA12	HTSR_1233	HSR6_1304	100
soluble [2Fe-2S] ferredoxin	fdx	HTSR_1312	HSR6_1385	100
Aldehyde ferredoxin oxidoreductase	aor	HTSR_1751	HSR6_1817	99.85
Tungsten-containing aldehyde:ferredoxin oxidoreductase (EC 1.2.7.5)	aor2	HTSR_1962	HSR6_2037	100
Ribulose-1,5-bisphosphate carboxylase, Type III (EC 4.1.1.39)	rbcL	HTSR_1161	HSR6_1196	100
**Fatty acid biosynthesis**				
3-oxoacyl-[acyl-carrier protein] reductase (EC 1.1.1.100)	fabG	HTSR_0229	HSR6_0216	100
3-oxoacyl-[acyl-carrier protein] reductase (EC 1.1.1.100)	fabG2	HTSR_0447	HSR6_0432	99.6
3-oxoacyl-[acyl-carrier protein] reductase (EC 1.1.1.100)	fabG1	HTSR_1202	HSR6_0432	52
3-oxoacyl-[acyl-carrier protein] reductase (EC 1.1.1.100)	fabG2	HTSR_1404	HSR6_1476	100
3-oxoacyl-[acyl-carrier protein] reductase (EC 1.1.1.100)	fabG3	HTSR_1684	HSR6_1752	100
**Amino acid biosynthesis**				
Aspartate aminotransferase (EC 2.6.1.1) (Asp)	aspC	HTSR_0414	HSR6_0399	100
Aspartate aminotransferase (EC 2.6.1.1) (Asp)	aspC3	HTSR_0975	HSR6_1006	99.73
Aspartate aminotransferase (EC 2.6.1.1) (Asp)	aspC4	HTSR_1169	HSR6_1204	100
Aspartate aminotransferase (EC 2.6.1.1) (Asp)	aspC5	HTSR_1188	HSR6_1223	98.48
Aspartate aminotransferase (EC 2.6.1.1) (Asp)	aspC2	HTSR_0784	HSR6_0811	99.7
NADP-specific glutamate dehydrogenase (EC 1.4.1.4) (Glu)	gdhA	HTSR_1189	HSR6_1224	99.28
Glutamine synthetase type I (EC 6.3.1.2) (Gln)	glnA	HTSR_1888	HSR6_1958	99.34
N-acetylglutamate synthase (EC 2.3.1.1); Lysine biosynthesis protein LysX (Arg)	lysX	HTSR_2057	HSR6_2133	100
N-acetyl-gamma-aminoadipyl-phosphate reductase (EC 1.2.1.-); N-acetyl-gamma-glutamyl-phosphate reductase (EC 1.2.1.38) (Arg)	argC	HTSR_0665	HSR6_0691	98.27
Acetylglutamate kinase (EC 2.7.2.8) @ Acetylaminoadipate kinase (EC 2.7.2.-) (Arg)	argB	HTSR_0666	HSR6_0692	99.27
Acetylornithine aminotransferase (EC 2.6.1.11); N-acetyl-lysine aminotransferase (EC 2.6.1.-) (Arg)	argD	HTSR_0667	HSR6_0693	99.73
Acetylornithine deacetylase (EC 3.5.1.16); N-acetyl-lysine deacetylase (EC 3.5.1.-) (Arg)	argE	HTSR_0668	HSR6_0694	99.7
Ornithine carbamoyltransferase (EC 2.1.3.3) (Arg)	argF	HTSR_1006	HSR6_1039	100
Argininosuccinate synthase (EC 6.3.4.5) (Arg)	argG	HTSR_2054	HSR6_2130	100

Argininosuccinate lyase (EC 4.3.2.1) (Arg)	argH	HTSR_2055	HSR6_2131	98.59
Pyrroline-5-carboxylate reductase (EC 1.5.1.2) (Pro)	proC	HTSR_1054	HSR6_1087	100
Glutamate 5-kinase (EC 2.7.2.11) (Pro)	proB	HTSR_1055	HSR6_1088	99.3
Gamma-glutamyl phosphate reductase (EC 1.2.1.41) (Pro)	proA	HTSR_1056	HSR6_1089	99.55
Ornithine cyclodeaminase (EC 4.3.1.12) (Pro)		HTSR_0178	HSR6_0176	99.36
Aspartokinase (EC 2.7.2.4) (Asn - Lsn)	lysC	HTSR_0006	HSR6_0006	100
Aspartate-semialdehyde dehydrogenase (EC 1.2.1.11) (Thr Lys)	asd	HTSR_0538	HSR6_0522	99.71
4-hydroxy-tetrahydrodipicolinate synthase (EC 4.3.3.7) (Lys)	dapA	HTSR_1329	HSR6_1401	100
Homoserine dehydrogenase (EC 1.1.1.3) (Thr)	lysC	HTSR_1561	HSR6_1631	100
Homoserine kinase (EC 2.7.1.39) (Thr)	thrB	HTSR_0379	HSR6_0364	99.66
Threonine synthase (EC 4.2.3.1) (Thr)	thrC	HTSR_1004	HSR6_1037	99.23
Threonine synthase (EC 4.2.3.1) (Thr)	thrC2	HTSR_1058	HSR6_1091	98.78
Threonine synthase (EC 4.2.3.1) (Thr)	thrC3	HTSR_1876	HSR6_1946	98.68
Threonine synthase (EC 4.2.3.1) (Thr)	thrC3	HTSR_2058	HSR6_2134	99.52
Homoserine O-acetyltransferase (EC 2.3.1.31) (Met)	metX	HTSR_2059	HSR6_2135	99.74
Lysine Biosynthetic Amino Acid Carrier Protein LysW (Met)	lysW	HTSR_2056	HSR6_2132	100
O-acetylhomoserine sulfhydrylase (EC 2.5.1.49) / O-succinylhomoserine sulfhydrylase (EC 2.5.1.48) (Met)		HTSR_0631	HSR6_0656	100
O-acetylhomoserine sulfhydrylase (EC 2.5.1.49) / O-succinylhomoserine sulfhydrylase (EC 2.5.1.48) (Met)	metY	HTSR_1687	HSR6_1755	99.3
5-methyltetrahydropteroyltriglutamate--homocysteine methyltransferase (EC 2.1.1.14) (Met)		HTSR_1803	HSR6_1872	99.43
Diaminopimelate decarboxylase (EC 4.1.1.20) (Lys)	lysA	HTSR_1332	HSR6_1404	99.75
Diaminopimelate epimerase (EC 5.1.1.7) (Lys)	dapF	HTSR_1333	HSR6_1405	99.26
Probable succinyl-diaminopimelate desuccinylase,DapE (EC 3.5.1.18) (Lys)	dapE	HTSR_1334	HSR6_1406	100
4-hydroxy-tetrahydrodipicolinate synthase (EC 4.3.3.7) (Lys)	dapA	HTSR_1329	HSR6_1401	100
4-hydroxy-tetrahydrodipicolinate reductase (EC 1.17.1.8) (Lys)	dapB	HTSR_1330	HSR6_1402	100
2,3,4,5-tetrahydropyridine-2,6-dicarboxylate N-succinyltransferase (EC 2.3.1.117) (Lys)	dapD	HTSR_1331	HSR6_1403	100
Fructose-bisphosphate aldolase, archaeal class I (EC 4.1.2.13) (Trp)	fba	HTSR_1163	HSR6_1198	100
5-Enolpyruvylshikimate-3-phosphate synthase (EC 2.5.1.19) (Trp)	aroA	HTSR_0725	HSR6_0751	94.38
3-dehydroquinate dehydratase I (EC 4.2.1.10) (Trp)	aroD	HTSR_1436	HSR6_1508	100
Shikimate 5-dehydrogenase I alpha (EC 1.1.1.25) (Trp)	aroE	HTSR_1392	HSR6_1464	100
Shikimate kinase II (EC 2.7.1.71) (Trp)	aroK	HTSR_0733	HSR6_0759	98.84
5-Enolpyruvylshikimate-3-phosphate synthase (EC 2.5.1.19) (Trp)	aroA	HTSR_0725	HSR6_0751	98.95
Chorismate synthase (EC 4.2.3.5) (Trp)	aroC	HTSR_0724	HSR6_0750	94.38
Anthranilate synthase, aminase component (EC 4.1.3.27) (Trp)	trpE1	HTSR_0479	HSR6_0463	100
Anthranilate synthase, amidotransferase component (EC 4.1.3.27) (Trp)	trpE2	HTSR_0480	HSR6_0464	98.99
Anthranilate phosphoribosyltransferase (EC 2.4.2.18) (Trp)	trpD	HTSR_0477	HSR6_0461	99.7
Phosphoribosylanthranilate isomerase (EC 5.3.1.24) (Trp)	trpF	HTSR_0478	HSR6_0462	99.52
Indole-3-glycerol phosphate synthase (EC 4.1.1.48) (Trp)	trpC	HTSR_1442	HSR6_1514	89.76
Tryptophan synthase beta chain (EC 4.2.1.20) (Trp)	trpB	HTSR_0028	HSR6_0028	100
Tryptophan synthase alpha chain (EC 4.2.1.20) (Trp)	trpA	HTSR_1440	HSR6_1512	98.08
Tryptophan synthase beta chain (EC 4.2.1.20) (Trp)	trpB	HTSR_1441	HSR6_1513	100
3-deoxy-manno-octulosonate cytidylyltransferase (EC 2.7.7.38)	kdsB	HTSR_0858	HSR6_0875	74.56
2-Keto-3-deoxy-D-manno-octulosonate-8-phosphate synthase (EC 2.5.1.55)	kdsA	HTSR_0859	HSR6_0876	92.22
Chorismate mutase I (EC 5.4.99.5) (Tyr Phe)	aroQ	HTSR_0732	HSR6_0758	100
Prephenate and/or arogenate dehydrogenase (unknown specificity) (EC 1.3.1.12)(EC 1.3.1.43) (Tyr)	tyrA	HTSR_0727	HSR6_0753	100
Biosynthetic Aromatic amino acid aminotransferase beta (EC 2.6.1.57) @ Histidinol-phosphate aminotransferase (EC 2.6.1.9) (Tyr)	hisC	HTSR_0911	HSR6_0925	96.91
Prephenate dehydratase (EC 4.2.1.51) (Phe)	pheA	HTSR_0181	HSR6_0179	98.88
Branched-chain amino acid aminotransferase (EC 2.6.1.42) (Ile)	ilvE	HTSR_0277	HSR6_0263	100
Dihydroxy-acid dehydratase (EC 4.2.1.9) (Ile)	ilvD	HTSR_1107	HSR6_1144	100
Dihydroxy-acid dehydratase (EC 4.2.1.9) (Ile)	ilvD2	HTSR_1218	HSR6_1288	100
Ketol-acid reductoisomerase (EC 1.1.1.86) (Ile)	ilvC	HTSR_0383	HSR6_0368	99.7
Acetolactate synthase small subunit (EC 2.2.1.6) (Ile)	ilvH	HTSR_0384	HSR6_0369	100
Acetolactate synthase large subunit (EC 2.2.1.6) (Ile)	ilvH2	HTSR_0385	HSR6_0370	99.83
Thiosulfate sulfurtransferase, rhodanese (EC 2.8.1.1) (Ala)	sseA	HTSR_1348	HSR6_1420	99.76
Ketol-acid reductoisomerase (EC 1.1.1.86) (Val)		HTSR_0383	HSR6_0368	99.7
Dihydroxy-acid dehydratase (EC 4.2.1.9) (Val)	ilvD	HTSR_1107	HSR6_1144	100
Dihydroxy-acid dehydratase (EC 4.2.1.9) (Val)	ilvD2	HTSR_1218	HSR6_1288	100
Branched-chain amino acid aminotransferase (EC 2.6.1.42) (Val)		HTSR_0277	HSR6_0263	100
2-isopropylmalate synthase (EC 2.3.3.13) (Leu)	leuA	HTSR_0386	HSR6_0371	100
2-isopropylmalate synthase (EC 2.3.3.13) (Leu)	leuA2	HTSR_1495	HSR6_1566	99.4
3-isopropylmalate dehydrogenase (EC 1.1.1.85) (Leu)	leuB	HTSR_0380	HSR6_0365	99.69
3-isopropylmalate dehydratase small subunit (EC 4.2.1.33) (Leu)	leuD	HTSR_0381	HSR6_0366	100
3-isopropylmalate dehydratase large subunit (EC 4.2.1.33) (Leu)	leuC	HTSR_0382	HSR6_0367	99.15
Branched-chain amino acid aminotransferase (EC 2.6.1.42) (Leu)		HTSR_0277	HSR6_0263	100
D-3-phosphoglycerate dehydrogenase (EC 1.1.1.95)	serA	HTSR_0539	HSR6_0523	99.37
D-3-phosphoglycerate dehydrogenase (EC 1.1.1.95)	gyaR	HTSR_1168	HSR6_1203	100
Threonine dehydratase (EC 4.3.1.19) (Ser)	ilvA	HTSR_1880	HSR6_1950	100
Phosphoserine phosphatase (EC 3.1.3.3) (Ser)	pspH	HTSR_1388	HSR6_1460	100
Serine hydroxymethyltransferase (EC 2.1.2.1) (Gly)	glyA	HTSR_0671	HSR6_0697	100
Serine acetyltransferase (EC 2.3.1.30) (Cys)	cysE	HTSR_0237	HSR6_0224	99.65
Cysteine synthase (EC 2.5.1.47) (Cys)	cysK	HTSR_1258	HSR6_1331	97.07
Cysteine synthase (EC 2.5.1.47) (Cys)	cysK2	HTSR_1625	HSR6_1693	99.68
O-acetylhomoserine sulfhydrylase (EC 2.5.1.49) / O-succinylhomoserine sulfhydrylase (EC 2.5.1.48) (Cys)		HTSR_0631	HSR6_0656	100
O-acetylhomoserine sulfhydrylase (EC 2.5.1.49) / O-succinylhomoserine sulfhydrylase (EC 2.5.1.48) (Cys)	metY	HTSR_1687		
D-3-phosphoglycerate dehydrogenase (EC 1.1.1.95) (His)	serA	HTSR_0539	HSR6_0523	99.37
D-3-phosphoglycerate dehydrogenase (EC 1.1.1.95) (His)		HTSR_1168	HSR6_1203	100
ATP phosphoribosyltransferase (EC 2.4.2.17) (His)	hisG	HTSR_0162	HSR6_0158	100
Phosphoribosyl-ATP pyrophosphatase (EC 3.6.1.31) (His)	hisE	HTSR_1616	HSR6_1684	100
Phosphoribosyl-AMP cyclohydrolase (EC 3.5.4.19) (His)	hisL	HTSR_0083	HSR6_0082	97.52
Phosphoribosylformimino-5-aminoimidazole carboxamide ribotide isomerase (EC 5.3.1.16) (His)	hisA	HTSR_0080	HSR6_0079	99.59
Imidazole glycerol phosphate synthase amidotransferase subunit (EC 2.4.2.-) (His)	hisH	HTSR_1894	HSR6_1964	100
Imidazoleglycerol-phosphate dehydratase (EC 4.2.1.19) (His)	hisB	HTSR_0079	HSR6_0078	98.97
Biosynthetic Aromatic amino acid aminotransferase beta (EC 2.6.1.57) @ Histidinol-phosphate aminotransferase (EC 2.6.1.9) (His)	hisC	HTSR_0911	HSR6_0925	96.91
Histidinol dehydrogenase (EC 1.1.1.23) (His)	hisD	HTSR_0555	HSR6_0542	98.12
Imidazole glycerol phosphate synthase cyclase subunit (EC 4.1.3.-) (His)	hisF	HTSR_1303	HSR6_1376	100
**Amino acid catabolism**				
Pyruvoyl-dependent arginine decarboxylase (EC 4.1.1.19) (Arg)	pdaD	HTSR_0131	HSR6_0130	100
Agmatinase (EC 3.5.3.11) (Arg)	speB	HTSR_0416	HSR6_0401	100
Tryptophanase (EC 4.1.99.1) (Trp)	tnaA	HTSR_0012	HSR6_0012	99.545
Branched-chain amino acid aminotransferase (EC 2.6.1.42) (Ile, Leu,Val)		HTSR_0277	HSR6_0263	100
Dihydrolipoamide dehydrogenase of acetoin dehydrogenase (EC 1.8.1.4) / Dihydrolipoamide dehydrogenase (EC 1.8.1.4)		HTSR_0182	HSR6_0180	87.47
L-tyrosine decarboxylase (EC 4.1.1.25) (Tyr)	gadD	HTSR_1425	HSR6_1497	99.72
Aminomethyltransferase (glycine cleavage system T protein) (EC 2.1.2.10) (Gly)	gcvT	HTSR_0503	HSR6_0487	100
Glycine cleavage system H protein (Gly)		HTSR_0504	HSR6_0488	100
Glycine dehydrogenase [decarboxylating] (glycine cleavage system P1 protein) (EC 1.4.4.2) (Gly)	gcvP1	HTSR_0505	HSR6_0489	99.77
Glycine dehydrogenase [decarboxylating] (glycine cleavage system P2 protein) (EC 1.4.4.2) (Gly)	gcvP2	HTSR_0506	HSR6_0490	99.79
Archaeal S-adenosylmethionine synthetase (EC 2.5.1.6) (Met)	metK	HTSR_0607	HSR6_0595	99
Adenosylhomocysteinase (EC 3.3.1.1) (Met)	ahcY	HTSR_0160	HSR6_0156	99.77
Cysteine desulfurase (EC 2.8.1.7) (Cys)	sufS	HTSR_1091	HSR6_1128	100
Putative iron-sulfur cluster assembly scaffold protein for SUF system, SufE2 (Cys)	nifU	HTSR_2025	HSR6_2101	96.35
**Urea degradation**				
Urea channel UreI		HTSR_0637	HSR6_0662	100
3.5.1.5 (UreB/UreC/UreA)				
UreG,UreH, UreE/UreD, UreF				
**Fatty acid biosynthesis**				
3-oxoacyl-[acyl-carrier protein] reductase (EC 1.1.1.100)	fabG	HTSR_0229	HSR6_0216	100

3-oxoacyl-[acyl-carrier protein] reductase (EC 1.1.1.100)	fabG2	HTSR_0447	HSR6_0432	99.6
3-oxoacyl-[acyl-carrier protein] reductase (EC 1.1.1.100)	fabG1	HTSR_1202	HSR6_0432	52
3-oxoacyl-[acyl-carrier protein] reductase (EC 1.1.1.100)	fabG2	HTSR_1404	HSR6_1476	100
3-oxoacyl-[acyl-carrier protein] reductase (EC 1.1.1.100)	fabG3	HTSR_1684	HSR6_1752	100
**Nitrogen metabolism**				
Nitrate/nitrite transporter NarK		HTSR_0976	HSR6_1007	99.51
Nitrite reductase probable [NAD(P)H] subunit (EC 1.7.1.4)		HTSR_0303	HSR6_0289	100
NrfC protein		HTSR_0754	SR6_0780	99.59
NrfD protein		HTSR_1557	HSR6_1627	100
Nitrate/nitrite transporter		HTSR_1709	HSR6_1780	100
ABC-type nitrate/sulfonate/bicarbonate transport systems, periplasmic components		HTSR_0918	HSR6_0932	92.94
hypothetical protein		HTSR_0919	HSR6_0933	88.1
ABC-type nitrate/sulfonate/bicarbonate transport system, permease component		HTSR_0920	HSR6_0934	95.42
ABC-type nitrate/sulfonate/bicarbonate transport system ATPase component		HTSR_0921	HSR6_0935	99.62
ABC-type nitrate/sulfonate/bicarbonate transport systems, periplasmic components		HTSR_1851	HSR6_1920	100
ABC-type nitrate/sulfonate/bicarbonate transport system, permease component		HTSR_1852	HSR6_1921	100
ABC-type nitrate/sulfonate/bicarbonate transport system ATPase component; sulfonate ABC transporter ATP binding protein(EC:3.6.3.25)		HTSR_1853	HSR6_1922	98.83
Molybdopterin-guanine dinucleotide biosynthesis protein MobB	mobB	HTSR_1147	HSR6_1182	100
Molybdopterin-guanine dinucleotide biosynthesis protein MobA	mobA	HTSR_1664	HSR6_1732	99.43
Molybdopterin-guanine dinucleotide biosynthesis protein MobB / Molybdenum cofactor biosynthesis protein MoaE	moaE	HTSR_1951	HSR6_2026	98.07
Nitric oxide reductase activation protein NorQ		HTSR_0389	HSR6_0374	100
Nitric oxide reductase activation protein NorQ		HTSR_1679	HSR6_1747	99.1
Glutamine synthetase type I (EC 6.3.1.2)	glnA	HTSR_1888	HSR6_1958	99.34
N-acetylglutamate synthase (EC 2.3.1.1); Lysine biosynthesis protein LysX	lysX	HTSR_2057	HSR6_2133	100
NADP-specific glutamate dehydrogenase (EC 1.4.1.4)	gdhA	HTSR_1189	HSR6_1224	99.28
nifU protein C-terminal domain homolog		HTSR_1597	HSR6_1666	100
Capsular polysaccharide biosynthesis / N-glycosylation				
Glucosamine--fructose-6-phosphate aminotransferase [isomerizing] (EC 2.6.1.16)	glmS	HTSR_0882	HSR6_0897	99.67
N-acetylglucosamine-1-phosphate uridyltransferase (EC 2.7.7.23) / Glucosamine-1-phosphate N-acetyltransferase (EC 2.3.1.157)	glmU	HTSR_0890	HSR6_0906	99.24
UDP-glucose dehydrogenase (EC 1.1.1.22)	ugd	HTSR_0878	HSR6_0893	95.83
transmembrane oligosaccharyl transferase	aglB	HTSR_0845	HSR6_0872	87.82
**Osmotic balance - homeostasis**				
Trk system potassium uptake protein TrkA (NhaP-type Na+/H+ and K+/H+ antiporter)		HTSR_0255	HSR6_0242	99.51
Potassium uptake protein TrkH		HTSR_0357	HSR6_0342	98.28
Trk system potassium uptake protein TrkA		HTSR_0358	HSR6_0343	94.27
Potassium uptake protein TrkH	trkH	HTSR_0461	HSR6_0446	99.81
Trk system potassium uptake protein TrkA	trkA	HTSR_0462	HSR6_0447	97.75
Trk system potassium uptake protein TrkA		HTSR_0925	HSR6_0939	100
Sulfate permease, Trk-type		HTSR_0957	HSR6_0988	99.84
Trk system potassium uptake protein TrkA (NhaP-type Na+/H+ and K+/H+ antiporter)		HTSR_0255	HSR6_0242	99.51
Na+/H+ antiporter NhaC		HTSR_0998	HSR6_1031	99.11
Na+/H+ antiporter	nhaC	HTSR_1314	HSR6_1387	80.08
Sodium/calcium exchanger		HTSR_0573	HSR6_0561	96.79
PH adaptation potassium efflux system protein E; sodium- potassium/hydrogen antiporter subunit E (multisubunit Na+/H+ antiporter, Mnh)		HTSR_1024	HSR6_1057	99.73
Multiple resistance and pH regulation protein F precursor (multisubunit Na+/H+ antiporter, Mnh)		HTSR_1025	HSR6_1058	100
PH adaptation potassium efflux system protein G; sodium- potassium/hydrogen antiporter subunit G (multisubunit Na+/H+ antiporter, Mnh)		HTSR_1026	HSR6_1059	100
PH adaptation potassium efflux system protein B1; sodium- potassium/hydrogen antiporter subunit B1 (multisubunit Na+/H+ antiporter, Mnh)		HTSR_1027	HSR6_1060	100
PH adaptation potassium efflux system protein B2; sodium- potassium/hydrogen antiporter subunit B2 (multisubunit Na+/H+ antiporter, Mnh)		HTSR_1028	HSR6_1061	100
PH adaptation potassium efflux system protein C; sodium- potassium/hydrogen antiporter subunit C (multisubunit Na+/H+ antiporter, Mnh)		HTSR_1029	HSR6_1062	100
PH adaptation potassium efflux system protein D 1 (multisubunit Na+/H+ antiporter, Mnh)		HTSR_1030	HSR6_1063	100
pH adaptation potassium efflux system protein D 2 (multisubunit Na+/H+ antiporter, Mnh)		HTSR_1031	HSR6_1064	99.82
pH adaptation potassium efflux system protein D 3 (multisubunit Na+/H+ antiporter, Mnh)		HTSR_1032	HSR6_1065	100
High-affinity choline uptake protein BetT		HTSR_0054	HSR6_0054	100
Glycine betaine transporter OpuD		HTSR_0234	HSR6_0221	100
Choline-sulfatase (EC 3.1.6.6)		HTSR_0994	HSR6_1027	96.10
Glycine betaine transporter OpuD		HTSR_1753	HSR6_1819	100
Carbonic anhydrase (EC 4.2.1.1)	cynT	HTSR_0257	HSR6_0244	90.79
**Rhodopsins**				
Phytoene synthase (EC 2.5.1.32)	crtB	HTSR_0319	HSR6_0305	98.74
putative carotenoid biosynthesis protein		HTSR_0320	HSR6_0306	100
Lycopene elongase (EC 2.5.1.-)	ubiA	HTSR_0321	HSR6_0307	100
Phytoene dehydrogenase (EC 1.14.99.-)	crtI	HTSR_0322	HSR6_0308	100
Bacterio-opsin activator domain-containing protein		HTSR_0056	HSR6_0056	100
Bacterio-opsin activator domain-containing protein		HTSR_0057	HSR6_0057	98.57
bacterio-opsin activator HTH domain-containing protein		HTSR_0421	HSR6_0406	100
bacterio-opsin activator HTH domain-containing protein		HTSR_0520	HSR6_0504	99.02
Bacterio-opsin activator domain-containing protein		HTSR_0542	HSR6_0526	100
bacterio-opsin activator HTH domain-containing protein		HTSR_0625	-	-
bacterio-opsin activator HTH domain-containing protein		HTSR_0755	HSR6_0781	99.51
Bacterio-opsin activator domain-containing protein		HTSR_0789	HSR6_0816	95.39
Bacterio-opsin activator domain-containing protein		HTSR_0790	HSR6_0817	92.59
Octaprenyl diphosphate synthase (EC 2.5.1.90) / Dimethylallyltransferase (EC 2.5.1.1) / (2E,6E)-farnesyl diphosphate synthase (EC 2.5.1.10) / Geranylgeranyl (	idsA	HTSR_1035	HSR6_1068	99.72
bacterio-opsin activator HTH domain-containing protein		HTSR_1343	HSR6_1415	99.01
bacterio-opsin activator HTH domain-containing protein		HTSR_1349	HSR6_1421	99.51
bacterio-opsin activator HTH domain-containing protein		HTSR_1350	HSR6_1422	99.53
bacterio-opsin activator HTH domain-containing protein		HTSR_1545	HSR6_1615	99.1
bacterio-opsin activator HTH domain-containing protein		HTSR_1546	HSR6_1616	99.12
bacterio-opsin activator HTH domain-containing protein		HTSR_1547	HSR6_1617	100
bacterio-opsin activator HTH domain-containing protein		HTSR_1663	HSR6_1731	100
Bacterio-opsin activator domain-containing protein		HTSR_1789	HSR6_1858	97.02
Octaprenyl diphosphate synthase (EC 2.5.1.90) / Dimethylallyltransferase (EC 2.5.1.1) / (2E,6E)-farnesyl diphosphate synthase (EC 2.5.1.10) / Geranylgeranyl (	idsA2	HTSR_1817	HSR6_1886	100
HTR-like protein		HTSR_0443	HSR6_0428	97.48
HTR-like protein		HTSR_0470	HSR6_0454	100
Htr-like protein	htlA	HTSR_0674	HSR6_0700	94.3
**Ubiquinone and other terpenoid-quinone biosynthesis**				
Ubiquinone/menaquinone biosynthesis methyltransferase UbiE/COQ5 (EC 2.1.1.-)	ubiE	HTSR_1450	HSR6_1522	97.45
2-heptaprenyl-1,4-naphthoquinone methyltransferase (EC 2.1.1.163)		HTSR_1106	HSR6_1143	99.46
2-succinyl-5-enolpyruvyl-6-hydroxy-3-cyclohexene-1-carboxylic-acid synthase (EC 2.2.1.9)	menD	HTSR_1291	HSR6_1364	99.49
Lycopene elongase (EC 2.5.1.-)	ubiA	HTSR_0321	HSR6_0307	100
1,4-dihydroxy-2-naphthoate polyprenyltransferase (EC 2.5.1.74)	menA	HTSR_1105	HSR6_1142	97.25
1,4-dihydroxy-2-naphthoate polyprenyltransferase (EC 2.5.1.74)	menA	HTSR_1293	HSR6_1366	100
Isochorismate synthase (EC 5.4.4.2) @ Menaquinone-specific isochorismate synthase (EC 5.4.4.2)	menF	HTSR_1290	HSR6_1363	99.78
Naphthoate synthase (EC 4.1.3.36)	menB	HTSR_1292	HSR6_1365	100
O-succinylbenzoate synthase (EC 4.2.1.113)	menC	HTSR_1294	HSR6_1367	100
O-succinylbenzoic acid--CoA ligase (EC 6.2.1.26)	menE	HTSR_1295	HSR6_1368	96.57
**Excision repair**				
Excinuclease ABC subunit A	uvrA	HTSR_1594	HSR6_1663	99.62
DNA ligase (EC 6.5.1.2)	ligA	HTSR_2061	HSR6_2137	98.59
unknown		HTSR_2062	HSR6_2138	98.02
Excinuclease ABC subunit C	uvrC	HTSR_2063	HSR6_2139	100
Heme-degrading monooxygenase IsdG (EC 1.14.99.3)		HTSR_2064	HSR6_2140	95.33

Excinuclease ABC subunit B	uvrB	HTSR_2065	HSR6_2141	100
ATP-dependent DNA helicase pcrA (EC 3.6.1.-)	uvrD	HTSR_1601	HSR6_1670	98.52
ATP-dependent DNA helicase pcrA (EC 3.6.1.-)		HTSR_1724	|HSR6_1794	96.23
**Oxidative stress**				
Superoxide dismutase [Mn] (EC 1.15.1.1)	sod	HTSR_1099	HSR6_1136	100
Catalase (EC 1.11.1.6) / Peroxidase (EC 1.11.1.7)	katG	HTSR_1111	HSR6_1146	100
**Flagella**				
Flagella-related protein FlaI		HTSR_0799	HSR6_0826	99.82
Flagella-related protein FlaI		HTSR_1215	HSR6_1285	99.88
flagella accessory protein FlaCE	flaE	HTSR_1353	HSR6_1425	100
Flagella-related protein FlaF	flaF	HTSR_1354	HSR6_1426	99.35
Flagella-related protein FlaG	flaG	HTSR_1355	HSR6_1427	99.33
Flagella-related protein FlaH	flaH	HTSR_1356	HSR6_1428	100
Flagella-related protein FlaI	flaI	HTSR_1357	HSR6_1429	100
Flagella-related protein FlaJ	flaJ	HTSR_1358	HSR6_1430	99.65
Flagellin FlaB2	FlaB2	HTSR_1378	HSR6_1450	98.52
hypothetical protein		HTSR_1379	HSR6_1451	99.45
Flagellin FlaB2	FlaB2	HTSR_1380	HSR6_1452	97.02
Flagellin FlaB2	FlaB2	HTSR_1381	HSR6_1453	90.86
hypothetical protein		HTSR_1382	HSR6_1454	99.29
Flagella-related protein FlaE	FlaE	HTSR_1383	HSR6_1455	100
**Gas vesicle**				
GvpA_L_F_G_H_K_O				
**Sec system**				
Protein-export membrane protein SecF (TC 3.A.5.1.1)	SecF	HTSR_0141	HSR6_0142	100
Protein-export membrane protein SecD (TC 3.A.5.1.1)	SecD	HTSR_0142	HSR6_0143	100
Preprotein translocase secY subunit (TC 3.A.5.1.1)	secY	HTSR_0354	HSR6_0339	100
Preprotein translocase subunit secE (Protein transport protein SEC61 gamma subunit homolog)	secE	HTSR_1397	HSR6_1469	100
Preprotein translocase secG subunit (Protein transport protein SEC61 subunit beta homolog)		HTSR_1614	HSR6_1682	100
**Tat complex**				
TatA-C				
