# Supplementary material for: Discovery of anaerobic lithoheterotrophic haloarchaea, ubiquitous in hypersaline habitats
Source: ISME J. 2017 Jan 20;11(5):1245–60. doi: 10.1038/ismej.2016.203 (PMC5437934; doi:10.1038/ismej.2016.203)
Supplement: Supplementary Table S11 [file ismej2016203x4.pdf]

Table S11 Proteome of Halodesulfurarchaeum formicicum HTSR1, with abundance and relative abundance (%) of proteins detected in corresponding sulfur- (red), thiosulfate- (blue) and DMSO-respiring (green) cells.

locus_tag	product	S8	THIO	HTRS	S8	THIO	HTRS
HTSR_0001	orc1/cdc6 family replication initiation protein	3	3	15	0,027%	0,028%	0,359%
HTSR_0002	hypothetical protein	0	0	0	0,000%	0,000%	0,000%
HTSR_0003	hypothetical protein	0	0	0	0,000%	0,000%	0,000%
HTSR_0004	signal peptidase I	3	3	4	0,027%	0,028%	0,096%
HTSR_0005	DNA polymerase II small subunit	0	0	3	0,000%	0,000%	0,072%
HTSR_0006	aspartate kinase	9	11	10	0,082%	0,104%	0,239%
HTSR_0007	radical SAM protein	12	6	2	0,109%	0,057%	0,048%
HTSR_0008	hypothetical protein	0	0	1	0,000%	0,000%	0,024%
HTSR_0009	hypothetical protein	0	0	0	0,000%	0,000%	0,000%
HTSR_0010	lipoate-protein ligase A	0	1	1	0,000%	0,009%	0,024%
HTSR_0011	lipoate synthase	0	0	2	0,000%	0,000%	0,048%
HTSR_0012	tryptophanase	5	7	8	0,045%	0,066%	0,191%
HTSR_0013	serine/threonine protein phosphatase	0	0	0	0,000%	0,000%	0,000%
HTSR_0014	IMP cyclohydrolase	0	0	0	0,000%	0,000%	0,000%
HTSR_0016	hypothetical protein	0	0	0	0,000%	0,000%	0,000%
HTSR_0017	permease	0	0	0	0,000%	0,000%	0,000%
HTSR_0018	universal stress protein UspA	0	0	0	0,000%	0,000%	0,000%
HTSR_0019	hypothetical protein	0	0	0	0,000%	0,000%	0,000%
HTSR_0020	Na+/H+ antiporter NhaC-like protein	1	1	0	0,009%	0,009%	0,000%
HTSR_0021	amino acid permease-associated region	6	24	5	0,054%	0,227%	0,120%
HTSR_0022	AsnC family transcriptional regulator	4	5	4	0,036%	0,047%	0,096%
HTSR_0023	NADH dehydrogenase	0	0	0	0,000%	0,000%	0,000%
HTSR_0024	hypothetical protein	6	6	4	0,054%	0,057%	0,096%
HTSR_0025	phosphate transporter	3	2	2	0,027%	0,019%	0,048%
HTSR_0026	phosphate transporter	0	0	0	0,000%	0,000%	0,000%
HTSR_0027	universal stress protein UspA	10	25	19	0,091%	0,236%	0,455%
HTSR_0028	tryptophan synthase, beta subunit	17	26	16	0,154%	0,246%	0,383%
HTSR_0029	NADH-dependent flavin oxidoreductase	12	19	10	0,109%	0,179%	0,239%
HTSR_0030	thymidylate synthase	4	7	3	0,036%	0,066%	0,072%
HTSR_0031	dihydrofolate reductase	5	0	1	0,045%	0,000%	0,024%
HTSR_0032	ferredoxin	3	2	1	0,027%	0,019%	0,024%
HTSR_0033	spermidine synthase	0	1	6	0,000%	0,009%	0,144%
HTSR_0034	3-hydroxy-3-methylglutaryl-coenzyme A reductase	24	32	16	0,218%	0,302%	0,383%
HTSR_0035	glutaredoxin	20	16	7	0,181%	0,151%	0,168%
HTSR_0036	hypothetical protein	0	0	0	0,000%	0,000%	0,000%
HTSR_0037	Uncharacterized protein MJ0187	3	0	1	0,027%	0,000%	0,024%
HTSR_0038	DEAD/DEAH box helicase domain protein/Putative ski2-type helicase MJ1124	5	9	13	0,045%	0,085%	0,311%
HTSR_0039	hypothetical protein	5	6	3	0,045%	0,057%	0,072%
HTSR_0040	universal stress protein UspA	3	5	4	0,027%	0,047%	0,096%
HTSR_0041	Hef nuclease / ATP-dependent RNA helicase, EIF-4A family	8	16	18	0,073%	0,151%	0,431%
HTSR_0042	dolichol kinase	0	0	0	0,000%	0,000%	0,000%
HTSR_0043	glycyl-tRNA synthetase	15	11	13	0,136%	0,104%	0,311%
HTSR_0044	putative signal transduction protein with CBS domains	14	17	14	0,127%	0,161%	0,335%
HTSR_0045	hypothetical protein	1	0	1	0,009%	0,000%	0,024%
HTSR_0046	oligopeptide/dipeptide ABC transporter, ATPase subunit	10	21	20	0,091%	0,198%	0,479%
HTSR_0047	peptide ABC transporter substrate-binding protein / periplasmic substrate-binding component (TC 3.A.1.5.2)	32	31	24	0,290%	0,293%	0,574%
HTSR_0048	peptide ABC transporter permease DppB (TC 3.A.1.5.2)	3	2	1	0,027%	0,019%	0,024%
HTSR_0049	peptide ABC transporter permease DppC (TC 3.A.1.5.2)	5	3	3	0,045%	0,028%	0,072%
HTSR_0050	hypothetical protein	0	0	0	0,000%	0,000%	0,000%
HTSR_0051	hypothetical protein	0	0	0	0,000%	0,000%	0,000%
HTSR_0052	hypothetical protein	1	0	1	0,009%	0,000%	0,024%
HTSR_0053	DNA polymerase II large subunit	1	5	9	0,009%	0,047%	0,215%
HTSR_0054	glycine/betaine ABC transporter	1	0	0	0,009%	0,000%	0,000%
HTSR_0055	IclR family transcriptional regulator	0	0	6	0,000%	0,000%	0,144%
HTSR_0056	bacterio-opsin activator HTH domain-containing protein	6	0	2	0,054%	0,000%	0,048%
HTSR_0057	bacterio-opsin activator HTH domain-containing protein	2	0	0	0,018%	0,000%	0,000%
HTSR_0058	phosphoesterase RecJ domain-containing protein / Single-stranded-DNA-specific exonuclease RecJ (EC 3.1.-.-)	0	0	10	0,000%	0,000%	0,239%
HTSR_0059	uroporphyrinogen III methyltransferase	2	3	3	0,018%	0,028%	0,072%
HTSR_0060	porphobilinogen deaminase	15	22	15	0,136%	0,208%	0,359%
HTSR_0061	glutamate-1-semialdehyde aminotransferase	5	8	6	0,045%	0,076%	0,144%
HTSR_0062	glutamate-1-semialdehyde aminotransferase	4	6	7	0,036%	0,057%	0,168%
HTSR_0063	hypothetical protein	0	0	0	0,000%	0,000%	0,000%
HTSR_0064	delta-aminolevulinic acid dehydratase / Porphobilinogen synthase (EC 4.2.1.24)	17	13	9	0,154%	0,123%	0,215%
HTSR_0065	Fe-S oxidoreductase	18	34	25	0,163%	0,321%	0,598%
HTSR_0066	hypothetical protein	1	0	0	0,009%	0,000%	0,000%
HTSR_0067	hypothetical protein	0	0	0	0,000%	0,000%	0,000%
HTSR_0068	Sua5/YciO/YrdC/YwlC family protein	2	1	0	0,018%	0,009%	0,000%
HTSR_0069	hypothetical protein	0	0	0	0,000%	0,000%	0,000%
HTSR_0070	peroxiredoxin-like protein	6	1	2	0,054%	0,009%	0,048%
HTSR_0071	glutaredoxin / Glutathione S-transferase N-terminal domain	6	2	4	0,054%	0,019%	0,096%
HTSR_0072	hypothetical protein / Magnesium and cobalt efflux protein CorC	10	9	10	0,091%	0,085%	0,239%
HTSR_0073	phosphate transporter	1	1	1	0,009%	0,009%	0,024%
HTSR_0074	hypothetical protein	7	4	2	0,064%	0,038%	0,048%
HTSR_0075	uracil phosphoribosyltransferase	6	10	6	0,054%	0,094%	0,144%
HTSR_0076	hypothetical protein	0	0	0	0,000%	0,000%	0,000%
HTSR_0077	hypothetical protein / FIG000605: protein co-occurring with transport systems (COG1739)	1	9	15	0,009%	0,085%	0,359%
HTSR_0078	amino acid-binding ACT domain-containing protein	8	4	4	0,073%	0,038%	0,096%
HTSR_0079	imidazoleglycerol-phosphate dehydratase	2	4	3	0,018%	0,038%	0,072%
HTSR_0080	phosphoribosylformimino-5-aminoimidazole carboxamide ribotide isomerase	2	11	5	0,018%	0,104%	0,120%
HTSR_0081	ferredoxin	3	10	2	0,027%	0,094%	0,048%
HTSR_0082	peptidase A24B, FlaK domain protein / Signal peptidase, type IV - prepilin/preflagellin	3	3	4	0,027%	0,028%	0,096%
HTSR_0083	phosphoribosyl-AMP cyclohydrolase	3	0	1	0,027%	0,000%	0,024%
HTSR_0084	hypothetical protein	24	13	16	0,218%	0,123%	0,383%
HTSR_0085	MCM family protein	15	15	14	0,136%	0,142%	0,335%
HTSR_0086	hypothetical protein	0	0	1	0,000%	0,000%	0,024%
HTSR_0087	hypothetical protein	0	0	0	0,000%	0,000%	0,000%
HTSR_0088	hypothetical protein	0	0	0	0,000%	0,000%	0,000%
HTSR_0089	hypothetical protein	0	0	0	0,000%	0,000%	0,000%
HTSR_0090	cell division inhibitor MinD-like	0	0	0	0,000%	0,000%	0,000%
HTSR_0091	transcription initiation factor TFB	0	0	0	0,000%	0,000%	0,000%
HTSR_0092	hypothetical protein	0	0	0	0,000%	0,000%	0,000%
HTSR_0093	cell division inhibitor	0	0	0	0,000%	0,000%	0,000%
HTSR_0094	histidine triad (HIT) hydrolase	3	3	0	0,027%	0,028%	0,000%
HTSR_0095	isoleucyl-tRNA synthetase	31	28	18	0,281%	0,265%	0,431%
HTSR_0096	ribose-phosphate pyrophosphokinase	9	15	11	0,082%	0,142%	0,263%
HTSR_0097	phosphohexomutase (phosphoglucomutase	8	19	22	0,073%	0,179%	0,527%
HTSR_0098	iron (metal) dependent repressor, DtxR family	7	8	5	0,064%	0,076%	0,120%
HTSR_0099	hypothetical protein	0	0	0	0,000%	0,000%	0,000%
HTSR_0100	hypothetical protein	2	3	3	0,018%	0,028%	0,072%
HTSR_0101	hypothetical protein	0	0	0	0,000%	0,000%	0,000%
HTSR_0102	hypothetical protein	0	0	0	0,000%	0,000%	0,000%

HTSR_0103	PilT domain-containing protein	0	0	0	0,000%	0,000%	0,000%
HTSR_0104	hypothetical protein	0	0	0	0,000%	0,000%	0,000%
HTSR_0106	hypothetical protein	0	0	0	0,000%	0,000%	0,000%
HTSR_0107	DEAD/DEAH box helicase domain protein / Archaea-specific Superfamily II helicase MJ1401	0	9	17	0,000%	0,085%	0,407%
HTSR_0108	cupin domain-containing protein	4	0	3	0,036%	0,000%	0,072%
HTSR_0109	Zn-dependent protease	4	12	2	0,036%	0,113%	0,048%
HTSR_0110	PAS/PAC sensor signal transduction histidine kinase	5	17	7	0,045%	0,161%	0,168%
HTSR_0112	helicase	0	0	0	0,000%	0,000%	0,000%
HTSR_0113	CopG family transcriptional regulator	2	0	4	0,018%	0,000%	0,096%
HTSR_0114	DNA-binding protein	17	5	8	0,154%	0,047%	0,191%
HTSR_0115	replication factor A	24	19	16	0,218%	0,179%	0,383%
HTSR_0116	hypothetical protein	0	0	1	0,000%	0,000%	0,024%
HTSR_0117	DNA binding protein, Tfx family	11	7	3	0,100%	0,066%	0,072%
HTSR_0118	TRAM domain-containing protein	1	1	0	0,009%	0,009%	0,000%
HTSR_0119	iron ABC transporter permease	0	0	0	0,000%	0,000%	0,000%
HTSR_0120	iron ABC transporter substrate-binding protein	4	0	9	0,036%	0,000%	0,215%
HTSR_0121	ABC transporter ATP-binding protein	0	0	0	0,000%	0,000%	0,000%
HTSR_0122	DNA-binding TFAR19-related protein	8	3	5	0,073%	0,028%	0,120%
HTSR_0123	histidyl-tRNA synthetase	14	14	15	0,127%	0,132%	0,359%
HTSR_0124	tRNA pseudouridine synthase A	0	0	2	0,000%	0,000%	0,048%
HTSR_0125	oligoendopeptidase F	15	21	10	0,136%	0,198%	0,239%
HTSR_0126	acetyltransferase, N-acetylglutamate synthase	0	0	0	0,000%	0,000%	0,000%
HTSR_0127	protoporphyrinogen oxidase	12	14	13	0,109%	0,132%	0,311%
HTSR_0128	radical SAM protein	3	9	6	0,027%	0,085%	0,144%
HTSR_0129	glutamyl-tRNA reductase	4	1	19	0,036%	0,009%	0,455%
HTSR_0130	proteasome-activating nucleotidase	10	6	20	0,091%	0,057%	0,479%
HTSR_0131	pyruvoyl-dependent arginine decarboxylase	0	1	0	0,000%	0,009%	0,000%
HTSR_0132	hypothetical protein	1	0	0	0,009%	0,000%	0,000%
HTSR_0133	hypothetical protein	0	0	0	0,000%	0,000%	0,000%
HTSR_0134	translation initiation factor IF-2	17	23	20	0,154%	0,217%	0,479%
HTSR_0135	PRC-barrel domain protein	5	8	4	0,045%	0,076%	0,096%
HTSR_0136	nucleic acid-binding protein	6	3	3	0,054%	0,028%	0,072%
HTSR_0137	hypothetical protein	0	0	0	0,000%	0,000%	0,000%
HTSR_0138	Glucose-6-phosphate isomerase	11	15	9	0,100%	0,142%	0,215%
HTSR_0139	hypothetical protein	0	0	0	0,000%	0,000%	0,000%
HTSR_0140	hypothetical protein	0	0	0	0,000%	0,000%	0,000%
HTSR_0141	preprotein translocase subunit SecF	2	2	3	0,018%	0,019%	0,072%
HTSR_0142	preprotein translocase subunit SecD	15	16	7	0,136%	0,151%	0,168%
HTSR_0143	ribonuclease HII	0	0	0	0,000%	0,000%	0,000%
HTSR_0144	pseudouridylate synthase	5	8	6	0,045%	0,076%	0,144%
HTSR_0145	hypothetical protein	2	3	0	0,018%	0,028%	0,000%
HTSR_0146	tRNA (pseudouridine-N1)-methyltransferase	2	9	9	0,018%	0,085%	0,215%
HTSR_0148	hypothetical protein	0	0	3	0,000%	0,000%	0,072%
HTSR_0149	hypothetical protein	0	0	1	0,000%	0,000%	0,024%
HTSR_0150	PilT protein domain-containing protein	0	0	0	0,000%	0,000%	0,000%
HTSR_0151	hypothetical protein	0	0	0	0,000%	0,000%	0,000%
HTSR_0152	hypothetical protein	0	0	2	0,000%	0,000%	0,048%
HTSR_0153	transposase, IS200 like protein	0	0	0	0,000%	0,000%	0,000%
HTSR_0154	hypothetical protein	0	0	0	0,000%	0,000%	0,000%
HTSR_0155	IS1341-type transposase	0	0	0	0,000%	0,000%	0,000%
HTSR_0156	DNA-binding protein	0	0	0	0,000%	0,000%	0,000%
HTSR_0157	membrane associated metalloprotease	0	0	1	0,000%	0,000%	0,024%
HTSR_0158	thiamine-monophosphate kinase	1	2	3	0,009%	0,019%	0,072%
HTSR_0159	30S ribosomal protein S19e	13	11	12	0,118%	0,104%	0,287%
HTSR_0160	adenosylhomocysteinase	14	26	11	0,127%	0,246%	0,263%
HTSR_0161	5-methylthioadenosine/S-adenosylhomocysteine deaminase	11	11	13	0,100%	0,104%	0,311%
HTSR_0162	ATP phosphoribosyltransferase	16	21	16	0,145%	0,198%	0,383%
HTSR_0163	TATA-box binding family protein	5	12	8	0,045%	0,113%	0,191%
HTSR_0164	RNA methylase	1	6	17	0,009%	0,057%	0,407%
HTSR_0165	replication factor C small subunit 2	4	3	7	0,036%	0,028%	0,168%
HTSR_0166	ribonuclease Z	10	15	11	0,091%	0,142%	0,263%
HTSR_0167	iron-sulfur cluster-binding protein FixX	2	1	1	0,018%	0,009%	0,024%
HTSR_0168	FAD dependent oxidoreductase	15	45	21	0,136%	0,425%	0,503%
HTSR_0169	electron transfer flavoprotein subunit alpha	14	32	12	0,127%	0,302%	0,287%
HTSR_0170	electron transfer flavoprotein subunit beta	5	14	5	0,045%	0,132%	0,120%
HTSR_0171	hypothetical protein	0	3	7	0,000%	0,028%	0,168%
HTSR_0172	tyrosyl-tRNA synthetase	8	10	13	0,073%	0,094%	0,311%
HTSR_0173	arsenate reductase (glutaredoxin) ArsC	0	0	0	0,000%	0,000%	0,000%
HTSR_0174	translation initiation factor eIF-1A	2	1	1	0,018%	0,009%	0,024%
HTSR_0175	serine/threonine protein kinase	2	1	5	0,018%	0,009%	0,120%
HTSR_0176	RNA-processing protein	9	7	6	0,082%	0,066%	0,144%
HTSR_0177	thermosome subunit alpha	47	29	36	0,426%	0,274%	0,862%
HTSR_0178	ornithine cyclodeaminase	11	17	9	0,100%	0,161%	0,215%
HTSR_0179	leucyl-tRNA synthetase	50	63	43	0,454%	0,595%	1,029%
HTSR_0180	hsp20-type chaperone	10	18	5	0,091%	0,170%	0,120%
HTSR_0181	Prephenate dehydratase	8	12	8	0,073%	0,113%	0,191%
HTSR_0182	dihydrolipoamide dehydrogenase	23	19	14	0,209%	0,179%	0,335%
HTSR_0183	DEAD/DEAH box helicase	5	9	30	0,045%	0,085%	0,718%
HTSR_0184	DNA topoisomerase I	0	0	0	0,000%	0,000%	0,000%
HTSR_0185	tRNA-intron endonuclease	4	17	22	0,036%	0,161%	0,527%
HTSR_0186	tryptophanyl-tRNA synthetase	6	12	6	0,054%	0,113%	0,144%
HTSR_0187	phenylalanyl-tRNA synthetase subunit alpha	13	14	14	0,118%	0,132%	0,335%
HTSR_0188	phenylalanyl-tRNA synthetase subunit beta	14	28	21	0,127%	0,265%	0,503%
HTSR_0189	dihydroorotate dehydrogenase	13	10	9	0,118%	0,094%	0,215%
HTSR_0190	hypothetical protein	0	1	0	0,000%	0,009%	0,000%
HTSR_0191	hypothetical protein	0	0	0	0,000%	0,000%	0,000%
HTSR_0192	Non-histone chromosomal MC1 family protein	5	7	5	0,045%	0,066%	0,120%
HTSR_0197	hypothetical protein	0	0	0	0,000%	0,000%	0,000%
HTSR_0198	C4-dicarboxylate ABC transporter substrate-binding protein	9	14	11	0,082%	0,132%	0,263%
HTSR_0199	C4-dicarboxylate ABC transporter	1	0	3	0,009%	0,000%	0,072%
HTSR_0200	C4-dicarboxylate ABC transporter permease	7	8	5	0,064%	0,076%	0,120%
HTSR_0201	hypothetical protein	0	0	7	0,000%	0,000%	0,168%
HTSR_0203	hypothetical protein	0	0	0	0,000%	0,000%	0,000%
HTSR_0204	hypothetical protein	4	2	0	0,036%	0,019%	0,000%
HTSR_0205	hypothetical protein	0	1	1	0,000%	0,009%	0,024%
HTSR_0206	chromosome segregation protein SMC	24	24	28	0,218%	0,227%	0,670%
HTSR_0207	hypothetical protein	7	3	2	0,064%	0,028%	0,048%
HTSR_0208	orc1/cdc6 family replication initiation protein	0	0	3	0,000%	0,000%	0,072%
HTSR_0209	amidase	1	4	7	0,009%	0,038%	0,168%
HTSR_0210	spermidine/putrescine-binding periplasmic protein potD	5	5	5	0,045%	0,047%	0,120%
HTSR_0211	spermidine/putrescine ABC transporter ATPase component potA	2	12	3	0,018%	0,113%	0,072%
HTSR_0212	spermidine/putrescine ABC transporter permease component potB	0	0	0	0,000%	0,000%	0,000%
HTSR_0213	spermidine/putrescine ABC transporter permease component potC	1	1	0	0,009%	0,009%	0,000%
HTSR_0214	short-chain dehydrogenase/reductase SDR	3	8	3	0,027%	0,076%	0,072%
HTSR_0215	calcium-binding protein	0	0	1	0,000%	0,000%	0,024%

HTSR_0216	hypothetical protein / carboxymethylenebutenolidase-related protein	0	3	3	0,000%	0,028%	0,072%
HTSR_0217	hypothetical protein	0	0	0	0,000%	0,000%	0,000%
HTSR_0218	hypothetical protein	0	0	0	0,000%	0,000%	0,000%
HTSR_0219	chromosome segregation protein SMC	26	27	19	0,236%	0,255%	0,455%
HTSR_0220	hypothetical protein	6	5	4	0,054%	0,047%	0,096%
HTSR_0221	IclR family transcriptional regulator	5	0	13	0,045%	0,000%	0,311%
HTSR_0222	aldehyde dehydrogenase	3	3	7	0,027%	0,028%	0,168%
HTSR_0223	short-chain dehydrogenase/reductase SDR	17	13	8	0,154%	0,123%	0,191%
HTSR_0224	NADH:flavin oxidoreductase/NADH oxidase	3	5	3	0,027%	0,047%	0,072%
HTSR_0225	electron transfer flavoprotein subunit alpha	1	0	0	0,009%	0,000%	0,000%
HTSR_0226	FAD dependent oxidoreductase	0	0	6	0,000%	0,000%	0,144%
HTSR_0227	4Fe-4S ferredoxin	0	0	0	0,000%	0,000%	0,000%
HTSR_0228	electron transfer flavoprotein alpha/beta- subunit	0	0	0	0,000%	0,000%	0,000%
HTSR_0229	short-chain dehydrogenase/reductase SDR	0	0	2	0,000%	0,000%	0,048%
HTSR_0230	NADH:flavin oxidoreductase/NADH oxidase	7	19	17	0,064%	0,179%	0,407%
HTSR_0231	mycofactocin system protein MftB	0	0	0	0,000%	0,000%	0,000%
HTSR_0232	mycofactocin radical SAM maturase	0	0	0	0,000%	0,000%	0,000%
HTSR_0233	FkbM family methyltransferase	0	0	0	0,000%	0,000%	0,000%
HTSR_0234	BCCT transporter	0	0	0	0,000%	0,000%	0,000%
HTSR_0235	creatinine amidohydrolase	1	0	0	0,009%	0,000%	0,000%
HTSR_0236	hypothetical protein	2	2	0	0,018%	0,019%	0,000%
HTSR_0237	serine O-acetyltransferase	3	4	6	0,027%	0,038%	0,144%
HTSR_0238	cytochrome oxidase subunit I	0	0	0	0,000%	0,000%	0,000%
HTSR_0239	hypothetical protein	0	0	0	0,000%	0,000%	0,000%
HTSR_0240	ATPase	0	0	0	0,000%	0,000%	0,000%
HTSR_0241	hypothetical protein	0	0	0	0,000%	0,000%	0,000%
HTSR_0242	Protein-L-isoaspartate(D-aspartate) O-methyltransferase	0	0	4	0,000%	0,000%	0,096%
HTSR_0243	hypothetical protein	11	8	9	0,100%	0,076%	0,215%
HTSR_0244	leucyl aminopeptidase (aminopeptidase T)	11	10	9	0,100%	0,094%	0,215%
HTSR_0245	hypothetical protein	0	0	0	0,000%	0,000%	0,000%
HTSR_0246	glyceraldehyde-3-phosphate dehydrogenase	21	26	16	0,191%	0,246%	0,383%
HTSR_0247	heat shock protein Hsp20	6	5	2	0,054%	0,047%	0,048%
HTSR_0248	RimK domain protein ATP-grasp / Coenzyme gamma-F420-2:L-glutamate ligase	0	0	5	0,000%	0,000%	0,120%
HTSR_0249	50S ribosomal protein L10e	5	9	14	0,045%	0,085%	0,335%
HTSR_0250	hypothetical protein	10	15	4	0,091%	0,142%	0,096%
HTSR_0251	RecA-superfamily ATPase	1	6	10	0,009%	0,057%	0,239%
HTSR_0252	hypothetical protein	0	0	0	0,000%	0,000%	0,000%
HTSR_0253	SNF family Na+-dependent transporter	1	2	2	0,009%	0,019%	0,048%
HTSR_0254	DNA methylase N-4/N-6 domain protein	0	7	7	0,000%	0,066%	0,168%
HTSR_0255	TrkA-N domain protein	3	14	11	0,027%	0,132%	0,263%
HTSR_0256	phosphatidylethanolamine-binding protein	5	5	0	0,045%	0,047%	0,000%
HTSR_0257	carbonic anhydrase	3	2	3	0,027%	0,019%	0,072%
HTSR_0258	hypothetical protein	2	2	1	0,018%	0,019%	0,024%
HTSR_0259	hypothetical protein	0	0	0	0,000%	0,000%	0,000%
HTSR_0260	hypothetical protein	0	0	0	0,000%	0,000%	0,000%
HTSR_0261	hypothetical protein	0	0	0	0,000%	0,000%	0,000%
HTSR_0262	ArsR family transcriptional regulator	0	0	0	0,000%	0,000%	0,000%
HTSR_0263	hypothetical protein	0	0	0	0,000%	0,000%	0,000%
HTSR_0264	peptidase S49	0	0	6	0,000%	0,000%	0,144%
HTSR_0265	hypothetical protein	0	0	0	0,000%	0,000%	0,000%
HTSR_0266	hypothetical protein	0	0	0	0,000%	0,000%	0,000%
HTSR_0268	AIR synthase related protein domain protein	3	5	5	0,027%	0,047%	0,120%
HTSR_0269	DNA mismatch repair protein MutS	3	14	19	0,027%	0,132%	0,455%
HTSR_0270	DNA mismatch repair protein MutL	2	5	9	0,018%	0,047%	0,215%
HTSR_0271	FMN-binding domain protein	6	14	8	0,054%	0,132%	0,191%
HTSR_0272	UBA/THIF-type NAD/FAD binding protein	8	9	5	0,073%	0,085%	0,120%
HTSR_0273	cold-shock protein	6	2	2	0,054%	0,019%	0,048%
HTSR_0274	oxidoreductase (glycolate oxidase iron-sulfur subunit)	0	11	6	0,000%	0,104%	0,144%
HTSR_0275	CopG family transcriptional regulator	4	1	1	0,036%	0,009%	0,024%
HTSR_0276	hypothetical protein	0	0	0	0,000%	0,000%	0,000%
HTSR_0277	branched-chain amino acid aminotransferase	36	34	29	0,327%	0,321%	0,694%
HTSR_0278	hypothetical protein	1	4	1	0,009%	0,038%	0,024%
HTSR_0279	hypothetical protein	0	0	0	0,000%	0,000%	0,000%
HTSR_0280	orotate phosphoribosyltransferase	5	7	6	0,045%	0,066%	0,144%
HTSR_0281	adenine phosphoribosyltransferase	10	8	8	0,091%	0,076%	0,191%
HTSR_0282	xanthine/uracil permease family transport protein	1	1	1	0,009%	0,009%	0,024%
HTSR_0283	hypothetical protein	1	2	7	0,009%	0,019%	0,168%
HTSR_0284	threonyl-tRNA synthetase	31	38	29	0,281%	0,359%	0,694%
HTSR_0285	transcriptional regulator / Phage shock protein A	23	17	19	0,209%	0,161%	0,455%
HTSR_0286	CTP synthase	15	16	16	0,136%	0,151%	0,383%
HTSR_0287	GMP synthase subunit B	16	24	24	0,145%	0,227%	0,574%
HTSR_0288	hypothetical protein	4	4	4	0,036%	0,038%	0,096%
HTSR_0289	hypothetical protein / CRISPR repeat RNA endoribonuclease Cas6	8	21	9	0,073%	0,198%	0,215%
HTSR_0290	glyoxalase/bleomycin resistance protein/dioxygenase	15	12	9	0,136%	0,113%	0,215%
HTSR_0291	molybdenum cofactor biosynthesis protein MoaB	4	5	1	0,036%	0,047%	0,024%
HTSR_0292	isopentenyl-diphosphate delta-isomerase	4	7	5	0,036%	0,066%	0,120%
HTSR_0293	AsnC family transcriptional regulator	0	0	3	0,000%	0,000%	0,072%
HTSR_0294	carbamoyl-phosphate synthase, small subunit	7	0	5	0,064%	0,000%	0,120%
HTSR_0295	hypothetical protein	12	14	3	0,109%	0,132%	0,072%
HTSR_0296	carbamoyl-phosphate synthase large subunit	36	34	21	0,327%	0,321%	0,503%
HTSR_0297	hypothetical protein	0	0	0	0,000%	0,000%	0,000%
HTSR_0298	aminodeoxychorismate lyase	12	19	13	0,109%	0,179%	0,311%
HTSR_0299	para-aminobenzoate synthetase component II	4	3	1	0,036%	0,028%	0,024%
HTSR_0300	para-aminobenzoate synthetase component I	15	22	16	0,136%	0,208%	0,383%
HTSR_0301	hypothetical protein	3	5	5	0,027%	0,047%	0,120%
HTSR_0302	hypothetical protein	3	4	5	0,027%	0,038%	0,120%
HTSR_0303	NAD(P)H-nitrite reductase	40	41	29	0,363%	0,387%	0,694%
HTSR_0304	metallo-beta-lactamase superfamily protein	1	2	1	0,009%	0,019%	0,024%
HTSR_0305	zinc finger CDGSH-type domain-containing protein	1	3	0	0,009%	0,028%	0,000%
HTSR_0306	protoporphyrinogen oxidase	6	8	10	0,054%	0,076%	0,239%
HTSR_0307	CBS-domain-containing membrane protein	28	2	29	0,254%	0,019%	0,694%
HTSR_0308	hypothetical protein	1	1	0	0,009%	0,009%	0,000%
HTSR_0309	AbrB family transcriptional regulator	0	0	1	0,000%	0,000%	0,024%
HTSR_0310	IS1341-type transposase	0	0	4	0,000%	0,000%	0,096%
HTSR_0311	DNA repair and recombination protein RadB	0	0	15	0,000%	0,000%	0,359%
HTSR_0312	cell division cycle protein CdcH	43	47	27	0,390%	0,444%	0,646%
HTSR_0313	30S ribosomal protein S8e	14	8	14	0,127%	0,076%	0,335%
HTSR_0314	hypothetical protein	0	0	0	0,000%	0,000%	0,000%
HTSR_0315	hypothetical protein	2	5	3	0,018%	0,047%	0,072%
HTSR_0316	orotidine 5'-phosphate decarboxylase	4	7	4	0,036%	0,066%	0,096%
HTSR_0317	IS1341-type transposase	0	0	2	0,000%	0,000%	0,048%
HTSR_0318	GTP-binding protein	1	0	6	0,009%	0,000%	0,144%
HTSR_0319	Phytoene synthase	1	0	4	0,009%	0,000%	0,096%
HTSR_0320	carotene biosynthesis associated membrane protein	1	0	0	0,009%	0,000%	0,000%
HTSR_0321	UbiA prenyltransferase Lycopene elongase (EC 2.5.1.-)	0	1	1	0,000%	0,009%	0,024%

HTSR_0322	phytoene desaturase	13	30	12	0,118%	0,283%	0,287%
HTSR_0323	methenyltetrahydromethanopterin cyclohydrolase	9	16	9	0,082%	0,151%	0,215%
HTSR_0324	hypothetical protein	6	6	3	0,054%	0,057%	0,072%
HTSR_0325	hypothetical protein	1	0	3	0,009%	0,000%	0,072%
HTSR_0326	hypothetical protein	2	5	0	0,018%	0,047%	0,000%
HTSR_0327	hypothetical protein	1	1	1	0,009%	0,009%	0,024%
HTSR_0329	hypothetical protein	1	2	1	0,009%	0,019%	0,024%
HTSR_0330	hypothetical protein	1	3	9	0,009%	0,028%	0,215%
HTSR_0331	50S ribosomal protein L3	14	10	18	0,127%	0,094%	0,431%
HTSR_0332	50S ribosomal protein L4	19	16	19	0,172%	0,151%	0,455%
HTSR_0333	50S ribosomal protein L23	6	2	2	0,054%	0,019%	0,048%
HTSR_0334	50S ribosomal protein L2	13	14	21	0,118%	0,132%	0,503%
HTSR_0335	30S ribosomal protein S19	8	6	12	0,073%	0,057%	0,287%
HTSR_0336	50S ribosomal protein L22	9	3	4	0,082%	0,028%	0,096%
HTSR_0337	30S ribosomal protein S3	19	11	16	0,172%	0,104%	0,383%
HTSR_0338	50S ribosomal protein L29	4	3	5	0,036%	0,028%	0,120%
HTSR_0339	Ribonuclease P, Rpp29	0	0	2	0,000%	0,000%	0,048%
HTSR_0340	30S ribosomal protein S17	6	5	7	0,054%	0,047%	0,168%
HTSR_0341	50S ribosomal protein L14	10	14	20	0,091%	0,132%	0,479%
HTSR_0342	50S ribosomal protein L24	9	5	8	0,082%	0,047%	0,191%
HTSR_0343	30S ribosomal protein S4e	17	6	9	0,154%	0,057%	0,215%
HTSR_0344	50S ribosomal protein L5	6	9	4	0,054%	0,085%	0,096%
HTSR_0345	30S ribosomal protein S14	2	3	5	0,018%	0,028%	0,120%
HTSR_0346	30S ribosomal protein S8	9	5	10	0,082%	0,047%	0,239%
HTSR_0347	50S ribosomal protein L6	13	8	9	0,118%	0,076%	0,215%
HTSR_0348	50S ribosomal protein L32e	12	11	14	0,109%	0,104%	0,335%
HTSR_0349	50S ribosomal protein L19e	16	13	15	0,145%	0,123%	0,359%
HTSR_0350	50S ribosomal protein L18	6	9	13	0,054%	0,085%	0,311%
HTSR_0351	30S ribosomal protein S5	12	10	21	0,109%	0,094%	0,503%
HTSR_0352	50S ribosomal protein L30	10	7	5	0,091%	0,066%	0,120%
HTSR_0353	50S ribosomal protein L15	6	5	9	0,054%	0,047%	0,215%
HTSR_0354	preprotein translocase subunit SecY	5	7	7	0,045%	0,066%	0,168%
HTSR_0355	amidohydrolase	3	12	14	0,027%	0,113%	0,335%
HTSR_0356	hypothetical protein	0	1	0	0,000%	0,009%	0,000%
HTSR_0357	potassium transport system protein TrkH	1	4	0	0,009%	0,038%	0,000%
HTSR_0358	TrkA-N domain protein	6	4	1	0,054%	0,038%	0,024%
HTSR_0359	hypothetical protein	0	0	0	0,000%	0,000%	0,000%
HTSR_0360	AsnC family transcriptional regulator	4	7	6	0,036%	0,066%	0,144%
HTSR_0361	universal stress protein UspA	9	10	4	0,082%	0,094%	0,096%
HTSR_0362	phosphate permease	8	7	9	0,073%	0,066%	0,215%
HTSR_0363	DNA repair helicase	4	2	10	0,036%	0,019%	0,239%
HTSR_0364	oxidoreductase	1	13	16	0,009%	0,123%	0,383%
HTSR_0365	hypothetical protein	0	0	0	0,000%	0,000%	0,000%
HTSR_0366	hypothetical protein	0	0	0	0,000%	0,000%	0,000%
HTSR_0367	TrkA-C domain-containing protein	2	1	1	0,018%	0,009%	0,024%
HTSR_0368	sodium/hydrogen exchanger	0	1	1	0,000%	0,009%	0,024%
HTSR_0369	major facilitator superfamily MFS_1	1	1	0	0,009%	0,009%	0,000%
HTSR_0370	hypothetical protein	0	1	0	0,000%	0,009%	0,000%
HTSR_0371	putative transporter component	0	1	0	0,000%	0,009%	0,000%
HTSR_0372	fused rhodanese domain-containing protein/hydrolase	12	8	7	0,109%	0,076%	0,168%
HTSR_0373	hypothetical protein	1	1	0	0,009%	0,009%	0,000%
HTSR_0374	ABC transporter	0	2	3	0,000%	0,019%	0,072%
HTSR_0375	hypothetical protein	0	6	2	0,000%	0,057%	0,048%
HTSR_0376	metal-dependent hydrolase	0	0	0	0,000%	0,000%	0,000%
HTSR_0377	dipeptidase	3	14	1	0,027%	0,132%	0,024%
HTSR_0378	hypothetical protein	0	0	1	0,000%	0,000%	0,024%
HTSR_0379	homoserine kinase	2	2	5	0,018%	0,019%	0,120%
HTSR_0380	3-isopropylmalate dehydrogenase	10	16	8	0,091%	0,151%	0,191%
HTSR_0381	3-isopropylmalate dehydratase small subunit	13	4	7	0,118%	0,038%	0,168%
HTSR_0382	3-isopropylmalate dehydratase large subunit	14	0	14	0,127%	0,000%	0,335%
HTSR_0383	ketol-acid reductoisomerase	19	27	12	0,172%	0,255%	0,287%
HTSR_0384	acetolactate synthase small subunit	9	18	12	0,082%	0,170%	0,287%
HTSR_0385	acetolactate synthase large subunit	6	4	7	0,054%	0,038%	0,168%
HTSR_0386	pyruvate carboxyltransferase	2	0	5	0,018%	0,000%	0,120%
HTSR_0387	hypothetical protein	0	0	1	0,000%	0,000%	0,024%
HTSR_0388	hypothetical protein	0	0	0	0,000%	0,000%	0,000%
HTSR_0389	ATPase AAA / Nitric oxide reductase activation protein NorQ	0	0	4	0,000%	0,000%	0,096%
HTSR_0390	von Willebrand factor type A	0	0	3	0,000%	0,000%	0,072%
HTSR_0391	signal-transducing histidine kinase	6	12	11	0,054%	0,113%	0,263%
HTSR_0392	PAS domain s-box	0	5	6	0,000%	0,047%	0,144%
HTSR_0393	hypothetical protein	4	1	4	0,036%	0,009%	0,096%
HTSR_0394	Na+/solute symporter	6	1	1	0,054%	0,009%	0,024%
HTSR_0395	hypothetical protein	0	0	0	0,000%	0,000%	0,000%
HTSR_0396	DNA primase	9	11	5	0,082%	0,104%	0,120%
HTSR_0397	conserved hypothetical membrane protein (DUF92)	1	1	0	0,009%	0,009%	0,000%
HTSR_0398	undecaprenyl diphosphate synthase	3	5	6	0,027%	0,047%	0,144%
HTSR_0399	cell division protein FtsZ	0	0	0	0,000%	0,000%	0,000%
HTSR_0400	DNA-directed DNA polymerase	0	0	0	0,000%	0,000%	0,000%
HTSR_0401	hypothetical protein	0	0	0	0,000%	0,000%	0,000%
HTSR_0402	hypothetical protein	0	0	0	0,000%	0,000%	0,000%
HTSR_0403	putative metal-dependent membrane protease	0	0	0	0,000%	0,000%	0,000%
HTSR_0404	orc1/cdc6 family replication initiation protein	0	0	3	0,000%	0,000%	0,072%
HTSR_0405	glutathione S-transferase	7	5	10	0,064%	0,047%	0,239%
HTSR_0406	hypothetical protein	5	3	3	0,045%	0,028%	0,072%
HTSR_0407	undecaprenyl diphosphate synthase	1	0	1	0,009%	0,000%	0,024%
HTSR_0408	hypothetical protein	3	2	4	0,027%	0,019%	0,096%
HTSR_0409	pterin-4-alpha-carbinolamine dehydratase	1	2	1	0,009%	0,019%	0,024%
HTSR_0410	hypothetical protein	0	0	0	0,000%	0,000%	0,000%
HTSR_0411	molybdenum cofactor biosynthesis protein MoeA	3	7	5	0,027%	0,066%	0,120%
HTSR_0412	heat shock protein Hsp20	3	5	4	0,027%	0,047%	0,096%
HTSR_0413	membrane protein	16	38	23	0,145%	0,359%	0,551%
HTSR_0414	aspartate transaminase	14	10	9	0,127%	0,094%	0,215%
HTSR_0415	translation initiation factor eIF-5A	11	7	6	0,100%	0,066%	0,144%
HTSR_0416	agmatinase	0	1	0	0,000%	0,009%	0,000%
HTSR_0417	dinuclear metal center protein, YbgI/SA1388 family	2	8	2	0,018%	0,076%	0,048%
HTSR_0418	carboxymuconolactone decarboxylase	1	3	0	0,009%	0,028%	0,000%
HTSR_0419	deoxyhypusine synthase	9	5	5	0,082%	0,047%	0,120%
HTSR_0420	hypothetical protein	2	0	0	0,018%	0,000%	0,000%
HTSR_0421	bacterio-opsin activator HTH domain-containing protein	0	0	1	0,000%	0,000%	0,024%
HTSR_0422	4Fe-4S ferredoxin iron-sulfur binding domain protein	0	0	0	0,000%	0,000%	0,000%
HTSR_0423	dimethylsulfoxide reductase	1	5	1	0,009%	0,047%	0,024%
HTSR_0424	molybdopterin oxidoreductase	0	0	0	0,000%	0,000%	0,000%
HTSR_0425	polysulfide reductase NrfD	0	0	0	0,000%	0,000%	0,000%
HTSR_0426	chaperone protein TorD	0	0	0	0,000%	0,000%	0,000%
HTSR_0427	ion transporter	0	3	0	0,000%	0,028%	0,000%

HTSR_0428	zinc/iron permease	0	0	0	0,000%	0,000%	0,000%
HTSR_0429	hypothetical protein / Magnesium and cobalt efflux protein CorC	7	12	5	0,064%	0,113%	0,120%
HTSR_0430	hypothetical protein	0	0	0	0,000%	0,000%	0,000%
HTSR_0431	hypothetical protein	2	4	0	0,018%	0,038%	0,000%
HTSR_0432	cationic amino acid transporter	0	2	0	0,000%	0,019%	0,000%
HTSR_0433	universal stress protein UspA	0	3	1	0,000%	0,028%	0,024%
HTSR_0434	malate dehydrogenase	26	29	18	0,236%	0,274%	0,431%
HTSR_0435	DMT family permease	0	3	8	0,000%	0,028%	0,191%
HTSR_0436	pyridoxamine 5'-phosphate oxidase	5	1	2	0,045%	0,009%	0,048%
HTSR_0437	translation initiation factor aIF-1A	3	1	3	0,027%	0,009%	0,072%
HTSR_0438	FAD dependent oxidoreductase	16	28	12	0,145%	0,265%	0,287%
HTSR_0439	HAD-superfamily hydrolase	1	0	7	0,009%	0,000%	0,168%
HTSR_0440	DNA-directed RNA-polymerase subunit M	0	0	0	0,000%	0,000%	0,000%
HTSR_0441	hypothetical protein	2	1	0	0,018%	0,009%	0,000%
HTSR_0442	hypothetical protein / Vng1740c	6	2	5	0,054%	0,019%	0,120%
HTSR_0443	RecA-superfamily ATPase	1	9	5	0,009%	0,085%	0,120%
HTSR_0444	GYD family protein	2	2	2	0,018%	0,019%	0,048%
HTSR_0445	phage PhiH1 repressor protein	8	5	6	0,073%	0,047%	0,144%
HTSR_0446	formate/nitrite transporter	13	21	14	0,118%	0,198%	0,335%
HTSR_0447	3-oxoacyl-ACP reductase	3	10	0	0,027%	0,094%	0,000%
HTSR_0448	nicotinate-nucleotide--dimethylbenzimidazole phosphoribosyltransferase	7	0	5	0,064%	0,000%	0,120%
HTSR_0449	methyl-accepting chemotaxis sensory transducer	20	27	15	0,181%	0,255%	0,359%
HTSR_0450	chemotaxis protein CheW	0	0	0	0,000%	0,000%	0,000%
HTSR_0451	methyl-accepting chemotaxis sensory transducer with Pas/Pac sensor	3	1	1	0,027%	0,009%	0,024%
HTSR_0452	PAS/PAC sensor signal transduction histidine kinase	0	0	1	0,000%	0,000%	0,024%
HTSR_0453	hypothetical protein	0	0	2	0,000%	0,000%	0,048%
HTSR_0454	sodium-dependent phosphate transporter	0	0	0	0,000%	0,000%	0,000%
HTSR_0456	tRNA pseudouridine synthase B	4	5	6	0,036%	0,047%	0,144%
HTSR_0457	cytidylate kinase	10	12	5	0,091%	0,113%	0,120%
HTSR_0458	HTR-like protein	10	9	7	0,091%	0,085%	0,168%
HTSR_0459	adenylate kinase	6	9	4	0,054%	0,085%	0,096%
HTSR_0460	hypothetical protein	6	4	1	0,054%	0,038%	0,024%
HTSR_0461	potassium transporter TrkH	3	0	2	0,027%	0,000%	0,048%
HTSR_0462	potassium transporter TrkA	4	10	9	0,036%	0,094%	0,215%
HTSR_0463	hypothetical protein	1	2	1	0,009%	0,019%	0,024%
HTSR_0464	molecular chaperone Hsp20	0	0	0	0,000%	0,000%	0,000%
HTSR_0465	hypothetical protein	0	0	0	0,000%	0,000%	0,000%
HTSR_0466	hypothetical protein	1	1	1	0,009%	0,009%	0,024%
HTSR_0467	ArsR family transcriptional regulator	0	1	0	0,000%	0,009%	0,000%
HTSR_0468	hypothetical protein	0	0	0	0,000%	0,000%	0,000%
HTSR_0469	signal transduction histidine kinase	0	2	1	0,000%	0,019%	0,024%
HTSR_0470	PAS sensor histidine kinase	1	5	8	0,009%	0,047%	0,191%
HTSR_0471	methyl-accepting chemotaxis sensory transducer with Pas/Pac sensor	28	14	10	0,254%	0,132%	0,239%
HTSR_0472	chemotaxis protein CheW	6	0	5	0,054%	0,000%	0,120%
HTSR_0473	signal-transducing histidine kinase	0	0	0	0,000%	0,000%	0,000%
HTSR_0474	hypothetical protein	0	0	0	0,000%	0,000%	0,000%
HTSR_0475	alcohol dehydrogenase GroES domain protein	0	0	0	0,000%	0,000%	0,000%
HTSR_0477	anthranilate phosphoribosyltransferase	0	0	2	0,000%	0,000%	0,048%
HTSR_0478	phosphoribosylanthranilate isomerase	9	1	1	0,082%	0,009%	0,024%
HTSR_0479	anthranilate synthase, aminase component	11	0	16	0,100%	0,000%	0,383%
HTSR_0480	anthranilate synthase, amidotransferase component	2	0	0	0,018%	0,000%	0,000%
HTSR_0481	ribonucleoside-diphosphate reductase	27	33	57	0,245%	0,312%	1,364%
HTSR_0482	hypothetical protein	0	0	0	0,000%	0,000%	0,000%
HTSR_0483	hypothetical protein	8	11	9	0,073%	0,104%	0,215%
HTSR_0485	multi-sensor signal transduction histidine kinase	0	0	2	0,000%	0,000%	0,048%
HTSR_0486	RDD domain containing protein	0	0	0	0,000%	0,000%	0,000%
HTSR_0487	hypothetical protein	0	0	0	0,000%	0,000%	0,000%
HTSR_0488	oxidoreductase (aldo-keto reductase)	16	16	16	0,145%	0,151%	0,383%
HTSR_0489	GCN5-like N-acetyltransferase	6	6	4	0,054%	0,057%	0,096%
HTSR_0490	DMT(drug/metabolite transporter) superfamily permease	0	0	1	0,000%	0,000%	0,024%
HTSR_0491	inosine-5'-monophosphate dehydrogenase	6	5	2	0,054%	0,047%	0,048%
HTSR_0492	hypothetical protein	1	0	2	0,009%	0,000%	0,048%
HTSR_0493	malate dehydrogenase (oxaloacetate-decarboxylating)(NADP+)	29	35	35	0,263%	0,331%	0,838%
HTSR_0494	ATPase AAA	7	11	14	0,064%	0,104%	0,335%
HTSR_0495	acetyltransferase	4	1	1	0,036%	0,009%	0,024%
HTSR_0496	transcriptional regulator	7	1	11	0,064%	0,009%	0,263%
HTSR_0497	Hydroxymethylglutaryl-CoA synthase	35	54	53	0,318%	0,510%	1,269%
HTSR_0498	PKD domain-containing protein	6	3	4	0,054%	0,028%	0,096%
HTSR_0499	hypothetical protein	9	6	7	0,082%	0,057%	0,168%
HTSR_0500	NAD(P)H-flavin oxidoreductase	3	2	4	0,027%	0,019%	0,096%
HTSR_0501	TatD-related deoxyribonuclease	0	2	1	0,000%	0,019%	0,024%
HTSR_0502	hypothetical protein	2	0	1	0,018%	0,000%	0,024%
HTSR_0503	glycine cleavage system protein T	13	33	6	0,118%	0,312%	0,144%
HTSR_0504	glycine cleavage system protein H	2	3	2	0,018%	0,028%	0,048%
HTSR_0505	glycine dehydrogenase subunit 1	22	31	13	0,200%	0,293%	0,311%
HTSR_0506	glycine dehydrogenase subunit 2	33	34	16	0,299%	0,321%	0,383%
HTSR_0507	HxlR family transcriptional regulator	3	1	3	0,027%	0,009%	0,072%
HTSR_0508	hypothetical protein	2	3	0	0,018%	0,028%	0,000%
HTSR_0509	glutaredoxin	2	4	1	0,018%	0,038%	0,024%
HTSR_0510	ATP:cob(i)alamin adenosyltransferase	2	2	2	0,018%	0,019%	0,048%
HTSR_0512	Appr-1-p processing protein	0	0	0	0,000%	0,000%	0,000%
HTSR_0513	hypothetical protein	0	1	0	0,000%	0,009%	0,000%
HTSR_0514	chaperone protein TorD	0	0	6	0,000%	0,000%	0,144%
HTSR_0515	polysulfide reductase NrfD	0	5	9	0,000%	0,047%	0,215%
HTSR_0516	4Fe-4S ferredoxin iron-sulfur binding domain protein	0	5	18	0,000%	0,047%	0,431%
HTSR_0517	dimethylsulfoxide reductase	1	25	87	0,009%	0,236%	2,082%
HTSR_0518	hypothetical protein	0	0	1	0,000%	0,000%	0,024%
HTSR_0519	hypothetical protein	0	0	0	0,000%	0,000%	0,000%
HTSR_0520	bacterio-opsin activator HTH domain-containing protein	0	0	6	0,000%	0,000%	0,144%
HTSR_0521	major facilitator superfamily MFS_1	0	0	0	0,000%	0,000%	0,000%
HTSR_0522	peptidylprolyl isomerase	4	3	3	0,036%	0,028%	0,072%
HTSR_0523	2-dehydro-3-deoxyglucarate aldolase	1	2	3	0,009%	0,019%	0,072%
HTSR_0525	quinohemoprotein alcohol dehydrogenase	2	1	1	0,018%	0,009%	0,024%
HTSR_0526	hypothetical protein	0	0	0	0,000%	0,000%	0,000%
HTSR_0527	hypothetical protein	1	0	0	0,009%	0,000%	0,000%
HTSR_0528	succinyl-CoA synthetase subunit alpha	6	7	8	0,054%	0,066%	0,191%
HTSR_0529	succinyl-CoA synthetase subunit beta	10	17	13	0,091%	0,161%	0,311%
HTSR_0530	zinc-binding dehydrogenase	3	0	5	0,027%	0,000%	0,120%
HTSR_0531	glucose 1-dehydrogenase	7	7	5	0,064%	0,066%	0,120%
HTSR_0532	universal stress protein UspA	4	4	1	0,036%	0,038%	0,024%
HTSR_0533	nucleoside deaminase (cytosine deaminase, guanine deaminase)	2	9	5	0,018%	0,085%	0,120%
HTSR_0534	HD family metal dependent phosphohydrolase	6	6	14	0,054%	0,057%	0,335%
HTSR_0535	2-phospho-L-lactate transferase	0	0	1	0,000%	0,000%	0,024%
HTSR_0536	tRNA dihydrouridine synthase B-like protein	0	0	0	0,000%	0,000%	0,000%
HTSR_0537	30S ribosomal protein S17e	0	4	7	0,000%	0,038%	0,168%

HTSR_0538	aspartate-semialdehyde dehydrogenase	26	11	15	0,236%	0,104%	0,359%
HTSR_0539	D-3-phosphoglycerate dehydrogenase	9	11	10	0,082%	0,104%	0,239%
HTSR_0540	hypothetical protein	0	0	1	0,000%	0,000%	0,024%
HTSR_0541	hypothetical protein	0	0	0	0,000%	0,000%	0,000%
HTSR_0542	bacterio-opsin activator HTH domain-containing protein	3	4	4	0,027%	0,038%	0,096%
HTSR_0543	hypothetical protein	0	1	1	0,000%	0,009%	0,024%
HTSR_0544	hypothetical protein	9	12	7	0,082%	0,113%	0,168%
HTSR_0545	hypothetical protein	0	0	0	0,000%	0,000%	0,000%
HTSR_0546	hypothetical protein	6	3	11	0,054%	0,028%	0,263%
HTSR_0547	hypothetical protein	0	0	0	0,000%	0,000%	0,000%
HTSR_0548	molybdenum-pterin-binding protein	0	1	2	0,000%	0,009%	0,048%
HTSR_0549	hypothetical protein	0	0	1	0,000%	0,000%	0,024%
HTSR_0550	AzlC family protein	0	0	2	0,000%	0,000%	0,048%
HTSR_0551	hypothetical protein	1	0	0	0,009%	0,000%	0,000%
HTSR_0552	hypothetical protein	1	0	0	0,009%	0,000%	0,000%
HTSR_0553	universal stress protein UspA	0	0	0	0,000%	0,000%	0,000%
HTSR_0554	hypothetical protein	9	0	8	0,082%	0,000%	0,191%
HTSR_0555	histidinol dehydrogenase	13	23	20	0,118%	0,217%	0,479%
HTSR_0556	hypothetical protein	1	2	2	0,009%	0,019%	0,048%
HTSR_0557	hypothetical protein	0	1	0	0,000%	0,009%	0,000%
HTSR_0558	hypothetical protein	1	3	2	0,009%	0,028%	0,048%
HTSR_0559	membrane-bound metal-dependent hydrolase	0	0	0	0,000%	0,000%	0,000%
HTSR_0560	translation initiation factor aIF-2B	15	25	18	0,136%	0,236%	0,431%
HTSR_0561	hypothetical protein	10	21	11	0,091%	0,198%	0,263%
HTSR_0562	hypothetical protein	1	3	0	0,009%	0,028%	0,000%
HTSR_0563	GCN5-like N-acetyltransferase	2	8	14	0,018%	0,076%	0,335%
HTSR_0564	DNA primase small subunit	2	7	7	0,018%	0,066%	0,168%
HTSR_0565	hypothetical protein	2	0	1	0,018%	0,000%	0,024%
HTSR_0566	ATPase AAA	56	71	41	0,508%	0,671%	0,981%
HTSR_0567	hypothetical protein	0	0	0	0,000%	0,000%	0,000%
HTSR_0568	3-methyl-2-oxobutanoate hydroxymethyltransferase	1	6	1	0,009%	0,057%	0,024%
HTSR_0569	hypothetical protein	0	0	0	0,000%	0,000%	0,000%
HTSR_0570	alanyl-tRNA synthetase	15	10	9	0,136%	0,094%	0,215%
HTSR_0571	transcriptional regulator	2	1	2	0,018%	0,009%	0,048%
HTSR_0572	MCP domain-containing signal transducer	28	24	22	0,254%	0,227%	0,527%
HTSR_0573	Na+/Ca2+-antiporter-like protein	0	0	0	0,000%	0,000%	0,000%
HTSR_0574	ATPase, P-type (transporting), HAD superfamily, subfamily IC	0	8	0	0,000%	0,076%	0,000%
HTSR_0575	hypothetical protein	0	0	6	0,000%	0,000%	0,144%
HTSR_0577	transcription initiation factor TFB	0	0	0	0,000%	0,000%	0,000%
HTSR_0578	phosphopantetheine adenylyltransferase	2	0	1	0,018%	0,000%	0,024%
HTSR_0579	TrmB family transcriptional regulator	11	13	9	0,100%	0,123%	0,215%
HTSR_0580	carboxylate-amine ligase/glutamate--cysteine ligase GCS2	5	5	5	0,045%	0,047%	0,120%
HTSR_0581	fibrillarin	1	3	2	0,009%	0,028%	0,048%
HTSR_0582	nucleolar protein-like protein	12	13	15	0,109%	0,123%	0,359%
HTSR_0583	Zn-ribbon RNA-binding protein	0	3	3	0,000%	0,028%	0,072%
HTSR_0584	elongation factor 1-beta	6	7	6	0,054%	0,066%	0,144%
HTSR_0585	hypothetical protein	0	0	0	0,000%	0,000%	0,000%
HTSR_0586	translation initiation factor IF-2 subunit beta	6	7	7	0,054%	0,066%	0,168%
HTSR_0587	hypothetical protein	0	0	1	0,000%	0,000%	0,024%
HTSR_0588	hypothetical protein	1	1	0	0,009%	0,009%	0,000%
HTSR_0589	8-oxoguanine DNA glycosylase	1	1	17	0,009%	0,009%	0,407%
HTSR_0590	yjeF family carbohydrate kinase	11	18	12	0,100%	0,170%	0,287%
HTSR_0591	molybdenum cofactor biosynthesis protein MoaC	4	2	3	0,036%	0,019%	0,072%
HTSR_0592	GTP-binding protein HflX	17	14	23	0,154%	0,132%	0,551%
HTSR_0593	FUN14 family protein	1		1	0,009%	0,000%	0,024%
HTSR_0594	RNA-associated protein	20	14	6	0,181%	0,132%	0,144%
HTSR_0595	proteasome subunit alpha	10	15	22	0,091%	0,142%	0,527%
HTSR_0596	ribonuclease P protein subunit RPP14	2	4	5	0,018%	0,038%	0,120%
HTSR_0597	ribonuclease P protein subunit RPP30	1	0	1	0,009%	0,000%	0,024%
HTSR_0598	hypothetical protein	2	1	1	0,018%	0,009%	0,024%
HTSR_0599	hypothetical protein	0	0	0	0,000%	0,000%	0,000%
HTSR_0600	aspartate carbamoyltransferase regulatory subunit	2	9	3	0,018%	0,085%	0,072%
HTSR_0601	aspartate carbamoyltransferase catalytic subunit	14	29	18	0,127%	0,274%	0,431%
HTSR_0602	cobyrinic acid ac-diamide synthase	0	0	0	0,000%	0,000%	0,000%
HTSR_0603	hypothetical protein	0	0	0	0,000%	0,000%	0,000%
HTSR_0604	peptidylprolyl isomerase	21	18	13	0,191%	0,170%	0,311%
HTSR_0605	NRAMP family Mn2+/Fe2+ transporter	4	3	4	0,036%	0,028%	0,096%
HTSR_0606	adenylate cyclase	3	1	1	0,027%	0,009%	0,024%
HTSR_0607	S-adenosylmethionine synthetase	22	35	26	0,200%	0,331%	0,622%
HTSR_0608	tRNA sulfurtransferase	4	3	13	0,036%	0,028%	0,311%
HTSR_0609	hypothetical protein	0	1	0	0,000%	0,009%	0,000%
HTSR_0610	phosphoribosylamine--glycine ligase	11	28	14	0,100%	0,265%	0,335%
HTSR_0611	XapX domain protein	0	0	0	0,000%	0,000%	0,000%
HTSR_0612	succinate dehydrogenase subunit A	17	26	14	0,154%	0,246%	0,335%
HTSR_0613	succinate dehydrogenase subunit B	1	6	0	0,009%	0,057%	0,000%
HTSR_0614	succinate dehydrogenase subunit D	0	0	0	0,000%	0,000%	0,000%
HTSR_0615	succinate dehydrogenase subunit C (cytochrome b-556)	1	1	1	0,009%	0,009%	0,024%
HTSR_0616	succinylglutamate desuccinylase/aspartoacylase	0	8	7	0,000%	0,076%	0,168%
HTSR_0617	alpha-L-glutamate ligase, RimK family	13	28	11	0,118%	0,265%	0,263%
HTSR_0619	hypothetical protein	0	0	0	0,000%	0,000%	0,000%
HTSR_0620	hypothetical protein	0	0	4	0,000%	0,000%	0,096%
HTSR_0621	integral membrane sensor signal transduction histidine kinase	11	4	4	0,100%	0,038%	0,096%
HTSR_0622	hypothetical protein	2	1	4	0,018%	0,009%	0,096%
HTSR_0623	arsenic resistance protein ArsB	0	0	0	0,000%	0,000%	0,000%
HTSR_0624	ArsR family transcriptional regulator	0	0	0	0,000%	0,000%	0,000%
HTSR_0625	transcriptional regulator	0	0	3	0,000%	0,000%	0,072%
HTSR_0626	hypothetical protein	0	1	0	0,000%	0,009%	0,000%
HTSR_0627	molybdopterin oxidoreductase / Formate dehydrogenase(EC:1.2.1.2)	38	82	17	0,345%	0,775%	0,407%
HTSR_0628	4Fe-4S ferredoxin iron-sulfur binding domain protein	12	22	2	0,109%	0,208%	0,048%
HTSR_0629	polysulfide reductase NrfD	3	6	3	0,027%	0,057%	0,072%
HTSR_0630	chaperone protein TorD	0	3	0	0,000%	0,028%	0,000%
HTSR_0631	CoA-binding domain-containing protein / O-acetylhomoserine sulfhydrylase (EC 2.5.1.49) / O-succinylhomoserine sulfhydry	1	1	1	0,009%	0,009%	0,024%
HTSR_0632	permease	0	0	0	0,000%	0,000%	0,000%
HTSR_0633	cation diffusion facilitator family transporter	0	4	4	0,000%	0,038%	0,096%
HTSR_0634	universal stress protein UspA	3	7	2	0,027%	0,066%	0,048%
HTSR_0635	hypothetical protein	2	1	0	0,018%	0,009%	0,000%
HTSR_0636	formamidase	20	86	1	0,181%	0,812%	0,024%
HTSR_0637	AmiS/UreI transporter	0	0	0	0,000%	0,000%	0,000%
HTSR_0638	CrcB-like protein	0	0	0	0,000%	0,000%	0,000%
HTSR_0639	integral membrane protein/CrcB-like protein	0	0	0	0,000%	0,000%	0,000%
HTSR_0640	thermosome subunit alpha	1	19	2	0,009%	0,179%	0,048%
HTSR_0641	hypothetical protein	0	0	0	0,000%	0,000%	0,000%
HTSR_0642	cobalt ABC transporter ATP-binding protein	9	0	0	0,082%	0,000%	0,000%
HTSR_0643	cobalt ABC transporter permease	3	0	0	0,027%	0,000%	0,000%
HTSR_0644	hypothetical protein	4	0	1	0,036%	0,000%	0,024%

HTSR_0645	cobalt ABC transporter permease	1	2	1	0,009%	0,019%	0,024%
HTSR_0646	iron ABC transporter ATP-binding protein	0	0	0	0,000%	0,000%	0,000%
HTSR_0647	iron ABC transporter permease	0	0	0	0,000%	0,000%	0,000%
HTSR_0648	periplasmic binding protein	0	0	1	0,000%	0,000%	0,024%
HTSR_0649	hydrogenase maturation protein HypF	0	17	0	0,000%	0,161%	0,000%
HTSR_0650	hydrogenase nickel incorporation protein HypB	0	0	0	0,000%	0,000%	0,000%
HTSR_0651	hydrogenase nickel incorporation protein HypA	0	0	0	0,000%	0,000%	0,000%
HTSR_0652	hydrogenase expression/formation protein HypE	0	0	0	0,000%	0,000%	0,000%
HTSR_0653	hydrogenase expression/formation protein HypD	0	0	0	0,000%	0,000%	0,000%
HTSR_0654	hydrogenase expression/formation protein HypC	0	0	0	0,000%	0,000%	0,000%
HTSR_0655	hydrogenase maturation protease	0	0	0	0,000%	0,000%	0,000%
HTSR_0656	prokaryotic cytochrome b561	0	7	0	0,000%	0,066%	0,000%
HTSR_0657	Ni/Fe-hydrogenase large subunit	11	26	7	0,100%	0,246%	0,168%
HTSR_0658	Ni/Fe-hydrogenase small subunit	1	5	2	0,009%	0,047%	0,048%
HTSR_0659	hypothetical protein	1	1	3	0,009%	0,009%	0,072%
HTSR_0660	GMP synthase - glutamine amidotransferase domainprotein	0	4	2	0,000%	0,038%	0,048%
HTSR_0661	hypothetical protein	0	0	1	0,000%	0,000%	0,024%
HTSR_0662	succinylglutamate desuccinylase/aspartoacylase	0	1	10	0,000%	0,009%	0,239%
HTSR_0663	hypothetical protein	0	0	0	0,000%	0,000%	0,000%
HTSR_0665	N-acetyl-gamma-glutamyl-phosphate reductase	33	15	27	0,299%	0,142%	0,646%
HTSR_0666	acetylglutamate kinase	14	7	7	0,127%	0,066%	0,168%
HTSR_0667	acetylornithine aminotransferase	27	16	19	0,245%	0,151%	0,455%
HTSR_0668	acetylornithine deacetylase	8	8	10	0,073%	0,076%	0,239%
HTSR_0669	hypothetical protein	0	0	0	0,000%	0,000%	0,000%
HTSR_0670	hypothetical protein	11	22	20	0,100%	0,208%	0,479%
HTSR_0671	serine hydroxymethyltransferase	62	54	37	0,563%	0,510%	0,886%
HTSR_0672	hypothetical protein	4	10	10	0,036%	0,094%	0,239%
HTSR_0673	PadR family transcriptional regulator	4	4	5	0,036%	0,038%	0,120%
HTSR_0674	Htr-like protein	0	1	0	0,000%	0,009%	0,000%
HTSR_0675	hypothetical protein	0	0	0	0,000%	0,000%	0,000%
HTSR_0676	A/G-specific adenine glycosylase	0	1	0	0,000%	0,009%	0,000%
HTSR_0677	NADPH-dependent FMN reductase	8	5	6	0,073%	0,047%	0,144%
HTSR_0678	thymidine kinase	0	0	3	0,000%	0,000%	0,072%
HTSR_0679	nucleic acid binding OB-fold tRNA/helicase-type	28	29	27	0,254%	0,274%	0,646%
HTSR_0680	histone acetyltransferase, ELP3 family protein	2	6	9	0,018%	0,057%	0,215%
HTSR_0681	fibronectin-binding protein	25	32	36	0,227%	0,302%	0,862%
HTSR_0682	peptide chain release factor pelota	7	3	2	0,064%	0,028%	0,048%
HTSR_0683	beta-lactamase	0	2	4	0,000%	0,019%	0,096%
HTSR_0684	ATP-dependent helicase	2	5	10	0,018%	0,047%	0,239%
HTSR_0685	hypothetical protein	0	3	3	0,000%	0,028%	0,072%
HTSR_0686	hypothetical protein	0	0	0	0,000%	0,000%	0,000%
HTSR_0687	integral membrane protein	1	1	0	0,009%	0,009%	0,000%
HTSR_0688	signal-transducing histidine kinase	11	15	13	0,100%	0,142%	0,311%
HTSR_0689	DNA helicase	3	19	16	0,027%	0,179%	0,383%
HTSR_0690	hypothetical protein	12	7	17	0,109%	0,066%	0,407%
HTSR_0691	TetR family transcriptional regulator	4	3	3	0,036%	0,028%	0,072%
HTSR_0692	hypothetical protein	1	1	3	0,009%	0,009%	0,072%
HTSR_0693	ABC transporter ATP-binding protein	0	0	0	0,000%	0,000%	0,000%
HTSR_0694	ABC transporter permease	0	0	0	0,000%	0,000%	0,000%
HTSR_0695	hypothetical protein	3	2	2	0,027%	0,019%	0,048%
HTSR_0696	endoglucanase	7	0	16	0,064%	0,000%	0,383%
HTSR_0697	small nuclear ribonucleoprotein	0	0	0	0,000%	0,000%	0,000%
HTSR_0698	50S ribosomal protein L37e	0	1	2	0,000%	0,009%	0,048%
HTSR_0699	amidophosphoribosyltransferase	8	17	7	0,073%	0,161%	0,168%
HTSR_0701	acylphosphatase	1	3	1	0,009%	0,028%	0,024%
HTSR_0702	IS1341-type transposase	0	0	2	0,000%	0,000%	0,048%
HTSR_0703	hypothetical protein	0	0	0	0,000%	0,000%	0,000%
HTSR_0704	thioredoxin reductase	5	8	8	0,045%	0,076%	0,191%
HTSR_0705	cyclase family protein	1	0	0	0,009%	0,000%	0,000%
HTSR_0706	hypothetical protein	1	1	1	0,009%	0,009%	0,024%
HTSR_0707	universal stress protein UspA	6	19	1	0,054%	0,179%	0,024%
HTSR_0708	5'-nucleotidase	0	1	1	0,000%	0,009%	0,024%
HTSR_0709	hypothetical protein	5	13	6	0,045%	0,123%	0,144%
HTSR_0710	MscS mechanosensitive ion channel	4	4	2	0,036%	0,038%	0,048%
HTSR_0711	galactoside O-acetyltransferase 3	0	1	1	0,000%	0,009%	0,024%
HTSR_0712	hypothetical protein	0	0	0	0,000%	0,000%	0,000%
HTSR_0713	hypothetical protein	0	0	4	0,000%	0,000%	0,096%
HTSR_0714	sporulation regulator-like protein	0	0	1	0,000%	0,000%	0,024%
HTSR_0715	phosphoglycerate kinase	18	21	15	0,163%	0,198%	0,359%
HTSR_0716	signal transduction protein with CBS domains	5	6	3	0,045%	0,057%	0,072%
HTSR_0717	uridine kinase	2	2	3	0,018%	0,019%	0,072%
HTSR_0718	hypothetical protein	0	2	2	0,000%	0,019%	0,048%
HTSR_0719	DoxX family protein	0	0	0	0,000%	0,000%	0,000%
HTSR_0720	hypothetical protein	0	0	0	0,000%	0,000%	0,000%
HTSR_0722	hypothetical protein	0	0	0	0,000%	0,000%	0,000%
HTSR_0723	hypothetical protein	18	17	20	0,163%	0,161%	0,479%
HTSR_0724	chorismate synthase	19	11	14	0,172%	0,104%	0,335%
HTSR_0725	3-phosphoshikimate 1-carboxyvinyltransferase	5	18	7	0,045%	0,170%	0,168%
HTSR_0726	Xaa-Pro aminopeptidase	8	14	9	0,073%	0,132%	0,215%
HTSR_0727	prephenate dehydrogenase	11	10	4	0,100%	0,094%	0,096%
HTSR_0728	methyltransferase family protein	4	9	14	0,036%	0,085%	0,335%
HTSR_0729	5-nucleotidase	1	5	2	0,009%	0,047%	0,048%
HTSR_0730	hypothetical protein	0	0	0	0,000%	0,000%	0,000%
HTSR_0731	MazF family transcriptional regulator	3	2	1	0,027%	0,019%	0,024%
HTSR_0732	chorismate mutase	0	1	3	0,000%	0,009%	0,072%
HTSR_0733	shikimate kinase	4	4	3	0,036%	0,038%	0,072%
HTSR_0734	hypothetical protein	3	2	2	0,027%	0,019%	0,048%
HTSR_0735	small CPxCG-related zinc finger protein	0	0	0	0,000%	0,000%	0,000%
HTSR_0736	cell division control protein 48	25	27	30	0,227%	0,255%	0,718%
HTSR_0737	HAD-superfamily hydrolase	4	2	2	0,036%	0,019%	0,048%
HTSR_0738	ATP-dependent DNA helicase	2	10	23	0,018%	0,094%	0,551%
HTSR_0739	metallophosphoesterase	0	0	0	0,000%	0,000%	0,000%
HTSR_0740	glycerol dehydrogenase	21	21	17	0,191%	0,198%	0,407%
HTSR_0741	5-amino-6-(5-phosphoribosylamino)uracil reductase	1	2	1	0,009%	0,019%	0,024%
HTSR_0742	hypothetical protein	4	3	2	0,036%	0,028%	0,048%
HTSR_0743	thioredoxin-disulfide reductase	11	4	2	0,100%	0,038%	0,048%
HTSR_0744	hypothetical protein	1	2	2	0,009%	0,019%	0,048%
HTSR_0745	50S ribosomal protein L21e	3	4	4	0,027%	0,038%	0,096%
HTSR_0746	DNA-directed RNA polymerase subunit F	6	6	4	0,054%	0,057%	0,096%
HTSR_0747	adenosine deaminase	16	22	10	0,145%	0,208%	0,239%
HTSR_0748	dimethyladenosine transferase	0	8	11	0,000%	0,076%	0,263%
HTSR_0749	methyltransferase	0	1	1	0,000%	0,009%	0,024%
HTSR_0750	radical SAM protein	0	0	0	0,000%	0,000%	0,000%
HTSR_0751	formate dehydrogenase, gamma subunit	0	2	0	0,000%	0,019%	0,000%
HTSR_0752	hypothetical protein	0	0	0	0,000%	0,000%	0,000%

HTSR_0753	aldehyde ferredoxin oxidoreductase	0	15	0	0,000%	0,142%	0,000%
HTSR_0754	4Fe-4S ferredoxin iron-sulfur binding domain protein	0	0	0	0,000%	0,000%	0,000%
HTSR_0755	bacterio-opsin activator HTH domain-containing protein	0	0	1	0,000%	0,000%	0,024%
HTSR_0756	nucleoside diphosphate kinase	12	7	5	0,109%	0,066%	0,120%
HTSR_0757	50S ribosomal protein L24e	1	1	1	0,009%	0,009%	0,024%
HTSR_0758	30S ribosomal protein S28e	2	1	2	0,018%	0,009%	0,048%
HTSR_0759	50S ribosomal protein L7Ae	4	5	3	0,036%	0,047%	0,072%
HTSR_0760	bifunctional p-loop ATPase/acetyltransferase	0	13	14	0,000%	0,123%	0,335%
HTSR_0761	glutamyl-tRNA synthetase	16	20	14	0,145%	0,189%	0,335%
HTSR_0762	geranylgeranyl diphosphate synthase	5	9	5	0,045%	0,085%	0,120%
HTSR_0763	ribonuclease J	33	58	52	0,299%	0,548%	1,245%
HTSR_0764	isopentenyl phosphate kinase	0	5	1	0,000%	0,047%	0,024%
HTSR_0765	mevalonate kinase	8	16	4	0,073%	0,151%	0,096%
HTSR_0766	30S ribosomal protein S2	13	13	14	0,118%	0,123%	0,335%
HTSR_0767	enolase	20	19	15	0,181%	0,179%	0,359%
HTSR_0768	DNA-directed RNA polymerase subunit K	0	0	0	0,000%	0,000%	0,000%
HTSR_0769	DNA-directed RNA polymerase subunit N	2	4	2	0,018%	0,038%	0,048%
HTSR_0770	30S ribosomal protein S9	4	7	14	0,036%	0,066%	0,335%
HTSR_0771	50S ribosomal protein L13	7	7	12	0,064%	0,066%	0,287%
HTSR_0772	50S ribosomal protein L18e	12	8	9	0,109%	0,076%	0,215%
HTSR_0774	DNA-directed RNA polymerase subunit D	16	16	11	0,145%	0,151%	0,263%
HTSR_0775	30S ribosomal protein S11	9	8	9	0,082%	0,076%	0,215%
HTSR_0776	30S ribosomal protein S4	11	9	11	0,100%	0,085%	0,263%
HTSR_0777	30S ribosomal protein S13	16	8	17	0,145%	0,076%	0,407%
HTSR_0779	molybdenum cofactor biosynthesis protein A	6	0	2	0,054%	0,000%	0,048%
HTSR_0780	ATP-binding protein Mrp	15	16	8	0,136%	0,151%	0,191%
HTSR_0781	2-ketoglutarate ferredoxin oxidoreductase subunit alpha	35	59	43	0,318%	0,557%	1,029%
HTSR_0782	2-ketoglutarate ferredoxin oxidoreductase subunit beta	15	31	25	0,136%	0,293%	0,598%
HTSR_0783	AsnC family transcriptional regulator	4	3	1	0,036%	0,028%	0,024%
HTSR_0784	aspartate aminotransferase	13	14	9	0,118%	0,132%	0,215%
HTSR_0785	uridine phosphorylase	16	12	11	0,145%	0,113%	0,263%
HTSR_0786	cytidine deaminase	1	0	0	0,009%	0,000%	0,000%
HTSR_0787	hypothetical protein	0	0	0	0,000%	0,000%	0,000%
HTSR_0788	hypothetical protein	0	0	0	0,000%	0,000%	0,000%
HTSR_0789	bacterio-opsin activator HTH domain-containing protein	0	8	4	0,000%	0,076%	0,096%
HTSR_0790	bacterio-opsin activator HTH domain-containing protein	1	4	0	0,009%	0,038%	0,000%
HTSR_0791	hypothetical protein	0	0	0	0,000%	0,000%	0,000%
HTSR_0792	hypothetical protein	0	0	0	0,000%	0,000%	0,000%
HTSR_0793	ribose ABC transporter permease	1	1	0	0,009%	0,009%	0,000%
HTSR_0794	ribose ABC transporter permease	0	1	0	0,000%	0,009%	0,000%
HTSR_0795	ribose ABC transporter ATP-binding protein	1	2	1	0,009%	0,019%	0,024%
HTSR_0796	ABC transporter substrate-binding protein	16	13	15	0,145%	0,123%	0,359%
HTSR_0797	phosphomannomutase	12	21	12	0,109%	0,198%	0,287%
HTSR_0798	hypothetical protein	1	0	1	0,009%	0,000%	0,024%
HTSR_0799	type II secretion system protein E	2	11	23	0,018%	0,104%	0,551%
HTSR_0800	type II secretion system protein F	3	7	1	0,027%	0,066%	0,024%
HTSR_0801	type 11 methyltransferase	1	1	1	0,009%	0,009%	0,024%
HTSR_0803	hypothetical protein	6	8	9	0,054%	0,076%	0,215%
HTSR_0804	phosphohydrolase	3	8	4	0,027%	0,076%	0,096%
HTSR_0805	twin-arginine translocation protein, TatA/E family subunit	7	10	7	0,064%	0,094%	0,168%
HTSR_0807	cytochrome bd quinol oxidase subunit 1	6	8	4	0,054%	0,076%	0,096%
HTSR_0808	cytochrome bd quinol oxidase subunit 2	2	2	2	0,018%	0,019%	0,048%
HTSR_0809	HAD-superfamily hydrolase	2	0	7	0,018%	0,000%	0,168%
HTSR_0810	CBS domain-containing protein	9	5	4	0,082%	0,047%	0,096%
HTSR_0811	thioredoxin reductase	12	10	4	0,109%	0,094%	0,096%
HTSR_0812	cyclase/dehydrase	4	4	4	0,036%	0,038%	0,096%
HTSR_0813	TrmB family transcriptional regulator	15	17	8	0,136%	0,161%	0,191%
HTSR_0814	UDP-glucose 4-epimerase	2	7	2	0,018%	0,066%	0,048%
HTSR_0815	arsenic ABC transporter ATPase	11	23	16	0,100%	0,217%	0,383%
HTSR_0816	DNA gyrase subunit A	29	36	34	0,263%	0,340%	0,814%
HTSR_0817	DNA gyrase subunit B	11	20	48	0,100%	0,189%	1,149%
HTSR_0818	DNA topoisomerase VI subunit B	18	28	34	0,163%	0,265%	0,814%
HTSR_0819	DNA topoisomerase VI subunit A	1	2	9	0,009%	0,019%	0,215%
HTSR_0820	hypothetical protein	1	8	6	0,009%	0,076%	0,144%
HTSR_0821	proteasome subunit beta	6	17	19	0,054%	0,161%	0,455%
HTSR_0822	signal-transducing histidine kinase/response regulator	10	18	6	0,091%	0,170%	0,144%
HTSR_0823	glyoxalase/bleomycin resistance protein/dioxygenase	2	3	2	0,018%	0,028%	0,048%
HTSR_0824	hypothetical protein	0	0	0	0,000%	0,000%	0,000%
HTSR_0825	hypothetical protein	0	0	0	0,000%	0,000%	0,000%
HTSR_0826	XRE family transcriptional regulator	6	3	1	0,054%	0,028%	0,024%
HTSR_0827	phosphoribosylaminoimidazole synthetase	5	9	8	0,045%	0,085%	0,191%
HTSR_0828	hypothetical protein	0	0	0	0,000%	0,000%	0,000%
HTSR_0829	methyltransferase type 11	1	0	0	0,009%	0,000%	0,000%
HTSR_0830	6-pyruvoyltetrahydropterin synthase	0	0	0	0,000%	0,000%	0,000%
HTSR_0831	alcohol dehydrogenase zinc-binding domain protein	0	2	0	0,000%	0,019%	0,000%
HTSR_0832	glucose-6-phosphatase	0	0	0	0,000%	0,000%	0,000%
HTSR_0833	CDP-alcohol phosphatidyltransferase	0	0	0	0,000%	0,000%	0,000%
HTSR_0834	hypothetical protein	0	1	0	0,000%	0,009%	0,000%
HTSR_0835	glycosyl transferase group 1	0	2	6	0,000%	0,019%	0,144%
HTSR_0836	metallopeptidase	0	0	0	0,000%	0,000%	0,000%
HTSR_0837	TraB family protein	1	0	3	0,009%	0,000%	0,072%
HTSR_0838	hypothetical protein	0	0	0	0,000%	0,000%	0,000%
HTSR_0839	thioesterase superfamily protein	1	1	2	0,009%	0,009%	0,048%
HTSR_0840	hypothetical protein	1	1	0	0,009%	0,009%	0,000%
HTSR_0841	anaerobic glycerol-3-phosphate dehydrogenase subunit A	0	14	7	0,000%	0,132%	0,168%
HTSR_0842	anaerobic glycerol-3-phosphate dehydrogenase subunit C	0	2	1	0,000%	0,019%	0,024%
HTSR_0843	putative ATPase (AAA+ superfamily)	2	12	21	0,018%	0,113%	0,503%
HTSR_0844	hypothetical protein	0	0	0	0,000%	0,000%	0,000%
HTSR_0845	oligosaccharyltransferase AglB	11	9	4	0,100%	0,085%	0,096%
HTSR_0846	glycosyl transferase family 2	4	8	7	0,036%	0,076%	0,168%
HTSR_0847	GDP-mannose mannosyl hydrolase	0	0	4	0,000%	0,000%	0,096%
HTSR_0848	UDP-glucose 4-epimerase	13	16	25	0,118%	0,151%	0,598%
HTSR_0849	dTDP-glucose 4,6-dehydratase	18	7	29	0,163%	0,066%	0,694%
HTSR_0850	NAD-dependent dehydratase	0	0	0	0,000%	0,000%	0,000%
HTSR_0851	capsular polysaccharide biosynthesis protein	0	1	3	0,000%	0,009%	0,072%
HTSR_0852	group 1 glycosyl transferase	2	17	17	0,018%	0,161%	0,407%
HTSR_0853	N-acetylgalactosamine 6-sulfatase	5	17	27	0,045%	0,161%	0,646%
HTSR_0854	glucose-1-phosphate thymidylyltransferase	2	4	4	0,018%	0,038%	0,096%
HTSR_0855	hypothetical protein	2	4	7	0,018%	0,038%	0,168%
HTSR_0856	hypothetical protein / Tetratricopeptide TPR_2	1	1	5	0,009%	0,009%	0,120%
HTSR_0857	dTDP-glucose 4,6-dehydratase	11	18	22	0,100%	0,170%	0,527%
HTSR_0858	3-deoxy-manno-octulosonate cytidylyltransferase	7	0	4	0,064%	0,000%	0,096%
HTSR_0859	2-dehydro-3-deoxyphosphooctonate aldolase	6	2	15	0,054%	0,019%	0,359%
HTSR_0860	arabinose-5-phosphate isomerase	4	3	4	0,036%	0,028%	0,096%
HTSR_0863	CopG family transcriptional regulator	0	0	2	0,000%	0,000%	0,048%

HTSR_0864	capsule polysaccharide biosynthesis protein	4	21	16	0,036%	0,198%	0,383%
HTSR_0865	glycosyl transferase group 1	0	0	0	0,000%	0,000%	0,000%
HTSR_0866	glucose-1-phosphate thymidylyltransferase	5	4	5	0,045%	0,038%	0,120%
HTSR_0867	hypothetical protein	0	3	1	0,000%	0,028%	0,024%
HTSR_0868	hypothetical protein	0	0	0	0,000%	0,000%	0,000%
HTSR_0869	hypothetical protein	0	0	0	0,000%	0,000%	0,000%
HTSR_0870	glucose-1-phosphate thymidylyltransferase	0	0	0	0,000%	0,000%	0,000%
HTSR_0871	twitching motility protein PilT	1	3	5	0,009%	0,028%	0,120%
HTSR_0872	hypothetical protein	0	0	0	0,000%	0,000%	0,000%
HTSR_0873	hypothetical protein	0	0	0	0,000%	0,000%	0,000%
HTSR_0874	hypothetical protein	0	0	0	0,000%	0,000%	0,000%
HTSR_0875	hypothetical protein	3	0	0	0,027%	0,000%	0,000%
HTSR_0876	hypothetical protein	1	1	1	0,009%	0,009%	0,024%
HTSR_0877	hypothetical protein	1	1	0	0,009%	0,009%	0,000%
HTSR_0878	UDP-glucose 6-dehydrogenase	13	14	9	0,118%	0,132%	0,215%
HTSR_0879	ABC-type multidrug transport system, ATPase and permease component	10	13	7	0,091%	0,123%	0,168%
HTSR_0880	hypothetical protein	0	1	1	0,000%	0,009%	0,024%
HTSR_0881	hypothetical protein	0	0	0	0,000%	0,000%	0,000%
HTSR_0882	glucosamine--fructose-6-phosphate aminotransferase	9	21	11	0,082%	0,198%	0,263%
HTSR_0883	glucose-1-phosphate thymidylyltransferase	6	17	5	0,054%	0,161%	0,120%
HTSR_0884	hypothetical protein	0	0	0	0,000%	0,000%	0,000%
HTSR_0885	sugar-specific transcriptional regulator	3	10	10	0,027%	0,094%	0,239%
HTSR_0886	UDP-glucose 4-epimerase	1	2	0	0,009%	0,019%	0,000%
HTSR_0887	UDP-glucose 4-epimerase	4	18	5	0,036%	0,170%	0,120%
HTSR_0888	UDP-sulfoquinovose synthase	19	34	12	0,172%	0,321%	0,287%
HTSR_0889	hypothetical protein	0	0	0	0,000%	0,000%	0,000%
HTSR_0890	UDP-N-acetylglucosamine diphosphorylase	7	9	8	0,064%	0,085%	0,191%
HTSR_0891	glycosyl transferase family 2	2	6	8	0,018%	0,057%	0,191%
HTSR_0892	hypothetical protein	5	10	7	0,045%	0,094%	0,168%
HTSR_0893	hypothetical protein	1	1	2	0,009%	0,009%	0,048%
HTSR_0894	metallo-beta-lactamase family protein	1	5	0	0,009%	0,047%	0,000%
HTSR_0895	2-hydroxyhepta-2,4-diene-1,7-dioate isomerase	12	5	12	0,109%	0,047%	0,287%
HTSR_0896	hypothetical protein	0	1	0	0,000%	0,009%	0,000%
HTSR_0897	signal peptidase I	0	0	0	0,000%	0,000%	0,000%
HTSR_0898	phage protein	0	0	0	0,000%	0,000%	0,000%
HTSR_0899	hypothetical protein	0	7	0	0,000%	0,066%	0,000%
HTSR_0900	hypothetical protein	0	0	0	0,000%	0,000%	0,000%
HTSR_0901	hypothetical protein	0	10	0	0,000%	0,094%	0,000%
HTSR_0902	hypothetical protein	0	4	0	0,000%	0,038%	0,000%
HTSR_0903	von Willebrand factor type A	0	0	0	0,000%	0,000%	0,000%
HTSR_0904	hypothetical protein / Chitinase (EC 3.2.1.14)	0	1	0	0,000%	0,009%	0,000%
HTSR_0905	von Willebrand factor type A	1	3	0	0,009%	0,028%	0,000%
HTSR_0906	hypothetical protein	0	1	0	0,000%	0,009%	0,000%
HTSR_0907	hypothetical protein	0	3	0	0,000%	0,028%	0,000%
HTSR_0908	von Willebrand factor type A	0	2	0	0,000%	0,019%	0,000%
HTSR_0909	hypothetical protein	0	0	0	0,000%	0,000%	0,000%
HTSR_0910	hypothetical protein	3	7	0	0,027%	0,066%	0,000%
HTSR_0911	histidinol-phosphate aminotransferase	7	17	14	0,064%	0,161%	0,335%
HTSR_0912	adenylate kinase	2	0	2	0,018%	0,000%	0,048%
HTSR_0913	CDP-diacylglycerol--glycerol-3-phosphate 3-phosphatidyltransferase	1	1	2	0,009%	0,009%	0,048%
HTSR_0914	XRE family transcriptional regulator	3	2	4	0,027%	0,019%	0,096%
HTSR_0915	triosephosphate isomerase	6	7	6	0,054%	0,066%	0,144%
HTSR_0916	DNA polymerase IV	0	0	1	0,000%	0,000%	0,024%
HTSR_0917	GTP cyclohydrolase IIa	10	13	9	0,091%	0,123%	0,215%
HTSR_0918	sulfonate ABC transporter periplasmic substrate-binding protein	12	2	15	0,109%	0,019%	0,359%
HTSR_0919	hypothetical protein	1	0	0	0,009%	0,000%	0,000%
HTSR_0920	sulfonate ABC transporter permease	0	0	0	0,000%	0,000%	0,000%
HTSR_0921	sulfonate ABC transporter ATP-binding protein	0	0	0	0,000%	0,000%	0,000%
HTSR_0922	hypothetical protein	12	5	4	0,109%	0,047%	0,096%
HTSR_0923	molybdenum cofactor biosynthesis protein MoeA	18	16	11	0,163%	0,151%	0,263%
HTSR_0924	molybdenum cofactor biosynthesis protein MoeA	20	31	21	0,181%	0,293%	0,503%
HTSR_0925	Trk system potassium uptake protein TrkA	10	5	3	0,091%	0,047%	0,072%
HTSR_0926	hypothetical protein	5	4	5	0,045%	0,038%	0,120%
HTSR_0927	hypothetical protein	5	9	17	0,045%	0,085%	0,407%
HTSR_0928	cold-shock protein	0	0	0	0,000%	0,000%	0,000%
HTSR_0929	hypothetical protein	1	0	1	0,009%	0,000%	0,024%
HTSR_0930	phage integrase/site-specific recombinase	0	0	3	0,000%	0,000%	0,072%
HTSR_0931	hypothetical protein	3	0	1	0,027%	0,000%	0,024%
HTSR_0932	hypothetical protein	0	0	0	0,000%	0,000%	0,000%
HTSR_0933	site-specific recombinase, DNA invertase Pin	0	0	0	0,000%	0,000%	0,000%
HTSR_0934	type II restriction enzyme, methylase subunits	7	23	22	0,064%	0,217%	0,527%
HTSR_0935	hypothetical protein	0	0	0	0,000%	0,000%	0,000%
HTSR_0936	hypothetical protein	2	0	1	0,018%	0,000%	0,024%
HTSR_0937	hypothetical protein	0	0	0	0,000%	0,000%	0,000%
HTSR_0938	hypothetical protein	0	0	0	0,000%	0,000%	0,000%
HTSR_0939	hypothetical protein	0	0	0	0,000%	0,000%	0,000%
HTSR_0940	hypothetical protein	0	0	0	0,000%	0,000%	0,000%
HTSR_0941	hypothetical protein	0	0	0	0,000%	0,000%	0,000%
HTSR_0942	hypothetical protein	0	0	0	0,000%	0,000%	0,000%
HTSR_0943	hypothetical protein	0	0	0	0,000%	0,000%	0,000%
HTSR_0944	hypothetical protein	0	0	2	0,000%	0,000%	0,048%
HTSR_0945	IMP dehydrogenase	46	44	29	0,417%	0,416%	0,694%
HTSR_0946	hypothetical protein	0	0	0	0,000%	0,000%	0,000%
HTSR_0947	hypothetical protein	0	0	0	0,000%	0,000%	0,000%
HTSR_0948	hypothetical protein	0	0	0	0,000%	0,000%	0,000%
HTSR_0949	zinc/iron permease	0	0	0	0,000%	0,000%	0,000%
HTSR_0950	circadian clock protein, KaiC	10	2	17	0,091%	0,019%	0,407%
HTSR_0951	PAS domain s-box	8	27	12	0,073%	0,255%	0,287%
HTSR_0953	DHH family phosphoesterase	9	13	9	0,082%	0,123%	0,215%
HTSR_0954	photosystem reaction center subunit H	4	6	4	0,036%	0,057%	0,096%
HTSR_0955	DRTGG domain protein	16	30	16	0,145%	0,283%	0,383%
HTSR_0956	acetyl-CoA synthetase	26	39	19	0,236%	0,368%	0,455%
HTSR_0957	citrate transporter	0	6	1	0,000%	0,057%	0,024%
HTSR_0958	hypothetical protein	0	0	0	0,000%	0,000%	0,000%
HTSR_0959	SSSF family transport protein	1	3	2	0,009%	0,028%	0,048%
HTSR_0960	hypothetical protein	0	0	0	0,000%	0,000%	0,000%
HTSR_0961	integrase family protein	0	0	7	0,000%	0,000%	0,168%
HTSR_0962	hypothetical protein	0	0	1	0,000%	0,000%	0,024%
HTSR_0963	histidine kinase	0	0	6	0,000%	0,000%	0,144%
HTSR_0964	phosphoadenosine phosphosulfate reductase	20	21	20	0,181%	0,198%	0,479%
HTSR_0965	oxidoreductase (aldo-keto reductase family protein)	19	22	14	0,172%	0,208%	0,335%
HTSR_0966	electron transfer flavoprotein	15	22	22	0,136%	0,208%	0,527%
HTSR_0967	N-acetyltransferase GCN5	0	4	1	0,000%	0,038%	0,024%
HTSR_0968	molecular chaperone DnaJ	6	2	7	0,054%	0,019%	0,168%
HTSR_0969	molecular chaperone DnaK	25	21	28	0,227%	0,198%	0,670%

HTSR_0970	molecular chaperone GrpE	9	4	7	0,082%	0,038%	0,168%
HTSR_0971	hypothetical protein	1	0	1	0,009%	0,000%	0,024%
HTSR_0972	hypothetical protein	0	3	3	0,000%	0,028%	0,072%
HTSR_0973	RNA methylase	4	5	1	0,036%	0,047%	0,024%
HTSR_0974	3-isopropylmalate dehydratase	15	18	6	0,136%	0,170%	0,144%
HTSR_0975	aspartate aminotransferase	11	27	18	0,100%	0,255%	0,431%
HTSR_0976	nitrate/nitrite transporter	0	1	3	0,000%	0,009%	0,072%
HTSR_0977	translation-associated GTPase	4	8	5	0,036%	0,076%	0,120%
HTSR_0978	hypothetical protein	4	2	5	0,036%	0,019%	0,120%
HTSR_0979	hypothetical protein	3	2	1	0,027%	0,019%	0,024%
HTSR_0980	hypothetical protein	2	3	2	0,018%	0,028%	0,048%
HTSR_0981	proteasome-activating nucleotidase	30	15	35	0,272%	0,142%	0,838%
HTSR_0982	metallophosphoesterase	2	0	3	0,018%	0,000%	0,072%
HTSR_0983	chromosome segregation protein SMC	17	7	16	0,154%	0,066%	0,383%
HTSR_0984	hypothetical protein	0	0	0	0,000%	0,000%	0,000%
HTSR_0985	hypothetical protein	0	0	0	0,000%	0,000%	0,000%
HTSR_0986	DNA polymerase I	28	54	35	0,254%	0,510%	0,838%
HTSR_0987	signal transduction histidine kinase	0	0	4	0,000%	0,000%	0,096%
HTSR_0988	lactoylglutathione lyase	1	1	0	0,009%	0,009%	0,000%
HTSR_0989	short-chain dehydrogenase	3	8	6	0,027%	0,076%	0,144%
HTSR_0990	FeS assembly ATPase SufC	20	9	18	0,181%	0,085%	0,431%
HTSR_0991	FeS assembly protein SufB	13	2	14	0,118%	0,019%	0,335%
HTSR_0992	FeS assembly protein SufD	14	14	25	0,127%	0,132%	0,598%
HTSR_0994	sulfatase	3	2	7	0,027%	0,019%	0,168%
HTSR_0995	GtrA family protein	0	0	0	0,000%	0,000%	0,000%
HTSR_0996	hypothetical protein	2	3	1	0,018%	0,028%	0,024%
HTSR_0997	MscS Mechanosensitive ion channel	0	1	0	0,000%	0,009%	0,000%
HTSR_0998	Na+/H+ antiporter NhaC	1	3	0	0,009%	0,028%	0,000%
HTSR_0999	hypothetical protein	0	0	0	0,000%	0,000%	0,000%
HTSR_1000	signal transduction protein with CBS domains	4	7	5	0,036%	0,066%	0,120%
HTSR_1001	cold-shock protein	3	1	1	0,027%	0,009%	0,024%
HTSR_1002	metal dependent phosphohydrolase	10	1	8	0,091%	0,009%	0,191%
HTSR_1003	lysine transporter LysE	0	0	0	0,000%	0,000%	0,000%
HTSR_1004	threonine synthase	0	1	3	0,000%	0,009%	0,072%
HTSR_1005	putative GTPase	0	3	2	0,000%	0,028%	0,048%
HTSR_1006	ornithine carbamoyltransferase	16	11	8	0,145%	0,104%	0,191%
HTSR_1007	glutamine ABC transporter permease	4	2	2	0,036%	0,019%	0,048%
HTSR_1008	glutamine ABC transporter substrate-binding protein	7	6	2	0,064%	0,057%	0,048%
HTSR_1009	glutamine ABC transporter ATP-binding protein	3	2	3	0,027%	0,019%	0,072%
HTSR_1010	polar amino acid ABC transporter inner membrane protein	3	5	0	0,027%	0,047%	0,000%
HTSR_1011	phytoene dehydrogenase	11	24	16	0,100%	0,227%	0,383%
HTSR_1012	putative phosphoesterase, ICC	0	0	0	0,000%	0,000%	0,000%
HTSR_1013	Crp/Fnr family transcriptional regulator	8	6	6	0,073%	0,057%	0,144%
HTSR_1014	3-isopropylmalate dehydratase	9	8	5	0,082%	0,076%	0,120%
HTSR_1015	Zn-ribbon RNA-binding protein	0	0	0	0,000%	0,000%	0,000%
HTSR_1016	translation initiation factor IF-2 subunit alpha	13	7	5	0,118%	0,066%	0,120%
HTSR_1017	30S ribosomal protein S27e	3	3	4	0,027%	0,028%	0,096%
HTSR_1018	50S ribosomal protein L44e	1	2	3	0,009%	0,019%	0,072%
HTSR_1019	hypothetical protein	2	2	0	0,018%	0,019%	0,000%
HTSR_1020	hypothetical protein	0	4	0	0,000%	0,038%	0,000%
HTSR_1021	family 2 glycosyl transferase	6	7	12	0,054%	0,066%	0,287%
HTSR_1022	GtrA family protein	0	0	0	0,000%	0,000%	0,000%
HTSR_1023	adenine phosphoribosyltransferase	7	7	9	0,064%	0,066%	0,215%
HTSR_1024	monovalent cation/H+ antiporter subunit E	11	20	11	0,100%	0,189%	0,263%
HTSR_1025	monovalent cation/H+ antiporter subunit F	0	0	0	0,000%	0,000%	0,000%
HTSR_1026	monovalent cation/H+ antiporter subunit G	0	1	0	0,000%	0,009%	0,000%
HTSR_1027	monovalent cation/H+ antiporter subunit B	1	3	2	0,009%	0,028%	0,048%
HTSR_1028	monovalent cation/H+ antiporter subunit B	0	0	0	0,000%	0,000%	0,000%
HTSR_1029	monovalent cation/H+ antiporter subunit C	1	1	1	0,009%	0,009%	0,024%
HTSR_1030	monovalent cation/H+ antiporter subunit D	1	0	2	0,009%	0,000%	0,048%
HTSR_1031	monovalent cation/H+ antiporter subunit D	1	1	0	0,009%	0,009%	0,000%
HTSR_1032	monovalent cation/H+ antiporter subunit D	3	5	0	0,027%	0,047%	0,000%
HTSR_1033	phosphopantothenoylcysteine decarboxylase / phosphopantothenate--cysteine ligase	6	13	12	0,054%	0,123%	0,287%
HTSR_1034	hypothetical protein	0	0	1	0,000%	0,000%	0,024%
HTSR_1035	geranylgeranyl diphosphate synthase, type I	5	6	8	0,045%	0,057%	0,191%
HTSR_1036	hypothetical protein	1	0	0	0,009%	0,000%	0,000%
HTSR_1037	NAD+ kinase	3	5	5	0,027%	0,047%	0,120%
HTSR_1038	endonuclease III	6	2	4	0,054%	0,019%	0,096%
HTSR_1039	UbiD family decarboxylase	13	22	13	0,118%	0,208%	0,311%
HTSR_1040	hypothetical protein	2	0	3	0,018%	0,000%	0,072%
HTSR_1041	hypothetical protein	0	0	0	0,000%	0,000%	0,000%
HTSR_1042	3-octaprenyl-4-hydroxybenzoate carboxy-lyase UbiX	5	3	4	0,045%	0,028%	0,096%
HTSR_1043	diphosphomevalonate decarboxylase	8	9	7	0,073%	0,085%	0,168%
HTSR_1044	NADH dehydrogenase	10	18	13	0,091%	0,170%	0,311%
HTSR_1045	radical SAM protein, pyruvate-formate lyase-activating enzyme-like	1	7	1	0,009%	0,066%	0,024%
HTSR_1046	class II aldolase	0	1	0	0,000%	0,009%	0,000%
HTSR_1047	S-methyl-5-thioribose-1-phosphate isomerase	10	19	9	0,091%	0,179%	0,215%
HTSR_1048	thiamine-monophosphate kinase	4	2	3	0,036%	0,019%	0,072%
HTSR_1050	hypothetical protein	0	2	1	0,000%	0,019%	0,024%
HTSR_1051	hypothetical protein	0	0	2	0,000%	0,000%	0,048%
HTSR_1052	hypothetical protein	0	2	0	0,000%	0,019%	0,000%
HTSR_1053	(4Fe-4S)-binding protein	2	22	12	0,018%	0,208%	0,287%
HTSR_1054	pyrroline-5-carboxylate reductase	2	2	2	0,018%	0,019%	0,048%
HTSR_1055	glutamate 5-kinase	22	15	11	0,200%	0,142%	0,263%
HTSR_1056	glutamate-5-semialdehyde dehydrogenase	34	19	19	0,309%	0,179%	0,455%
HTSR_1057	hypothetical protein	0	4	1	0,000%	0,038%	0,024%
HTSR_1058	threonine synthase	3	12	8	0,027%	0,113%	0,191%
HTSR_1059	hypothetical protein	1	2	6	0,009%	0,019%	0,144%
HTSR_1060	hypothetical protein	2	3	17	0,018%	0,028%	0,407%
HTSR_1061	hypothetical protein	1	0	0	0,009%	0,000%	0,000%
HTSR_1063	tRNA (guanine37-N1)-methyltransferase	2	2	3	0,018%	0,019%	0,072%
HTSR_1064	diphthine synthase	0	2	3	0,000%	0,019%	0,072%
HTSR_1065	GTP-binding protein	7	10	11	0,064%	0,094%	0,263%
HTSR_1066	50S ribosomal protein L11	5	3	6	0,045%	0,028%	0,144%
HTSR_1067	50S ribosomal protein L1	18	15	15	0,163%	0,142%	0,359%
HTSR_1068	50S ribosomal protein L10	17	14	11	0,154%	0,132%	0,263%
HTSR_1069	50S ribosomal protein L12	0	0	0	0,000%	0,000%	0,000%
HTSR_1070	hypothetical protein	3	4	2	0,027%	0,038%	0,048%
HTSR_1071	hypothetical protein	6	8	6	0,054%	0,076%	0,144%
HTSR_1072	RNA methyltransferase, TrmH family, group 1	1	4	5	0,009%	0,038%	0,120%
HTSR_1073	multidrug DMT transporter	0	0	2	0,000%	0,000%	0,048%
HTSR_1074	AsnC family transcriptional regulator	8	12	3	0,073%	0,113%	0,072%
HTSR_1075	hypothetical protein	0	0	0	0,000%	0,000%	0,000%
HTSR_1076	glutamyl-tRNA(Gln) amidotransferase subunit E	31	47	25	0,281%	0,444%	0,598%
HTSR_1077	membrane protein	0	8	1	0,000%	0,076%	0,024%

HTSR_1078	hypothetical protein	3	2	2	0,027%	0,019%	0,048%
HTSR_1079	fumarate hydratase, class II	22	22	12	0,200%	0,208%	0,287%
HTSR_1080	flap endonuclease-1	0	2	5	0,000%	0,019%	0,120%
HTSR_1081	N-acetyltransferase GCN5	0	2	2	0,000%	0,019%	0,048%
HTSR_1082	menaquinone biosynthesis methyltransferase UbiE	0	0	3	0,000%	0,000%	0,072%
HTSR_1083	hypothetical protein	1	1	2	0,009%	0,009%	0,048%
HTSR_1084	hypothetical protein	0	0	0	0,000%	0,000%	0,000%
HTSR_1085	signal recognition particle 19 kDa protein	2	0	1	0,018%	0,000%	0,024%
HTSR_1086	glycyl aminopeptidase	4	1	8	0,036%	0,009%	0,191%
HTSR_1087	thermosome alpha subunit	27	16	27	0,245%	0,151%	0,646%
HTSR_1088	hypothetical protein	0	0	0	0,000%	0,000%	0,000%
HTSR_1089	putative dioxygenase / Candidate gene for the hypothesized phosphomevalonate decarboxylase; COG1355, Predicted diox	3	8	3	0,027%	0,076%	0,072%
HTSR_1090	DSBA oxidoreductase	2	3	9	0,018%	0,028%	0,215%
HTSR_1091	cysteine desulfurase	5	14	15	0,045%	0,132%	0,359%
HTSR_1092	thioredoxin domain protein	2	0	0	0,018%	0,000%	0,000%
HTSR_1093	TrmB family transcriptional regulator	1	0	0	0,009%	0,000%	0,000%
HTSR_1094	pyridine nucleotide-disulfide oxidoreductase	8	17	14	0,073%	0,161%	0,335%
HTSR_1095	SAM-dependent methyltransferase	4	12	8	0,036%	0,113%	0,191%
HTSR_1096	hypothetical protein	3	1	0	0,027%	0,009%	0,000%
HTSR_1097	peroxiredoxin	22	16	14	0,200%	0,151%	0,335%
HTSR_1098	deoxyribodipyrimidine photo-lyase	13	18	30	0,118%	0,170%	0,718%
HTSR_1099	superoxide dismutase, Fe-Mn family	2	1	1	0,018%	0,009%	0,024%
HTSR_1100	hypothetical protein	0	0	1	0,000%	0,000%	0,024%
HTSR_1102	threonine dehydrogenase and related Zn-dependentdehydrogenases	8	24	1	0,073%	0,227%	0,024%
HTSR_1103	transposase, IS605 OrfB family	0	0	11	0,000%	0,000%	0,263%
HTSR_1104	putative NADP-dependent oxidoreductase YncB	5	14	3	0,045%	0,132%	0,072%
HTSR_1105	1,4-dihydroxy-2-naphthoate octaprenyltransferase	0	0	0	0,000%	0,000%	0,000%
HTSR_1106	methyltransferase type 11 / 2-heptaprenyl-1,4-naphthoquinone methyltransferase (EC 2.1.1.163)	0	0	1	0,000%	0,000%	0,024%
HTSR_1107	dihydroxy-acid dehydratase	1	5	5	0,009%	0,047%	0,120%
HTSR_1108	major facilitator superfamily MFS_1	0	0	0	0,000%	0,000%	0,000%
HTSR_1109	transposase, IS605 OrfB family	0	0	0	0,000%	0,000%	0,000%
HTSR_1110	sugar phosphate permease	0	0	0	0,000%	0,000%	0,000%
HTSR_1111	catalase/peroxidase HPI	9	32	8	0,082%	0,302%	0,191%
HTSR_1112	hypothetical protein	0	0	0	0,000%	0,000%	0,000%
HTSR_1113	hypothetical protein	0	0	0	0,000%	0,000%	0,000%
HTSR_1114	hypothetical protein / Molybdenum cofactor biosynthesis protein MoaD	0	0	0	0,000%	0,000%	0,000%
HTSR_1115	hypothetical protein	0	0	0	0,000%	0,000%	0,000%
HTSR_1116	multi-sensor signal transduction histidine kinase	0	4	2	0,000%	0,038%	0,048%
HTSR_1117	hypothetical protein	0	0	0	0,000%	0,000%	0,000%
HTSR_1118	universal stress protein UspA	6	10	10	0,054%	0,094%	0,239%
HTSR_1119	hypothetical protein	0	0	0	0,000%	0,000%	0,000%
HTSR_1120	mechanosensitive ion channel	4	14	1	0,036%	0,132%	0,024%
HTSR_1121	FAD synthase	1	2	1	0,009%	0,019%	0,024%
HTSR_1122	aminoglycoside phosphotransferase	0	2	3	0,000%	0,019%	0,072%
HTSR_1123	apolipoprotein N-acyltransferase	1	6	14	0,009%	0,057%	0,335%
HTSR_1124	polyketide cyclase/dehydrase	6	11	12	0,054%	0,104%	0,287%
HTSR_1125	hypothetical protein	0	1	0	0,000%	0,009%	0,000%
HTSR_1126	phosphate uptake regulator PhoU	5	7	14	0,045%	0,066%	0,335%
HTSR_1127	N-acetylglucosamine-1-phosphate uridyltransferase	15	16	9	0,136%	0,151%	0,215%
HTSR_1128	competence/damage-inducible protein CinA	1	1	1	0,009%	0,009%	0,024%
HTSR_1129	hypothetical protein	3	2	6	0,027%	0,019%	0,144%
HTSR_1130	hypothetical protein	4	7	3	0,036%	0,066%	0,072%
HTSR_1131	transcription factor TFIIE subunit alpha	8	10	4	0,073%	0,094%	0,096%
HTSR_1132	tRNA (cytidine(56)-2'-O)-methyltransferase	2	1	1	0,018%	0,009%	0,024%
HTSR_1133	UDP-glucose 4-epimerase	6	14	17	0,054%	0,132%	0,407%
HTSR_1134	PadR family transcriptional regulator	4	3	2	0,036%	0,028%	0,048%
HTSR_1135	S-layer domain-containing protein	15	9	11	0,136%	0,085%	0,263%
HTSR_1136	RND transporter	0	2	0	0,000%	0,019%	0,000%
HTSR_1137	cation diffusion facilitator family transporter	0	1	1	0,000%	0,009%	0,024%
HTSR_1138	hypothetical protein	0	0	3	0,000%	0,000%	0,072%
HTSR_1139	transcription initiation factor IIB	9	7	27	0,082%	0,066%	0,646%
HTSR_1140	hypothetical protein	2	2	0	0,018%	0,019%	0,000%
HTSR_1141	RNA 3'-terminal phosphate cyclase (ATP)	0	9	11	0,000%	0,085%	0,263%
HTSR_1142	hypothetical protein	1	0	1	0,009%	0,000%	0,024%
HTSR_1143	2-dehydropantoate 2-reductase	0	2	7	0,000%	0,019%	0,168%
HTSR_1144	phosphoglycolate phosphatase	2	5	2	0,018%	0,047%	0,048%
HTSR_1145	hypothetical protein	0	1	0	0,000%	0,009%	0,000%
HTSR_1146	TrmB family transcriptional regulator	5	1	4	0,045%	0,009%	0,096%
HTSR_1147	molybdopterin-guanine dinucleotide biosynthesis protein B	4	8	7	0,036%	0,076%	0,168%
HTSR_1148	hypothetical protein	11	10	7	0,100%	0,094%	0,168%
HTSR_1149	hypothetical protein	2	0	0	0,018%	0,000%	0,000%
HTSR_1150	hypothetical protein	0	0	0	0,000%	0,000%	0,000%
HTSR_1151	mechanosensitive ion channel MscS	3	9	2	0,027%	0,085%	0,048%
HTSR_1152	electron transfer flavoprotein	16	4	14	0,145%	0,038%	0,335%
HTSR_1153	endonuclease III	0	0	2	0,000%	0,000%	0,048%
HTSR_1154	hypothetical protein	3	5	5	0,027%	0,047%	0,120%
HTSR_1155	TrkA-N domain protein	8	20	3	0,073%	0,189%	0,072%
HTSR_1156	AsnC family transcriptional regulator	5	9	3	0,045%	0,085%	0,072%
HTSR_1157	ATPase P	1	0	3	0,009%	0,000%	0,072%
HTSR_1158	heavy metal transport/detoxification protein	0	0	0	0,000%	0,000%	0,000%
HTSR_1159	hypothetical protein	0	0	0	0,000%	0,000%	0,000%
HTSR_1160	uracil-DNA glycosylase	1	0	1	0,009%	0,000%	0,024%
HTSR_1161	ribulose-bisphosphate carboxylase large chain	7	17	10	0,064%	0,161%	0,239%
HTSR_1162	fructose-1,6-bisphosphatase I	6	6	7	0,054%	0,057%	0,168%
HTSR_1163	fructose-bisphosphate aldolase, class I	12	17	9	0,109%	0,161%	0,215%
HTSR_1164	hypothetical protein	3	0	3	0,027%	0,000%	0,072%
HTSR_1165	3-ketoacyl-CoA thiolase	28	20	14	0,254%	0,189%	0,335%
HTSR_1166	hypothetical protein	5	3	1	0,045%	0,028%	0,024%
HTSR_1167	CBS/parB domain-containing protein	13	7	3	0,118%	0,066%	0,072%
HTSR_1168	D-glycerate dehydrogenase	22	18	18	0,200%	0,170%	0,431%
HTSR_1169	aspartate aminotransferase	27	36	26	0,245%	0,340%	0,622%
HTSR_1170	DHH family phosphoesterase / Kef-type K+ transport systems (NAD-binding component fused to domain related to exopoly	1	3	1	0,009%	0,028%	0,024%
HTSR_1171	NADH-quinone oxidoreductase subunit N	4	2	3	0,036%	0,019%	0,072%
HTSR_1172	NADH-quinone oxidoreductase subunit M	2	3	1	0,018%	0,028%	0,024%
HTSR_1173	NADH-quinone oxidoreductase subunit L	1	3	1	0,009%	0,028%	0,024%
HTSR_1174	NADH-quinone oxidoreductase subunit K	1	4	2	0,009%	0,038%	0,048%
HTSR_1175	NADH-quinone oxidoreductase subunit J2	1	1	1	0,009%	0,009%	0,024%
HTSR_1176	NADH-quinone oxidoreductase subunit J	0	0	0	0,000%	0,000%	0,000%
HTSR_1177	NADH-quinone oxidoreductase subunit I	2	4	3	0,018%	0,038%	0,072%
HTSR_1178	NADH-quinone oxidoreductase subunit H	6	6	4	0,054%	0,057%	0,096%
HTSR_1179	NADH-quinone oxidoreductase subunit C	36	48	47	0,327%	0,453%	1,125%
HTSR_1180	NADH-quinone oxidoreductase subunit B	13	17	21	0,118%	0,161%	0,503%
HTSR_1181	NADH-quinone oxidoreductase subunit A	7	5	3	0,064%	0,047%	0,072%
HTSR_1182	Fe-S oxidoreductase	11	0	25	0,100%	0,000%	0,598%
HTSR_1183	phosphoribosylaminoimidazole carboxylase, catalytic subunit	6	10	6	0,054%	0,094%	0,144%

HTSR_1184	phosphoribosylaminoimidazole carboxylase, ATPasesubunit	14	19	17	0,127%	0,179%	0,407%
HTSR_1185	membrane-bound mannosyltransferase	1	7	2	0,009%	0,066%	0,048%
HTSR_1186	hypothetical protein	0	0	0	0,000%	0,000%	0,000%
HTSR_1187	6,7-dimethyl-8-ribityllumazine synthase	7	7	4	0,064%	0,066%	0,096%
HTSR_1188	aspartate aminotransferase	11	14	7	0,100%	0,132%	0,168%
HTSR_1189	glutamate dehydrogenase (NADP+)	10	22	30	0,091%	0,208%	0,718%
HTSR_1190	hypothetical protein	0	3	5	0,000%	0,028%	0,120%
HTSR_1191	hypothetical protein	2	4	2	0,018%	0,038%	0,048%
HTSR_1192	hypothetical protein	3	3	3	0,027%	0,028%	0,072%
HTSR_1193	glycosyl transferase group 1	0	6	6	0,000%	0,057%	0,144%
HTSR_1194	glycosyl transferase group 1	3	8	13	0,027%	0,076%	0,311%
HTSR_1195	ribonuclease P, Rpr2/Rpp21 subunit	0	0	3	0,000%	0,000%	0,072%
HTSR_1196	RNA-binding protein YhbY	1	1	2	0,009%	0,009%	0,048%
HTSR_1197	mechanosensitive ion channel protein	7	13	8	0,064%	0,123%	0,191%
HTSR_1198	hypothetical protein	3	3	2	0,027%	0,028%	0,048%
HTSR_1199	UbiA prenyltransferase	1	1	0	0,009%	0,009%	0,000%
HTSR_1200	hypothetical protein	6	2	0	0,054%	0,019%	0,000%
HTSR_1202	short-chain dehydrogenase/reductase SDR	3	7	2	0,027%	0,066%	0,048%
HTSR_1203	hypothetical protein	0	0	0	0,000%	0,000%	0,000%
HTSR_1204	putative endonuclease containing a URI domain protein	0	0	2	0,000%	0,000%	0,048%
HTSR_1205	4-hydroxybenzoate polyprenyltransferase	0	0	0	0,000%	0,000%	0,000%
HTSR_1206	PAS domain S-box	0	2	1	0,000%	0,019%	0,024%
HTSR_1208	hypothetical protein	0	0	9	0,000%	0,000%	0,215%
HTSR_1209	hypothetical protein	0	0	5	0,000%	0,000%	0,120%
HTSR_1210	hypothetical protein	0	0	0	0,000%	0,000%	0,000%
HTSR_1211	hypothetical protein	1	0	0	0,009%	0,000%	0,000%
HTSR_1212	hypothetical protein	0	0	1	0,000%	0,000%	0,024%
HTSR_1213	hypothetical protein	0	0	2	0,000%	0,000%	0,048%
HTSR_1214	type II secretion system protein F	4	0	1	0,036%	0,000%	0,024%
HTSR_1215	type II secretion system protein E	13	19	31	0,118%	0,179%	0,742%
HTSR_1216	hypothetical protein	0	4	7	0,000%	0,038%	0,168%
HTSR_1217	ArsR family transcriptional regulator	5	2	7	0,045%	0,019%	0,168%
HTSR_1218	dihydroxy-acid dehydratase	15	7	22	0,136%	0,066%	0,527%
HTSR_1219	deoxyribonuclease/TRAM domain-containing protein	5	5	4	0,045%	0,047%	0,096%
HTSR_1220	methylenetetrahydrofolate reductase (NADPH)	30	51	22	0,272%	0,482%	0,527%
HTSR_1221	hypothetical protein	8	9	11	0,073%	0,085%	0,263%
HTSR_1222	universal stress protein UspA	4	17	14	0,036%	0,161%	0,335%
HTSR_1223	hypothetical protein	3	7	2	0,027%	0,066%	0,048%
HTSR_1224	PAS domain-containing sensor histidine kinase	4	2	13	0,036%	0,019%	0,311%
HTSR_1225	histidine kinase	8	11	5	0,073%	0,104%	0,120%
HTSR_1226	type I phosphodiesterase/nucleotide pyrophosphatase	20	21	18	0,181%	0,198%	0,431%
HTSR_1227	glycosyl transferase family 2	0	0	5	0,000%	0,000%	0,120%
HTSR_1228	hypothetical protein	1	1	2	0,009%	0,009%	0,048%
HTSR_1229	polysaccharide deacetylase	0	0	8	0,000%	0,000%	0,191%
HTSR_1230	hypothetical protein	0	3	1	0,000%	0,028%	0,024%
HTSR_1231	dolichyl-phosphate-mannose-protein mannosyltransferase	0	1	0	0,000%	0,009%	0,000%
HTSR_1232	MATE family efflux transporter	0	0	1	0,000%	0,000%	0,024%
HTSR_1233	universal stress protein UspA	4	14	19	0,036%	0,132%	0,455%
HTSR_1234	hypothetical protein	6	15	3	0,054%	0,142%	0,072%
HTSR_1235	hypothetical protein / YbaK/prolyl-tRNA synthetase associated region	1	1	0	0,009%	0,009%	0,000%
HTSR_1236	hypothetical protein	0	0	0	0,000%	0,000%	0,000%
HTSR_1237	hypothetical protein	0	0	0	0,000%	0,000%	0,000%
HTSR_1238	phosphoesterase RecJ domain protein	36	30	26	0,327%	0,283%	0,622%
HTSR_1239	phospholipase D/transphosphatidylase	0	0	0	0,000%	0,000%	0,000%
HTSR_1240	hypothetical protein	0	0	0	0,000%	0,000%	0,000%
HTSR_1241	PBS lyase HEAT-like repeat domain-containing protein	30	28	20	0,272%	0,265%	0,479%
HTSR_1242	CDP-diacylglycerol-serine O-phosphatidyltransferase	0	0	0	0,000%	0,000%	0,000%
HTSR_1243	30S ribosomal protein S3Ae	15	16	25	0,136%	0,151%	0,598%
HTSR_1244	hypothetical protein	0	0	0	0,000%	0,000%	0,000%
HTSR_1245	hypothetical protein	0	1	5	0,000%	0,009%	0,120%
HTSR_1246	30S ribosomal protein S15	16	11	22	0,145%	0,104%	0,527%
HTSR_1248	hypothetical protein	5	11	1	0,045%	0,104%	0,024%
HTSR_1249	beta-lactamase domain protein	0	0	1	0,000%	0,000%	0,024%
HTSR_1250	P-type ATPase	0	4	0	0,000%	0,038%	0,000%
HTSR_1251	mgtC family protein	0	1	0	0,000%	0,009%	0,000%
HTSR_1252	glutamate-1-semialdehyde aminotransferase	15	20	11	0,136%	0,189%	0,263%
HTSR_1253	dihydrodipicolinate reductase	3	11	4	0,027%	0,104%	0,096%
HTSR_1254	CBS domain-containing protein	2	1	3	0,018%	0,009%	0,072%
HTSR_1255	hypothetical protein	0	3	0	0,000%	0,028%	0,000%
HTSR_1256	hypothetical protein	1	6	3	0,009%	0,057%	0,072%
HTSR_1257	hypothetical protein	0	0	0	0,000%	0,000%	0,000%
HTSR_1258	cysteine synthase	0	0	0	0,000%	0,000%	0,000%
HTSR_1259	endonuclease	0	0	4	0,000%	0,000%	0,096%
HTSR_1260	hypothetical protein	0	0	0	0,000%	0,000%	0,000%
HTSR_1261	phosphoenolpyruvate carboxylase	52	59	49	0,472%	0,557%	1,173%
HTSR_1262	hypothetical protein	2	5	6	0,018%	0,047%	0,144%
HTSR_1263	DtxR family transcriptional regulator	7	0	3	0,064%	0,000%	0,072%
HTSR_1264	hypothetical protein	1	0	1	0,009%	0,000%	0,024%
HTSR_1265	hypothetical protein	0	0	1	0,000%	0,000%	0,024%
HTSR_1266	MATE family drug/sodium antiporter	1	0	0	0,009%	0,000%	0,000%
HTSR_1267	hypothetical protein	0	0	0	0,000%	0,000%	0,000%
HTSR_1268	hypothetical protein	0	0	0	0,000%	0,000%	0,000%
HTSR_1269	IS1341-type transposase	0	0	0	0,000%	0,000%	0,000%
HTSR_1270	prolyl-tRNA synthetase	13	16	14	0,118%	0,151%	0,335%
HTSR_1271	SOUL heme-binding protein	9	25	10	0,082%	0,236%	0,239%
HTSR_1272	short-chain dehydrogenase/reductase SDR	10	15	19	0,091%	0,142%	0,455%
HTSR_1273	hypothetical protein	0	1	0	0,000%	0,009%	0,000%
HTSR_1274	hypothetical protein	1	1	1	0,009%	0,009%	0,024%
HTSR_1275	ferrous iron transporter FeoB	4	4	4	0,036%	0,038%	0,096%
HTSR_1276	hypothetical protein	10	7	6	0,091%	0,066%	0,144%
HTSR_1277	histone deacetylase family protein	3	4	5	0,027%	0,038%	0,120%
HTSR_1278	hydantoinase/oxoprolinase	9	14	22	0,082%	0,132%	0,527%
HTSR_1279	hypothetical protein	0	0	0	0,000%	0,000%	0,000%
HTSR_1280	cysteinyl-tRNA synthetase	25	31	17	0,227%	0,293%	0,407%
HTSR_1281	hypothetical protein	0	4	4	0,000%	0,038%	0,096%
HTSR_1282	hypothetical protein	2	5	2	0,018%	0,047%	0,048%
HTSR_1283	hypothetical protein	1	0	0	0,009%	0,000%	0,000%
HTSR_1284	adenylosuccinate synthetase	38	26	25	0,345%	0,246%	0,598%
HTSR_1285	hypothetical protein	7	5	7	0,064%	0,047%	0,168%
HTSR_1286	hypothetical protein	18	16	14	0,163%	0,151%	0,335%
HTSR_1287	metal-binding protein	3	0	1	0,027%	0,000%	0,024%
HTSR_1288	cell surface protein	0	0	0	0,000%	0,000%	0,000%
HTSR_1289	sulfite oxidase-like oxidoreductase	1	0	4	0,009%	0,000%	0,096%
HTSR_1290	isochorismate synthase	6	3	12	0,054%	0,028%	0,287%
HTSR_1291	2-succinyl-5-enolpyruvyl-6-hydroxy-3-cyclohexene-1-carboxylic-acid synthase	11	15	15	0,100%	0,142%	0,359%

HTSR_1292	naphthoate synthase	11	22	15	0,100%	0,208%	0,359%
HTSR_1293	1,4-dihydroxy-2-naphthoate octaprenyltransferase	1	3	3	0,009%	0,028%	0,072%
HTSR_1294	O-succinylbenzoate synthase	0	2	2	0,000%	0,019%	0,048%
HTSR_1295	O-succinylbenzoate-CoA ligase	3	17	11	0,027%	0,161%	0,263%
HTSR_1296	transcription repressor	6	5	3	0,054%	0,047%	0,072%
HTSR_1297	aspartyl-tRNA(Asn)/glutamyl-tRNA(Gln) amidotransferase subunit A	17	14	22	0,154%	0,132%	0,527%
HTSR_1298	aspartyl-tRNA(Asn)/glutamyl-tRNA(Gln) amidotransferase subunit C	2	2	1	0,018%	0,019%	0,024%
HTSR_1299	phosphoesterase	2	0	5	0,018%	0,000%	0,120%
HTSR_1300	phosphoribosylformylglycinamidine synthase II	18	33	23	0,163%	0,312%	0,551%
HTSR_1301	hypothetical protein	0	0	1	0,000%	0,000%	0,024%
HTSR_1302	hypothetical protein	0	0	0	0,000%	0,000%	0,000%
HTSR_1303	imidazole glycerol phosphate synthase subunit HisF	4	4	2	0,036%	0,038%	0,048%
HTSR_1304	DNA-directed RNA polymerase subunit L	3	1	2	0,027%	0,009%	0,048%
HTSR_1305	rhomboid family protein	0	0	0	0,000%	0,000%	0,000%
HTSR_1306	RNA methylase	0	0	0	0,000%	0,000%	0,000%
HTSR_1307	diphthamide biosynthesis protein	1	0	9	0,009%	0,000%	0,215%
HTSR_1308	beta-lactamase domain protein	3	0	1	0,027%	0,000%	0,024%
HTSR_1309	hypothetical protein	25	2	15	0,227%	0,019%	0,359%
HTSR_1310	hypothetical protein	2	1	2	0,018%	0,009%	0,048%
HTSR_1311	phosphate transport system regulatory protein PhoU	13	11	9	0,118%	0,104%	0,215%
HTSR_1312	ferredoxin	2	1	1	0,018%	0,009%	0,024%
HTSR_1313	geranylgeranyl reductase	18	17	12	0,163%	0,161%	0,287%
HTSR_1314	Na+/H+ antiporter NhaC	8	11	2	0,073%	0,104%	0,048%
HTSR_1315	hypothetical protein	2	8	2	0,018%	0,076%	0,048%
HTSR_1316	30S ribosomal protein S10b	1	2	8	0,009%	0,019%	0,191%
HTSR_1317	NUDIX hydrolase	7	4	11	0,064%	0,038%	0,263%
HTSR_1318	hypothetical protein	0	2	2	0,000%	0,019%	0,048%
HTSR_1319	major facilitator superfamily MFS_1	0	0	0	0,000%	0,000%	0,000%
HTSR_1320	transcription termination factor Tfs	0	0	1	0,000%	0,000%	0,024%
HTSR_1321	tRNA (adenine57-N1/adenine58-N1)-methyltransferase	1	3	1	0,009%	0,028%	0,024%
HTSR_1322	nascent polypeptide-associated complex protein	5	1	2	0,045%	0,009%	0,048%
HTSR_1323	PUA domain containing protein	1	2	3	0,009%	0,019%	0,072%
HTSR_1324	cationic amino acid transporter	0	0	0	0,000%	0,000%	0,000%
HTSR_1326	universal stress protein UspA	10	9	8	0,091%	0,085%	0,191%
HTSR_1327	NYN domain-containing protein	2	3	5	0,018%	0,028%	0,120%
HTSR_1328	metallophosphoesterase	1	3	1	0,009%	0,028%	0,024%
HTSR_1329	4-hydroxy-tetrahydrodipicolinate synthase	29	21	11	0,263%	0,198%	0,263%
HTSR_1330	4-hydroxy-tetrahydrodipicolinate reductase	8	11	6	0,073%	0,104%	0,144%
HTSR_1331	2,3,4,5-tetrahydropyridine-2-carboxylate N-succinyltransferase	15	12	11	0,136%	0,113%	0,263%
HTSR_1332	diaminopimelate decarboxylase	17	16	17	0,154%	0,151%	0,407%
HTSR_1333	diaminopimelate epimerase	11	4	4	0,100%	0,038%	0,096%
HTSR_1334	succinyl-diaminopimelate desuccinylase	7	16	10	0,064%	0,151%	0,239%
HTSR_1335	adenylosuccinate lyase	23	29	29	0,209%	0,274%	0,694%
HTSR_1336	phosphoribosylaminoimidazolecarboxamide formyltransferase / IMP cyclohydrolase	16	29	21	0,145%	0,274%	0,503%
HTSR_1337	ferrochelatase	9	20	5	0,082%	0,189%	0,120%
HTSR_1338	uroporphyrinogen decarboxylase	4	14	13	0,036%	0,132%	0,311%
HTSR_1339	glycosyl transferase family 1	6	14	22	0,054%	0,132%	0,527%
HTSR_1340	guanosine monophosphate reductase	5	16	11	0,045%	0,151%	0,263%
HTSR_1341	glycerol-1-phosphate dehydrogenase [NAD(P)+]	16	25	20	0,145%	0,236%	0,479%
HTSR_1342	NAD-dependent epimerase/dehydratase	7	13	7	0,064%	0,123%	0,168%
HTSR_1343	transcriptional regulator	0	0	0	0,000%	0,000%	0,000%
HTSR_1344	chaperone protein TorD	3	4	0	0,027%	0,038%	0,000%
HTSR_1345	polysulfide reductase NrfD	7	8	2	0,064%	0,076%	0,048%
HTSR_1346	4Fe-4S ferredoxin iron-sulfur binding domain protein	11	10	3	0,100%	0,094%	0,072%
HTSR_1347	molybdopterin oxidoreductase	49	63	27	0,445%	0,595%	0,646%
HTSR_1348	rhodanese domain protein	26	32	19	0,236%	0,302%	0,455%
HTSR_1349	bacterio-opsin activator HTH domain-containing protein	1	0	2	0,009%	0,000%	0,048%
HTSR_1350	bacterio-opsin activator HTH domain-containing protein	1	0	0	0,009%	0,000%	0,000%
HTSR_1351	anaerobic dehydrogenase subunit	2	0	1	0,018%	0,000%	0,024%
HTSR_1352	hypothetical protein	0	0	0	0,000%	0,000%	0,000%
HTSR_1353	flagella-related protein E	11	2	6	0,100%	0,019%	0,144%
HTSR_1354	flagella-related protein F	0	0	0	0,000%	0,000%	0,000%
HTSR_1355	flagella-related protein G	2	0	6	0,018%	0,000%	0,144%
HTSR_1356	flagella-related protein H	3	5	12	0,027%	0,047%	0,287%
HTSR_1357	flagella-related protein I	1	0	3	0,009%	0,000%	0,072%
HTSR_1358	flagella-related protein J	0	0	0	0,000%	0,000%	0,000%
HTSR_1359	septum site-determining protein MinD	0	0	0	0,000%	0,000%	0,000%
HTSR_1360	hypothetical protein	0	0	0	0,000%	0,000%	0,000%
HTSR_1361	hypothetical protein	0	6	3	0,000%	0,057%	0,072%
HTSR_1362	chemotaxis protein CheW	1	0	2	0,009%	0,000%	0,048%
HTSR_1363	methyl-accepting chemotaxis sensory transducer	12	14	18	0,109%	0,132%	0,431%
HTSR_1364	metal-dependent hydrolase, beta-lactamase superfamily II	4	6	5	0,036%	0,057%	0,120%
HTSR_1365	hypothetical protein	11	5	7	0,100%	0,047%	0,168%
HTSR_1366	hypothetical protein	1	0	0	0,009%	0,000%	0,000%
HTSR_1367	chemotaxis protein CheW	3	6	8	0,027%	0,057%	0,191%
HTSR_1368	chemotaxis protein CheY	3	0	2	0,027%	0,000%	0,048%
HTSR_1369	chemotaxis-specific methylesterase CheB	6	7	6	0,054%	0,066%	0,144%
HTSR_1370	chemotaxis protein CheA	28	24	25	0,254%	0,227%	0,598%
HTSR_1371	chemotaxis protein CheC1	3	0	5	0,027%	0,000%	0,120%
HTSR_1372	chemotaxis protein CheC2	3	4	4	0,027%	0,038%	0,096%
HTSR_1373	chemotaxis protein CheD	0	0	2	0,000%	0,000%	0,048%
HTSR_1374	chemotaxis protein CheR	7	4	9	0,064%	0,038%	0,215%
HTSR_1375	chemotaxis protein CheF2	2	1	17	0,018%	0,009%	0,407%
HTSR_1376	chemotaxis protein CheF1	0	3	12	0,000%	0,028%	0,287%
HTSR_1377	phycocyanin alpha phycocyanobilin lyase	3	2	3	0,027%	0,019%	0,072%
HTSR_1378	flagellin	0	0	2	0,000%	0,000%	0,048%
HTSR_1379	hypothetical protein	1	0	1	0,009%	0,000%	0,024%
HTSR_1380	flagellin	10	4	12	0,091%	0,038%	0,287%
HTSR_1381	flagellin	4	1	9	0,036%	0,009%	0,215%
HTSR_1382	hypothetical protein	0	0	0	0,000%	0,000%	0,000%
HTSR_1383	flagella E	6	1	7	0,054%	0,009%	0,168%
HTSR_1384	mRNA 3-end processing factor-like protein	22	28	33	0,200%	0,265%	0,790%
HTSR_1385	endonuclease III	3	3	0	0,027%	0,028%	0,000%
HTSR_1386	hypothetical protein	4	0	2	0,036%	0,000%	0,048%
HTSR_1387	hypothetical protein	0	0	0	0,000%	0,000%	0,000%
HTSR_1388	phosphoserine phosphatase	42	25	32	0,381%	0,236%	0,766%
HTSR_1389	hypothetical protein	0	6	2	0,000%	0,057%	0,048%
HTSR_1390	ATP binding protein	0	0	3	0,000%	0,000%	0,072%
HTSR_1391	glucose-1-phosphate thymidylyltransferase	1	5	5	0,009%	0,047%	0,120%
HTSR_1392	shikimate dehydrogenase	6	6	5	0,054%	0,057%	0,120%
HTSR_1393	sodium/calcium exchanger membrane region	0	1	3	0,000%	0,009%	0,072%
HTSR_1394	hypothetical protein	0	2	1	0,000%	0,019%	0,024%
HTSR_1395	D-aminoacyl-tRNA deacylase	14	9	10	0,127%	0,085%	0,239%
HTSR_1396	cell division protein FtsZ	13	3	17	0,118%	0,028%	0,407%
HTSR_1397	preprotein translocase subunit SecE	1	3	3	0,009%	0,028%	0,072%

HTSR_1398	transcription antitermination protein NusG	9	7	1	0,082%	0,066%	0,024%
HTSR_1399	hypothetical protein	1	1	2	0,009%	0,009%	0,048%
HTSR_1400	metal-dependent phosphoesterase (PHP family)-like protein	1	0	9	0,009%	0,000%	0,215%
HTSR_1401	competence/damage-inducible protein CinA	2	1	3	0,018%	0,009%	0,072%
HTSR_1402	arsenite-transporting ATPase	8	12	3	0,073%	0,113%	0,072%
HTSR_1403	hydroxypyruvate reductase	8	11	13	0,073%	0,104%	0,311%
HTSR_1404	short-chain dehydrogenase/reductase	8	16	14	0,073%	0,151%	0,335%
HTSR_1405	deoxyribonuclease V	0	0	0	0,000%	0,000%	0,000%
HTSR_1406	rhomboid family intramembrane serine protease	1	1	2	0,009%	0,009%	0,048%
HTSR_1407	hypothetical protein	4	2	4	0,036%	0,019%	0,096%
HTSR_1408	DNA polymerase IV (family X)	11	19	19	0,100%	0,179%	0,455%
HTSR_1409	hypothetical protein	0	0	0	0,000%	0,000%	0,000%
HTSR_1410	hypothetical protein	2	0	3	0,018%	0,000%	0,072%
HTSR_1411	hypothetical protein	3	5	5	0,027%	0,047%	0,120%
HTSR_1412	Abi/CAAX family protein	1	1	1	0,009%	0,009%	0,024%
HTSR_1413	hypothetical protein	0	0	18	0,000%	0,000%	0,431%
HTSR_1414	DNA topoisomerase I	31	24	25	0,281%	0,227%	0,598%
HTSR_1415	hypothetical protein	0	0	0	0,000%	0,000%	0,000%
HTSR_1416	aspartyl-tRNA(Asn)/glutamyl-tRNA(Gln) amidotransferase subunit B	13	20	15	0,118%	0,189%	0,359%
HTSR_1417	hypothetical protein	0	0	1	0,000%	0,000%	0,024%
HTSR_1418	chromosome segregation protein SMC	6	0	7	0,054%	0,000%	0,168%
HTSR_1419	chromosome segregation and condensation protein ScpA	0	0	1	0,000%	0,000%	0,024%
HTSR_1420	5'-methylthioadenosine phosphorylase	21	23	17	0,191%	0,217%	0,407%
HTSR_1421	xanthine phosphoribosyltransferase	7	6	7	0,064%	0,057%	0,168%
HTSR_1422	hypothetical protein	4	4	4	0,036%	0,038%	0,096%
HTSR_1423	phosphoenolpyruvate synthase	34	52	34	0,309%	0,491%	0,814%
HTSR_1424	peptidase M48	0	1		0,000%	0,009%	0,000%
HTSR_1425	tyrosine decarboxylase / aspartate 1-decarboxylase	4	0	7	0,036%	0,000%	0,168%
HTSR_1426	hypothetical protein	0	0	1	0,000%	0,000%	0,024%
HTSR_1427	hypothetical protein	0	3	1	0,000%	0,028%	0,024%
HTSR_1428	methionyl-tRNA synthetase	35	45	35	0,318%	0,425%	0,838%
HTSR_1429	pyruvate kinase	16	33	15	0,145%	0,312%	0,359%
HTSR_1430	protease	2	2	3	0,018%	0,019%	0,072%
HTSR_1431	phosphoesterase	22	48	22	0,200%	0,453%	0,527%
HTSR_1432	MarR family transcriptional regulator	1	1	0	0,009%	0,009%	0,000%
HTSR_1433	hypothetical protein	0	0	0	0,000%	0,000%	0,000%
HTSR_1434	dolichol-P-glucose synthetase	1	4	6	0,009%	0,038%	0,144%
HTSR_1435	transcription initiation factor IIB 2	13	0	20	0,118%	0,000%	0,479%
HTSR_1436	3-dehydroquinate dehydratase I	10	8	1	0,091%	0,076%	0,024%
HTSR_1437	hypothetical protein	2	1	4	0,018%	0,009%	0,096%
HTSR_1438	3-dehydroquinate synthase	13	24	21	0,118%	0,227%	0,503%
HTSR_1439	fructose-bisphosphate aldolase	10	20	4	0,091%	0,189%	0,096%
HTSR_1440	tryptophan synthase alpha chain	7	2	4	0,064%	0,019%	0,096%
HTSR_1441	tryptophan synthase beta chain	15	19	12	0,136%	0,179%	0,287%
HTSR_1442	indole-3-glycerol phosphate synthase	9	0	5	0,082%	0,000%	0,120%
HTSR_1443	methylated-DNA--protein-cysteine methyltransferase	6	8	5	0,054%	0,076%	0,120%
HTSR_1444	abortive infection protein	1	0	1	0,009%	0,000%	0,024%
HTSR_1445	ATP-dependent protease LonB	14	11	33	0,127%	0,104%	0,790%
HTSR_1446	nicotinamide-nucleotide adenylyltransferase NadM	1	6	5	0,009%	0,057%	0,120%
HTSR_1447	S-adenosylmethionine hydroxide adenosyltransferase	4	4	3	0,036%	0,038%	0,072%
HTSR_1448	amidohydrolase	3	7	2	0,027%	0,066%	0,048%
HTSR_1449	glycosyltransferase, type 2	0	0	10	0,000%	0,000%	0,239%
HTSR_1450	S-adenosylmethionine-dependent methyltransferase	5	8	14	0,045%	0,076%	0,335%
HTSR_1451	ArsR family transcriptional regulator	1	0	1	0,009%	0,000%	0,024%
HTSR_1452	hypothetical protein	2	1	0	0,018%	0,009%	0,000%
HTSR_1453	deoxyribonuclease	9	11	6	0,082%	0,104%	0,144%
HTSR_1454	radical SAM protein	3	1	10	0,027%	0,009%	0,239%
HTSR_1455	hypothetical protein	0	0	0	0,000%	0,000%	0,000%
HTSR_1456	molecular chaperone Hsp20	5	5	5	0,045%	0,047%	0,120%
HTSR_1457	coenzyme A pyrophosphatase / NUDIX hydrolase	0	0	2	0,000%	0,000%	0,048%
HTSR_1458	hypothetical protein	7	4	4	0,064%	0,038%	0,096%
HTSR_1459	major facilitator superfamily MFS_1	1	1	0	0,009%	0,009%	0,000%
HTSR_1460	hypothetical protein	0	2	2	0,000%	0,019%	0,048%
HTSR_1461	hypothetical protein	0	0	0	0,000%	0,000%	0,000%
HTSR_1462	hypothetical protein	0	0	0	0,000%	0,000%	0,000%
HTSR_1463	hypothetical protein	0	0	0	0,000%	0,000%	0,000%
HTSR_1464	hypothetical protein	0	0	0	0,000%	0,000%	0,000%
HTSR_1465	type II secretion system transmembrane protein	0	1	0	0,000%	0,009%	0,000%
HTSR_1466	type II secretion system protein E	0	1	0	0,000%	0,009%	0,000%
HTSR_1467	hypothetical protein	0	0	0	0,000%	0,000%	0,000%
HTSR_1468	hypothetical protein	0	0	0	0,000%	0,000%	0,000%
HTSR_1469	type I phosphodiesterase/nucleotide pyrophosphatase	22	21	20	0,200%	0,198%	0,479%
HTSR_1470	inorganic pyrophosphatase	12	13	8	0,109%	0,123%	0,191%
HTSR_1471	PadR family transcriptional regulator	5	2	5	0,045%	0,019%	0,120%
HTSR_1472	hypothetical protein	3	0	1	0,027%	0,000%	0,024%
HTSR_1473	ribonuclease HI	5	1	2	0,045%	0,009%	0,048%
HTSR_1474	transcription initiation factor TFB	9	13	33	0,082%	0,123%	0,790%
HTSR_1475	geranylgeranylglyceryl phosphate synthase	0	3	1	0,000%	0,028%	0,024%
HTSR_1476	aspartyl-tRNA synthetase	19	29	21	0,172%	0,274%	0,503%
HTSR_1477	sugar kinase	4	3	5	0,036%	0,028%	0,120%
HTSR_1478	hypothetical protein / Phosphopantothenate synthetase, archaeal	5	1	5	0,045%	0,009%	0,120%
HTSR_1479	phosphomethylpyrimidine kinase	6	3	6	0,054%	0,028%	0,144%
HTSR_1480	dCTP deaminase	9	9	12	0,082%	0,085%	0,287%
HTSR_1481	peptidyl-tRNA hydrolase, PTH2 family	4	5	5	0,036%	0,047%	0,120%
HTSR_1482	tRNA pseudouridine synthase D	3	12	12	0,027%	0,113%	0,287%
HTSR_1483	hypothetical protein	2	2	3	0,018%	0,019%	0,072%
HTSR_1484	metal-binding protein	1	2	6	0,009%	0,019%	0,144%
HTSR_1485	50S ribosomal protein L37Ae	3	3	6	0,027%	0,028%	0,144%
HTSR_1486	DNA-directed RNA polymerase subunit P	1	2	3	0,009%	0,019%	0,072%
HTSR_1487	hypothetical protein	0	0	1	0,000%	0,000%	0,024%
HTSR_1488	periplasmic serine proteinase	7	6	5	0,064%	0,057%	0,120%
HTSR_1489	prefoldin subunit beta	9	9	4	0,082%	0,085%	0,096%
HTSR_1490	hypothetical protein	1	1	1	0,009%	0,009%	0,024%
HTSR_1491	membrane protein	5	1	4	0,045%	0,009%	0,096%
HTSR_1492	GMP synthase (glutamine-hydrolysing)	5	3	3	0,045%	0,028%	0,072%
HTSR_1493	hypothetical protein	0	0	0	0,000%	0,000%	0,000%
HTSR_1494	hypothetical protein	1	0	7	0,009%	0,000%	0,168%
HTSR_1495	2-isopropylmalate synthase	15	9	8	0,136%	0,085%	0,191%
HTSR_1496	succinic semialdehyde dehydrogenase	5	18	13	0,045%	0,170%	0,311%
HTSR_1498	secretion system protein	0	0	5	0,000%	0,000%	0,120%
HTSR_1499	membrane protein	0	0	0	0,000%	0,000%	0,000%
HTSR_1500	hypothetical protein	0	0	0	0,000%	0,000%	0,000%
HTSR_1501	chromosome partitioning protein ParA	0	0	2	0,000%	0,000%	0,048%
HTSR_1502	ATP-grasp superfamily enzyme	2	9	0	0,018%	0,085%	0,000%
HTSR_1503	hypothetical protein	1	4	3	0,009%	0,038%	0,072%

HTSR_1504	peroxiredoxin, OsmC subfamily	1	1	0	0,009%	0,009%	0,000%
HTSR_1506	CopG family transcriptional regulator	0	0	0	0,000%	0,000%	0,000%
HTSR_1507	cell division protein FtsZ	17	7	24	0,154%	0,066%	0,574%
HTSR_1508	arginosuccinate synthase	0	3	4	0,000%	0,028%	0,096%
HTSR_1509	peptidase M29 aminopeptidase II	11	24	17	0,100%	0,227%	0,407%
HTSR_1510	SAM-dependent methyltransferase	0	1	6	0,000%	0,009%	0,144%
HTSR_1511	endonuclease IV	0	0	3	0,000%	0,000%	0,072%
HTSR_1512	serine/threonine protein kinase	7	10	19	0,064%	0,094%	0,455%
HTSR_1513	ATPase	4	3	14	0,036%	0,028%	0,335%
HTSR_1514	hypothetical protein	0	0	0	0,000%	0,000%	0,000%
HTSR_1515	DNA recombination protein RmuC	3	3	6	0,027%	0,028%	0,144%
HTSR_1516	ATP-dependent DNA ligase	0	4	9	0,000%	0,038%	0,215%
HTSR_1517	hypothetical protein	1	0	8	0,009%	0,000%	0,191%
HTSR_1518	50S ribosomal protein L15e	4	3	8	0,036%	0,028%	0,191%
HTSR_1519	respiratory-chain NADH dehydrogenase domain 51 kDa subunit	2	5	0	0,018%	0,047%	0,000%
HTSR_1520	two-component sensor histidine kinase	0	0	2	0,000%	0,000%	0,048%
HTSR_1521	chaperone protein TorD	0	0	0	0,000%	0,000%	0,000%
HTSR_1522	molybdopterin oxidoreductase	0	56	0	0,000%	0,529%	0,000%
HTSR_1523	polysulfide reductase NrfD	0	8	0	0,000%	0,076%	0,000%
HTSR_1524	4Fe-4S ferredoxin iron-sulfur binding domain protein	0	6	0	0,000%	0,057%	0,000%
HTSR_1525	bacterio-opsin activator HTH domain-containing protein	1	2	4	0,009%	0,019%	0,096%
HTSR_1526	proteasome beta subunit	7	8	16	0,064%	0,076%	0,383%
HTSR_1527	proteasome alpha subunit	24	31	27	0,218%	0,293%	0,646%
HTSR_1528	translation elongation factor aEF-2	56	96	67	0,508%	0,907%	1,604%
HTSR_1529	30S ribosomal protein S7	22	16	21	0,200%	0,151%	0,503%
HTSR_1530	30S ribosomal protein S12	7	8	13	0,064%	0,076%	0,311%
HTSR_1531	transcription elongation factor NusA	0	0	0	0,000%	0,000%	0,000%
HTSR_1532	DNA-directed RNA polymerase subunit A''	26	32	28	0,236%	0,302%	0,670%
HTSR_1533	DNA-directed RNA polymerase subunit A'	45	65	53	0,408%	0,614%	1,269%
HTSR_1534	DNA-directed RNA polymerase subunit B'	19	37	35	0,172%	0,350%	0,838%
HTSR_1535	DNA-directed RNA polymerase subunit B''	31	54	56	0,281%	0,510%	1,340%
HTSR_1536	DNA-directed RNA polymerase subunit H	2	1	1	0,018%	0,009%	0,024%
HTSR_1538	TetR family transcriptional regulator	6	12	18	0,054%	0,113%	0,431%
HTSR_1539	exo-alpha-sialidase	17	29	20	0,154%	0,274%	0,479%
HTSR_1540	RND transporter	3	8	1	0,027%	0,076%	0,024%
HTSR_1541	hypothetical protein	0	0	0	0,000%	0,000%	0,000%
HTSR_1542	hypothetical protein	14	21	10	0,127%	0,198%	0,239%
HTSR_1543	transcriptional regulator	5	3	4	0,045%	0,028%	0,096%
HTSR_1544	hypothetical protein	0	2	0	0,000%	0,019%	0,000%
HTSR_1545	bacterio-opsin activator HTH domain-containing protein	0	2	2	0,000%	0,019%	0,048%
HTSR_1546	bacterio-opsin activator HTH domain-containing protein	0	0	0	0,000%	0,000%	0,000%
HTSR_1547	bacterio-opsin activator HTH domain-containing protein	0	0	0	0,000%	0,000%	0,000%
HTSR_1548	hypothetical protein	0	0	6	0,000%	0,000%	0,144%
HTSR_1549	binding-protein-dependent transport system innermembrane protein	0	0	0	0,000%	0,000%	0,000%
HTSR_1550	O-methyltransferase family 3	0	0	0	0,000%	0,000%	0,000%
HTSR_1551	ABC transporter substrate-binding protein	0	0	5	0,000%	0,000%	0,120%
HTSR_1552	ABC transporter ATP-binding protein	0	0	0	0,000%	0,000%	0,000%
HTSR_1553	binding-protein-dependent transport system innermembrane protein	0	0	0	0,000%	0,000%	0,000%
HTSR_1554	carbon monoxide dehydrogenase subunit G-like protein	0	0	0	0,000%	0,000%	0,000%
HTSR_1555	hypothetical protein	8	11	1	0,073%	0,104%	0,024%
HTSR_1556	4Fe-4S ferredoxin	0	0	0	0,000%	0,000%	0,000%
HTSR_1557	polysulfide reductase NrfD	1	1	0	0,009%	0,009%	0,000%
HTSR_1558	(2Fe-2S)-binding protein	1	0	0	0,009%	0,000%	0,000%
HTSR_1559	response regulator receiver protein	12	3	2	0,109%	0,028%	0,048%
HTSR_1560	ACT domain-containing protein	7	5	7	0,064%	0,047%	0,168%
HTSR_1561	Homoserine dehydrogenase	8	12	11	0,073%	0,113%	0,263%
HTSR_1562	elongation factor 1-alpha	46	61	52	0,417%	0,576%	1,245%
HTSR_1563	30S ribosomal protein S10	6	5	8	0,054%	0,047%	0,191%
HTSR_1565	hypothetical protein	0	0	4	0,000%	0,000%	0,096%
HTSR_1566	integrase	0	0	0	0,000%	0,000%	0,000%
HTSR_1567	ArsR family transcriptional regulator / Rhodanese domain protein	4	3	2	0,036%	0,028%	0,048%
HTSR_1568	hypothetical protein	4	5	2	0,036%	0,047%	0,048%
HTSR_1569	signal transduction protein	13	29	24	0,118%	0,274%	0,574%
HTSR_1570	hypothetical protein	0	0	0	0,000%	0,000%	0,000%
HTSR_1571	iron-sulfur binding protein, ferredoxin-like protein	4	11	3	0,036%	0,104%	0,072%
HTSR_1572	molecular chaperone TorD	10	6	0	0,091%	0,057%	0,000%
HTSR_1573	cytochrome b subunit of formate dehydrogenase	2	0	0	0,018%	0,000%	0,000%
HTSR_1574	hypothetical protein	0	0	0	0,000%	0,000%	0,000%
HTSR_1575	formate dehydrogenase	1	0	0	0,009%	0,000%	0,000%
HTSR_1576	anaerobic dehydrogenase subunit alpha	23	12	4	0,209%	0,113%	0,096%
HTSR_1577	hypothetical protein	3	13	9	0,027%	0,123%	0,215%
HTSR_1578	deoxyuridine 5'-triphosphate nucleotidohydrolase	0	0	0	0,000%	0,000%	0,000%
HTSR_1579	ribosomal-protein-alanine N-acetyltransferase	0	0	2	0,000%	0,000%	0,048%
HTSR_1580	hypothetical protein	2	4	2	0,018%	0,038%	0,048%
HTSR_1581	ADP-ribose pyrophosphatase	1	2	0	0,009%	0,019%	0,000%
HTSR_1582	hypothetical protein	0	0	0	0,000%	0,000%	0,000%
HTSR_1583	1,4-dihydroxy-2-naphthoate octaprenyltransferase / Menaquinone via futalosine polyprenyltransferase (MenA homolog), Nit	2	4	4	0,018%	0,038%	0,096%
HTSR_1584	ribonuclease R	20	30	14	0,181%	0,283%	0,335%
HTSR_1585	RNA-binding protein	2	4	1	0,018%	0,038%	0,024%
HTSR_1586	hypothetical protein	12	14	6	0,109%	0,132%	0,144%
HTSR_1587	hypothetical protein	3	2	2	0,027%	0,019%	0,048%
HTSR_1588	hypothetical protein	2	2	6	0,018%	0,019%	0,144%
HTSR_1589	hypothetical protein	0	0	0	0,000%	0,000%	0,000%
HTSR_1590	geranylgeranyl reductase	0	0	2	0,000%	0,000%	0,048%
HTSR_1591	metallophosphoesterase	0	2	1	0,000%	0,019%	0,024%
HTSR_1592	ATP dependent DNA ligase	4	18	11	0,036%	0,170%	0,263%
HTSR_1593	hypothetical protein	10	15	5	0,091%	0,142%	0,120%
HTSR_1594	excinuclease ABC subunit A	2	1	21	0,018%	0,009%	0,503%
HTSR_1595	aminomethyltransferase	22	16	16	0,200%	0,151%	0,383%
HTSR_1596	multidrug DMT transporter permease	0	1	1	0,000%	0,009%	0,024%
HTSR_1597	nitrogen-fixing protein NifU	0	0	0	0,000%	0,000%	0,000%
HTSR_1598	RNA-binding protein	4	0	6	0,036%	0,000%	0,144%
HTSR_1599	protease PrsW	0	2	0	0,000%	0,019%	0,000%
HTSR_1600	riboflavin synthase subunit alpha	0	1	3	0,000%	0,009%	0,072%
HTSR_1601	UvrD/REP helicase	9	0	27	0,082%	0,000%	0,646%
HTSR_1602	small CPxCG-related zinc finger protein	0	0	0	0,000%	0,000%	0,000%
HTSR_1603	ATPase	24	23	13	0,218%	0,217%	0,311%
HTSR_1604	ATPase	8	7	4	0,073%	0,066%	0,096%
HTSR_1605	ArsR family transcriptional regulator	1	0	0	0,009%	0,000%	0,000%
HTSR_1606	hypothetical protein	1	1	1	0,009%	0,009%	0,024%
HTSR_1607	ATPase RIL	11	20	15	0,100%	0,189%	0,359%
HTSR_1608	acetate/CoA ligase	46	48	65	0,417%	0,453%	1,556%
HTSR_1609	peptidase M54	1	0	8	0,009%	0,000%	0,191%
HTSR_1610	hypothetical protein	0	0	1	0,000%	0,000%	0,024%
HTSR_1611	HAD family hydrolase	2	5	7	0,018%	0,047%	0,168%

HTSR_1612	NADPH-dependent F420 reductase	3	4	0	0,027%	0,038%	0,000%
HTSR_1613	thioredoxin	4	6	7	0,036%	0,057%	0,168%
HTSR_1614	preprotein translocase subunit SecG	0	1	1	0,000%	0,009%	0,024%
HTSR_1615	hypothetical protein	7	8	7	0,064%	0,076%	0,168%
HTSR_1616	phosphoribosyl-ATP pyrophosphohydrolase	2	0	2	0,018%	0,000%	0,048%
HTSR_1617	GTP-binding protein	11	4	5	0,100%	0,038%	0,120%
HTSR_1618	hypothetical protein	2	3	4	0,018%	0,028%	0,096%
HTSR_1619	GTP-binding protein	1	2	8	0,009%	0,019%	0,191%
HTSR_1620	initiation factor 2B related protein	1	11	4	0,009%	0,104%	0,096%
HTSR_1621	coenzyme F420-0:L-glutamate ligase	0	0	4	0,000%	0,000%	0,096%
HTSR_1622	translation initiation factor Sui1	5	2	5	0,045%	0,019%	0,120%
HTSR_1623	ArsR family transcriptional regulator	0	0	0	0,000%	0,000%	0,000%
HTSR_1624	redox-active disulfide protein 2	0	0	0	0,000%	0,000%	0,000%
HTSR_1625	cysteine synthase A	3	0	6	0,027%	0,000%	0,144%
HTSR_1627	hypothetical protein	0	0	2	0,000%	0,000%	0,048%
HTSR_1628	hypothetical protein	0	0	0	0,000%	0,000%	0,000%
HTSR_1629	cob(I)alamin adenosyltransferase	1	0	3	0,009%	0,000%	0,072%
HTSR_1630	6-phosphogluconate dehydrogenase	9	17	17	0,082%	0,161%	0,407%
HTSR_1631	valyl-tRNA synthetase	28	50	32	0,254%	0,472%	0,766%
HTSR_1632	multidrug DMT transporter permease	0	0	0	0,000%	0,000%	0,000%
HTSR_1633	sulfate ABC transporter substrate-binding protein	6	17	5	0,054%	0,161%	0,120%
HTSR_1634	sulfate ABC transporter permease	0	0	0	0,000%	0,000%	0,000%
HTSR_1635	ABC transporter ATP-binding protein	0	0	0	0,000%	0,000%	0,000%
HTSR_1636	nicotinate phosphoribosyltransferase	14	25	11	0,127%	0,236%	0,263%
HTSR_1637	major facilitator superfamily MFS_1	2	0	1	0,018%	0,000%	0,024%
HTSR_1638	hypothetical protein	2	5	1	0,018%	0,047%	0,024%
HTSR_1639	hypothetical protein	0	0	1	0,000%	0,000%	0,024%
HTSR_1640	hypothetical protein	0	0	1	0,000%	0,000%	0,024%
HTSR_1641	dihydroorotase	4	16	13	0,036%	0,151%	0,311%
HTSR_1642	hypothetical protein	2	1	2	0,018%	0,009%	0,048%
HTSR_1643	universal stress protein UspA	3	6	6	0,027%	0,057%	0,144%
HTSR_1644	GCN5 family acetyltransferase	5	1	1	0,045%	0,009%	0,024%
HTSR_1645	recombinase RecJ	8	13	11	0,073%	0,123%	0,263%
HTSR_1646	hypothetical protein	3	5	1	0,027%	0,047%	0,024%
HTSR_1647	FAD-dependent pyridine nucleotide-disulfide oxidoreductase	2	15	1	0,018%	0,142%	0,024%
HTSR_1648	hypothetical protein	0	0	1	0,000%	0,000%	0,024%
HTSR_1649	30S ribosomal protein S6e	10	4	1	0,091%	0,038%	0,024%
HTSR_1650	peptide-methionine (S)-S-oxide reductase	4	3	2	0,036%	0,028%	0,048%
HTSR_1651	hypothetical protein	0	0	0	0,000%	0,000%	0,000%
HTSR_1652	MBL fold metallo-hydrolase	0	0	0	0,000%	0,000%	0,000%
HTSR_1654	filamentation induced by cAMP protein Fic	3	3	4	0,027%	0,028%	0,096%
HTSR_1655	PAS domain-containing sensor histidine kinase	7	11	6	0,064%	0,104%	0,144%
HTSR_1656	hypothetical protein	0	0	0	0,000%	0,000%	0,000%
HTSR_1657	anaerobic dehydrogenase subunit	13	10	8	0,118%	0,094%	0,191%
HTSR_1658	cytochrome b subunit of formate dehydrogenase	1	4	1	0,009%	0,038%	0,024%
HTSR_1659	hypothetical protein	0	3	1	0,000%	0,028%	0,024%
HTSR_1660	4Fe-4S ferredoxin iron-sulfur binding domain-containing protein	2	9	6	0,018%	0,085%	0,144%
HTSR_1661	molybdopterin oxidoreductase	16	28	39	0,145%	0,265%	0,933%
HTSR_1662	4Fe-4S ferredoxin	0	0	0	0,000%	0,000%	0,000%
HTSR_1663	bacterio-opsin activator HTH domain-containing protein	0	0	2	0,000%	0,000%	0,048%
HTSR_1664	molybdenum cofactor guanylyltransferase	2	0	1	0,018%	0,000%	0,024%
HTSR_1665	dinitrogenase iron-molybdenum cofactor biosynthesis protein	4	2	3	0,036%	0,019%	0,072%
HTSR_1666	metal-sulfur cluster biosynthetic enzyme	0	0	0	0,000%	0,000%	0,000%
HTSR_1667	hypothetical protein	1	3	0	0,009%	0,028%	0,000%
HTSR_1668	2-amino-3-ketobutyrate coenzyme A ligase	25	58	27	0,227%	0,548%	0,646%
HTSR_1669	toxin	0	1	1	0,000%	0,009%	0,024%
HTSR_1670	CopG family transcriptional regulator	1	0	0	0,009%	0,000%	0,000%
HTSR_1671	L-threonine 3-dehydrogenase	11	20	8	0,100%	0,189%	0,191%
HTSR_1672	aconitate hydratase	32	26	38	0,290%	0,246%	0,910%
HTSR_1673	aminoglycoside N3-acetyltransferase	0	2	5	0,000%	0,019%	0,120%
HTSR_1674	Fe3+-siderophore ABC transporter permease	0	1	0	0,000%	0,009%	0,000%
HTSR_1675	cobalamin/Fe3+-siderophore ABC transporter ATPase	0	0	0	0,000%	0,000%	0,000%
HTSR_1676	trimethylamine methyltransferase	5	0	8	0,045%	0,000%	0,191%
HTSR_1677	hypothetical protein	0	0	0	0,000%	0,000%	0,000%
HTSR_1678	cobalamin biosynthesis protein CobT	2	2	13	0,018%	0,019%	0,311%
HTSR_1679	AAA domain (dynein-related subfamily) / Nitric oxide reductase activation protein NorQ	6	0	20	0,054%	0,000%	0,479%
HTSR_1680	lipoate-protein ligase A	4	8	14	0,036%	0,076%	0,335%
HTSR_1681	hypothetical protein	0	0	0	0,000%	0,000%	0,000%
HTSR_1682	CopG family transcriptional regulator	1	1	1	0,009%	0,009%	0,024%
HTSR_1683	DsrE family protein	0	1	0	0,000%	0,009%	0,000%
HTSR_1684	3-oxoacyl-[acyl-carrier protein] reductase	6	0	15	0,054%	0,000%	0,359%
HTSR_1685	PAS sensor histidine kinase	1	20	17	0,009%	0,189%	0,407%
HTSR_1686	hypothetical protein	0	0	0	0,000%	0,000%	0,000%
HTSR_1687	O-acetylhomoserine (thiol)-lyase	19	19	23	0,172%	0,179%	0,551%
HTSR_1688	hypothetical protein	1	3	2	0,009%	0,028%	0,048%
HTSR_1689	cell surface glycoprotein related protein	0	0	0	0,000%	0,000%	0,000%
HTSR_1690	integrase/recombinase	0	0	0	0,000%	0,000%	0,000%
HTSR_1691	cell surface glycoprotein	0	0	0	0,000%	0,000%	0,000%
HTSR_1692	hypothetical protein	1	0	2	0,009%	0,000%	0,048%
HTSR_1693	hypothetical protein	11	8	6	0,100%	0,076%	0,144%
HTSR_1694	iron-dependent repressor	10	0	2	0,091%	0,000%	0,048%
HTSR_1695	hypothetical protein	7	1	0	0,064%	0,009%	0,000%
HTSR_1696	TrmB family transcriptional regulator	5	0	2	0,045%	0,000%	0,048%
HTSR_1697	polysulfide reductase NrfD	3	0	0	0,027%	0,000%	0,000%
HTSR_1698	4Fe-4S ferredoxin iron-sulfur binding domain-containing protein	0	0	0	0,000%	0,000%	0,000%
HTSR_1699	molybdopterin oxidoreductase	13	6	5	0,118%	0,057%	0,120%
HTSR_1700	4Fe-4S ferredoxin iron-sulfur binding domain protein	0	0	0	0,000%	0,000%	0,000%
HTSR_1701	IclR family transcriptional regulator	0	0	0	0,000%	0,000%	0,000%
HTSR_1702	hypothetical protein	1	0	2	0,009%	0,000%	0,048%
HTSR_1703	hypothetical protein	1	0	0	0,009%	0,000%	0,000%
HTSR_1704	tRNA-Thr(GGU) m(6)t(6)A37 methyltransferase TsaA	2	1	0	0,018%	0,009%	0,000%
HTSR_1705	ferredoxin	10	21	4	0,091%	0,198%	0,096%
HTSR_1706	uroporphyrinogen-III decarboxylase	12	28	5	0,109%	0,265%	0,120%
HTSR_1707	hypothetical protein	0	0	0	0,000%	0,000%	0,000%
HTSR_1708	Na+/proline symporter	0	2	0	0,000%	0,019%	0,000%
HTSR_1709	major facilitator superfamily MFS_1	0	0	0	0,000%	0,000%	0,000%
HTSR_1710	5-methyltetrahydrofolate--homocysteine methyltransferase	11	25	2	0,100%	0,236%	0,048%
HTSR_1711	B12 binding domain/Pterin binding enzyme	13	13	6	0,118%	0,123%	0,144%
HTSR_1712	uroporphyrinogen-III decarboxylase	5	21	6	0,045%	0,198%	0,144%
HTSR_1713	oxalate/formate antiporter family transporter	0	0	0	0,000%	0,000%	0,000%
HTSR_1714	hypothetical protein	0	1	1	0,000%	0,009%	0,024%
HTSR_1715	IclR family transcriptional regulator	8	11	19	0,073%	0,104%	0,455%
HTSR_1716	chromosome segregation protein SMC	24	34	16	0,218%	0,321%	0,383%
HTSR_1717	hypothetical protein	2	3	2	0,018%	0,028%	0,048%
HTSR_1718	hypothetical protein	0	0	0	0,000%	0,000%	0,000%

HTSR_1719	amidohydrolase	24	29	14	0,218%	0,274%	0,335%
HTSR_1720	ATP-grasp domain-containing protein	12	20	2	0,109%	0,189%	0,048%
HTSR_1721	phosphoribosyltransferase	6	8	5	0,054%	0,076%	0,120%
HTSR_1722	hypothetical protein	0	8	0	0,000%	0,076%	0,000%
HTSR_1723	radical SAM protein	0	0	0	0,000%	0,000%	0,000%
HTSR_1724	ATP-dependent DNA helicase PcrA	0	0	8	0,000%	0,000%	0,191%
HTSR_1725	hypothetical protein	0	0	1	0,000%	0,000%	0,024%
HTSR_1726	helicase	0	7	4	0,000%	0,066%	0,096%
HTSR_1727	hypothetical protein	0	0	0	0,000%	0,000%	0,000%
HTSR_1728	hypothetical protein	0	0	2	0,000%	0,000%	0,048%
HTSR_1729	hypothetical protein	0	0	0	0,000%	0,000%	0,000%
HTSR_1730	hypothetical protein	0	0	0	0,000%	0,000%	0,000%
HTSR_1731	hypothetical protein	1	0	4	0,009%	0,000%	0,096%
HTSR_1732	hypothetical protein	12	25	47	0,109%	0,236%	1,125%
HTSR_1733	hypothetical protein	0	0	7	0,000%	0,000%	0,168%
HTSR_1734	Predicted ATPase (AAA+ superfamily)	11	28	36	0,100%	0,265%	0,862%
HTSR_1736	formate dehydrogenase subunit alpha	39	66	34	0,354%	0,624%	0,814%
HTSR_1737	hypothetical protein	5	5	5	0,045%	0,047%	0,120%
HTSR_1738	trimethylamine:corrinoid methyltransferase	49	82	39	0,445%	0,775%	0,933%
HTSR_1739	formate--tetrahydrofolate ligase	64	107	61	0,581%	1,011%	1,460%
HTSR_1740	formate dehydrogenase subunit alpha	47	65	38	0,426%	0,614%	0,910%
HTSR_1741	hypothetical protein	0	1	3	0,000%	0,009%	0,072%
HTSR_1742	anaerobic dehydrogenase	5	5	1	0,045%	0,047%	0,024%
HTSR_1743	succinylglutamate desuccinylase/aspartoacylase	6	8	14	0,054%	0,076%	0,335%
HTSR_1744	ferredoxin	20	31	25	0,181%	0,293%	0,598%
HTSR_1745	tetrahydromethanopterin S-methyltransferase subunit H	23	37	26	0,209%	0,350%	0,622%
HTSR_1746	5,10-methylenetetrahydrofolate reductase	11	17	10	0,100%	0,161%	0,239%
HTSR_1747	methylenetetrahydrofolate dehydrogenase (NADP+)	45	46	27	0,408%	0,435%	0,646%
HTSR_1748	electron transfer flavoprotein subunit alpha	29	37	23	0,263%	0,350%	0,551%
HTSR_1749	electron transfer flavoprotein subunit beta	25	17	12	0,227%	0,161%	0,287%
HTSR_1750	glycine cleavage system protein T	95	192	103	0,862%	1,814%	2,465%
HTSR_1751	aldehyde ferredoxin oxidoreductase	4	0	11	0,036%	0,000%	0,263%
HTSR_1752	corrinoid methyltransferase	29	23	23	0,263%	0,217%	0,551%
HTSR_1753	choline/carnitine/betaine transporter	5	6	4	0,045%	0,057%	0,096%
HTSR_1754	dihydropteroate synthase	27	34	44	0,245%	0,321%	1,053%
HTSR_1755	competence/damage-inducible protein CinA	9	8	4	0,082%	0,076%	0,096%
HTSR_1756	hypothetical protein	17	9	2	0,154%	0,085%	0,048%
HTSR_1757	hypothetical protein	3	9	0	0,027%	0,085%	0,000%
HTSR_1758	cobalamin ABC transporter substrate-binding protein	16	10	2	0,145%	0,094%	0,048%
HTSR_1759	cobalamin biosynthesis protein CbiB	6	5	0	0,054%	0,047%	0,000%
HTSR_1760	cobalamin 5'-phosphate synthase	2	2	1	0,018%	0,019%	0,024%
HTSR_1761	adenosylcobinamide-phosphate guanylyltransferase	1	1	0	0,009%	0,009%	0,000%
HTSR_1762	threonine-phosphate decarboxylase	7	9	7	0,064%	0,085%	0,168%
HTSR_1763	adenosylcobinamide hydrolase	3	2	0	0,027%	0,019%	0,000%
HTSR_1764	adenosylcobyric acid synthase	4	11	2	0,036%	0,104%	0,048%
HTSR_1765	diphosphomevalonate decarboxylase	3	6	10	0,027%	0,057%	0,239%
HTSR_1766	NADH-binding protein	12	24	14	0,109%	0,227%	0,335%
HTSR_1767	hypothetical protein	0	0	0	0,000%	0,000%	0,000%
HTSR_1768	hypothetical protein	1	0	1	0,009%	0,000%	0,024%
HTSR_1769	multidrug ABC transporter ATP-binding protein	1	5	4	0,009%	0,047%	0,096%
HTSR_1770	creatinine amidohydrolase	6	6	6	0,054%	0,057%	0,144%
HTSR_1771	histidine kinase	0	0	0	0,000%	0,000%	0,000%
HTSR_1772	hypothetical protein	1	2	1	0,009%	0,019%	0,024%
HTSR_1773	NAD-dependent epimerase/dehydratase	2	13	14	0,018%	0,123%	0,335%
HTSR_1774	chlorite dismutase	8	8	6	0,073%	0,076%	0,144%
HTSR_1775	hypothetical protein	2	3	5	0,018%	0,028%	0,120%
HTSR_1776	cobalt transport ATP-binding protein	5	3	11	0,045%	0,028%	0,263%
HTSR_1777	cobalt transport ATP-binding protein	1	0	1	0,009%	0,000%	0,024%
HTSR_1778	cobalt transport protein	4	1	1	0,036%	0,009%	0,024%
HTSR_1780	DNA-3-methyladenine glycosylase II	0	0	3	0,000%	0,000%	0,072%
HTSR_1781	membrane protein	0	1	0	0,000%	0,009%	0,000%
HTSR_1782	replication factor A	10	5	13	0,091%	0,047%	0,311%
HTSR_1783	transcription factor CBF/NF-Y/histone domain-containing protein	8	9	7	0,073%	0,085%	0,168%
HTSR_1784	tRNA nucleotidyltransferase (CCA-adding enzyme)	15	25	24	0,136%	0,236%	0,574%
HTSR_1785	ZIP family metal transporter	0	0	0	0,000%	0,000%	0,000%
HTSR_1788	hypothetical protein	1	2	0	0,009%	0,019%	0,000%
HTSR_1789	bacterio-opsin activator HTH domain-containing protein	0	0	0	0,000%	0,000%	0,000%
HTSR_1790	hydroxylamine reductase	3	0	5	0,027%	0,000%	0,120%
HTSR_1791	hypothetical protein / Formiminotetrahydrofolate cyclodeaminase (EC 4.3.1.4)	0	0	0	0,000%	0,000%	0,000%
HTSR_1792	IMP dehydrogenase	9	9	7	0,082%	0,085%	0,168%
HTSR_1793	3,4-dihydroxy-2-butanone 4-phosphate synthase	4	3	7	0,036%	0,028%	0,168%
HTSR_1794	CTP-dependent riboflavin kinase	27	13	24	0,245%	0,123%	0,574%
HTSR_1795	tRNA-modifying enzyme	0	3	2	0,000%	0,028%	0,048%
HTSR_1796	arginyl-tRNA synthetase	19	25	21	0,172%	0,236%	0,503%
HTSR_1797	septum site-determining protein MinD	3	0	7	0,027%	0,000%	0,168%
HTSR_1798	phosphoesterase RecJ domain-containing protein	1	3	7	0,009%	0,028%	0,168%
HTSR_1799	peptide chain release factor 1	20	9	17	0,181%	0,085%	0,407%
HTSR_1800	multi-sensor signal transduction histidine kinase	8	38	24	0,073%	0,359%	0,574%
HTSR_1801	hypothetical protein	1	1	0	0,009%	0,009%	0,000%
HTSR_1802	A-type ATP synthase subunit D	10	13	2	0,091%	0,123%	0,048%
HTSR_1803	methionine synthase	16	15	7	0,145%	0,142%	0,168%
HTSR_1804	A-type ATP synthase subunit B	28	48	32	0,254%	0,453%	0,766%
HTSR_1805	A-type ATP synthase subunit A	23	31	21	0,209%	0,293%	0,503%
HTSR_1806	A-type ATP synthase subunit F	2	2	2	0,018%	0,019%	0,048%
HTSR_1807	A-type ATP synthase subunit C	19	31	27	0,172%	0,293%	0,646%
HTSR_1808	A-type ATP synthase subunit E	10	12	9	0,091%	0,113%	0,215%
HTSR_1809	A-type ATP synthase subunit K	0	0	0	0,000%	0,000%	0,000%
HTSR_1810	A-type ATP synthase subunit I	13	15	4	0,118%	0,142%	0,096%
HTSR_1811	hypothetical protein	12	9	4	0,109%	0,085%	0,096%
HTSR_1812	SAM-dependent methyltransferase	7	17	19	0,064%	0,161%	0,455%
HTSR_1813	hypothetical protein	8	8	11	0,073%	0,076%	0,263%
HTSR_1814	hypothetical protein	6	5	4	0,054%	0,047%	0,096%
HTSR_1815	electron transfer flavoprotein alpha/beta-subunit	3	10	6	0,027%	0,094%	0,144%
HTSR_1816	electron transfer flavoprotein alpha subunit	2	5	4	0,018%	0,047%	0,096%
HTSR_1817	geranylgeranyl diphosphate synthase, type I	2	0	2	0,018%	0,000%	0,048%
HTSR_1818	hypothetical protein	5	10	8	0,045%	0,094%	0,191%
HTSR_1819	radical SAM protein	1	0	1	0,009%	0,000%	0,024%
HTSR_1820	circadian clock protein, KaiC	3	7	9	0,027%	0,066%	0,215%
HTSR_1821	NAD+ kinase	2	8	4	0,018%	0,076%	0,096%
HTSR_1822	GTP cyclohydrolase I	27	19	34	0,245%	0,179%	0,814%
HTSR_1823	phage T7 F exclusion suppressor FxsA	1	1	0	0,009%	0,009%	0,000%
HTSR_1825	cytotoxic translational repressor of toxin-antitoxin stability system	1	0	0	0,009%	0,000%	0,000%
HTSR_1826	CopG family transcriptional regulator	1	0	1	0,009%	0,000%	0,024%
HTSR_1827	hypothetical protein	0	0	0	0,000%	0,000%	0,000%
HTSR_1828	poly(3-hydroxyalkanoate) polymerase subunit PhaC	9	4	3	0,082%	0,038%	0,072%

HTSR_1829	hypothetical protein	0	0	0	0,000%	0,000%	0,000%
HTSR_1830	2,3-bisphosphoglycerate-independent phosphoglycerate mutase	7	7	3	0,064%	0,066%	0,072%
HTSR_1831	amidohydrolase	3	8	11	0,027%	0,076%	0,263%
HTSR_1832	replication protein H	0	0	0	0,000%	0,000%	0,000%
HTSR_1833	cobyrinic acid a,c-diamide synthase	2	0	1	0,018%	0,000%	0,024%
HTSR_1834	precorrin-2 dehydrogenase / sirohydrochlorin ferrochelatase	0	0	0	0,000%	0,000%	0,000%
HTSR_1835	uroporphyrin-III C-methyltransferase	0	0	0	0,000%	0,000%	0,000%
HTSR_1836	cobalt transport protein CbiM	0	0	0	0,000%	0,000%	0,000%
HTSR_1837	cobalt transport protein CbiN	0	0	0	0,000%	0,000%	0,000%
HTSR_1838	cobalt ECF transporter T component CbiQ	0	0	0	0,000%	0,000%	0,000%
HTSR_1839	cobalt ABC transporter ATP-binding protein	0	0	0	0,000%	0,000%	0,000%
HTSR_1840	precorrin-6Y C5,15-methyltransferase (decarboxylating) subunit CbiT	1	1	1	0,009%	0,009%	0,024%
HTSR_1841	precorrin-2/cobalt-factor-2 C20-methyltransferase	0	0	0	0,000%	0,000%	0,000%
HTSR_1842	precorrin-4/cobalt-precorrin-4 C11-methyltransferase	4	0	0	0,036%	0,000%	0,000%
HTSR_1843	cobalamin biosynthesis protein CbiG	1	2	0	0,009%	0,019%	0,000%
HTSR_1844	cobalt-precorrin-2 C(20)-methyltransferase	0	0	0	0,000%	0,000%	0,000%
HTSR_1845	precorrin-3B C17-methyltransferase	4	0	1	0,036%	0,000%	0,024%
HTSR_1846	cobalamin (vitamin B12) biosynthesis CbiX protein	14	15	3	0,127%	0,142%	0,072%
HTSR_1847	Mg-chelatase subunit ChlI	19	13	0	0,172%	0,123%	0,000%
HTSR_1848	cobaltochelatase subunit CobN	13	10	0	0,118%	0,094%	0,000%
HTSR_1849	precorrin-8X/cobalt-precorrin-8 methylmutase	8	5	0	0,073%	0,047%	0,000%
HTSR_1850	cobalt-precorrin-6Y C(5)-methyltransferase	5	0	0	0,045%	0,000%	0,000%
HTSR_1851	ABC-type nitrate/sulfonate/bicarbonate transportsystem, periplasmic component	9	8	1	0,082%	0,076%	0,024%
HTSR_1852	ABC-type nitrate/sulfonate/bicarbonate transportsystem, permease component	2	0	0	0,018%	0,000%	0,000%
HTSR_1853	ABC-type nitrate/sulfonate/bicarbonate transportsystem, ATPase component	2	0	0	0,018%	0,000%	0,000%
HTSR_1854	cupin	1	2	0	0,009%	0,019%	0,000%
HTSR_1855	isocitrate dehydrogenase	25	21	19	0,227%	0,198%	0,455%
HTSR_1856	asparaginase	1	4	1	0,009%	0,038%	0,024%
HTSR_1857	methionyl aminopeptidase	6	12	11	0,054%	0,113%	0,263%
HTSR_1858	HIT family hydrolase, diadenosine tetraphosphatehydrolase	8	7	2	0,073%	0,066%	0,048%
HTSR_1859	hypothetical protein	1	1	0	0,009%	0,009%	0,000%
HTSR_1860	hypothetical protein	9	8	6	0,082%	0,076%	0,144%
HTSR_1861	MiaB-like tRNA modifying enzyme	0	0	2	0,000%	0,000%	0,048%
HTSR_1862	deoxyribose-phosphate aldolase	8	9	3	0,073%	0,085%	0,072%
HTSR_1863	hypothetical protein	1	3	2	0,009%	0,028%	0,048%
HTSR_1864	translation initiation factor IF-2B subunit delta	7	18	11	0,064%	0,170%	0,263%
HTSR_1865	hypothetical protein	0	0	1	0,000%	0,000%	0,024%
HTSR_1866	ribokinase	0	4	1	0,000%	0,038%	0,024%
HTSR_1867	uridine phosphorylase	13	4	6	0,118%	0,038%	0,144%
HTSR_1868	GCN5-related N-acetyltransferase	8	6	6	0,073%	0,057%	0,144%
HTSR_1869	glutamyl-tRNA(Gln) amidotransferase subunit D	12	25	8	0,109%	0,236%	0,191%
HTSR_1870	ArsR family transcriptional regulator	8	14	14	0,073%	0,132%	0,335%
HTSR_1871	hypothetical protein	0	0	0	0,000%	0,000%	0,000%
HTSR_1872	peptide-methionine (R)-S-oxide reductase	2	1	1	0,018%	0,009%	0,024%
HTSR_1873	transcriptional regulator	4	0	7	0,036%	0,000%	0,168%
HTSR_1874	tRNA(Ile2) 2-agmatinylcytidine synthetase	0	11	13	0,000%	0,104%	0,311%
HTSR_1875	hypothetical protein	0	0	0	0,000%	0,000%	0,000%
HTSR_1876	threonine synthase	1	5	10	0,009%	0,047%	0,239%
HTSR_1877	succinylglutamate desuccinylase/aspartoacylase	3	5	7	0,027%	0,047%	0,168%
HTSR_1878	potassium channel protein	17	23	17	0,154%	0,217%	0,407%
HTSR_1879	citrate synthase	10	15	13	0,091%	0,142%	0,311%
HTSR_1880	threonine dehydratase	21	12	23	0,191%	0,113%	0,551%
HTSR_1881	endoribonuclease L-PSP	6	9	2	0,054%	0,085%	0,048%
HTSR_1882	hypothetical protein	1	0	0	0,009%	0,000%	0,000%
HTSR_1884	thermosome subunit beta	32	26	30	0,290%	0,246%	0,718%
HTSR_1885	cytotoxic translational repressor of toxin-antitoxin stability system	0	0	0	0,000%	0,000%	0,000%
HTSR_1886	CopG family transcriptional regulator	0	0	0	0,000%	0,000%	0,000%
HTSR_1887	AsnC family transcriptional regulator	2	6	2	0,018%	0,057%	0,048%
HTSR_1888	glutamine synthetase	27	88	19	0,245%	0,831%	0,455%
HTSR_1889	hypothetical protein	3	3	1	0,027%	0,028%	0,024%
HTSR_1890	hypothetical protein	0	0	0	0,000%	0,000%	0,000%
HTSR_1891	ribonuclease BN	2	2	2	0,018%	0,019%	0,048%
HTSR_1892	tRNA (guanine26-N2/guanine27-N2)-dimethyltransferase	6	8	13	0,054%	0,076%	0,311%
HTSR_1893	zinc finger SWIM domain protein	0	0	0	0,000%	0,000%	0,000%
HTSR_1894	imidazole glycerol phosphate synthase subunit HisH	2	1	1	0,018%	0,009%	0,024%
HTSR_1895	uracil-DNA glycosylase, family 4	5	2	0	0,045%	0,019%	0,000%
HTSR_1896	hypothetical protein	3	2	2	0,027%	0,019%	0,048%
HTSR_1897	death domain-associated protein	0	0	0	0,000%	0,000%	0,000%
HTSR_1898	hydroxyacylglutathione hydrolase	0	0	1	0,000%	0,000%	0,024%
HTSR_1899	50S ribosomal protein L40e	3	2	4	0,027%	0,019%	0,096%
HTSR_1900	hypothetical protein	6	4	4	0,054%	0,038%	0,096%
HTSR_1901	hypothetical protein	0	2	2	0,000%	0,019%	0,048%
HTSR_1902	bile acid:sodium symporter	1	1	1	0,009%	0,009%	0,024%
HTSR_1903	seryl-tRNA synthetase	27	33	17	0,245%	0,312%	0,407%
HTSR_1904	histidine kinase	1	14	6	0,009%	0,132%	0,144%
HTSR_1905	hypothetical protein	8	10	10	0,073%	0,094%	0,239%
HTSR_1906	hypothetical protein	5	17	7	0,045%	0,161%	0,168%
HTSR_1907	membrane-bound mannosyltransferase	0	2	0	0,000%	0,019%	0,000%
HTSR_1908	ATPase AAA	20	27	21	0,181%	0,255%	0,503%
HTSR_1909	ATP-binding protein	0	0	2	0,000%	0,000%	0,048%
HTSR_1910	hypothetical protein	0	1	0	0,000%	0,009%	0,000%
HTSR_1911	hypothetical protein	0	1	4	0,000%	0,009%	0,096%
HTSR_1912	hypothetical protein	2	4	3	0,018%	0,038%	0,072%
HTSR_1913	translation initiation factor IF-2 subunit gamma	11	19	17	0,100%	0,179%	0,407%
HTSR_1914	RNA-binding protein	0	0	0	0,000%	0,000%	0,000%
HTSR_1915	DNA-directed RNA polymerase subunit E'	5	5	2	0,045%	0,047%	0,048%
HTSR_1916	DNA-directed RNA polymerase subunit E''	1	1	0	0,009%	0,009%	0,000%
HTSR_1917	hypothetical protein	0	0	0	0,000%	0,000%	0,000%
HTSR_1918	30S ribosomal protein S24e	12	2	7	0,109%	0,019%	0,168%
HTSR_1919	30S ribosomal protein S27ae	0	0	2	0,000%	0,000%	0,048%
HTSR_1920	bifunctional UGMP family protein/serine/threonine protein kinase	3	0	2	0,027%	0,000%	0,048%
HTSR_1921	hypothetical protein	5	10	5	0,045%	0,094%	0,120%
HTSR_1922	deoxyribonucleotide triphosphate pyrophosphatase	3	5	6	0,027%	0,047%	0,144%
HTSR_1923	hypothetical protein	5	2	4	0,045%	0,019%	0,096%
HTSR_1924	phosphate ABC transporter ATP-binding protein	0	0	1	0,000%	0,000%	0,024%
HTSR_1925	ABC-type tungstate transport system, periplasmiccomponent	0	0	1	0,000%	0,000%	0,024%
HTSR_1926	ABC-type tungstate transport system, permease component	1	0	0	0,009%	0,000%	0,000%
HTSR_1927	molybdenum-binding protein	5	14	12	0,045%	0,132%	0,287%
HTSR_1928	phosphate ABC transporter, substrate-binding protein	16	13	17	0,145%	0,123%	0,407%
HTSR_1929	phosphate ABC transporter, permease protein PstC	1	2	1	0,009%	0,019%	0,024%
HTSR_1930	phosphate ABC transporter, permease protein PstA	2	6	1	0,018%	0,057%	0,024%
HTSR_1931	phosphate ABC transporter, ATP-binding protein	9	18	8	0,082%	0,170%	0,191%
HTSR_1932	hypothetical protein	1	0	1	0,009%	0,000%	0,024%
HTSR_1933	glyoxalase/bleomycin resistance protein/dioxygenase	3	12	3	0,027%	0,113%	0,072%
HTSR_1934	phospholipase/carboxylesterase	1	1	1	0,009%	0,009%	0,024%

HTSR_1935	metal-dependent hydrolase	0	0	0	0,000%	0,000%	0,000%
HTSR_1936	potassium transporter TrkA	0	7	4	0,000%	0,066%	0,096%
HTSR_1937	potassium transporter TrkA	0	7	2	0,000%	0,066%	0,048%
HTSR_1938	potassium channel protein	1	4	3	0,009%	0,038%	0,072%
HTSR_1940	hypothetical protein	1	1	2	0,009%	0,009%	0,048%
HTSR_1941	NAD+ synthase	7	7	5	0,064%	0,066%	0,120%
HTSR_1943	hypothetical protein	1	1	8	0,009%	0,009%	0,191%
HTSR_1944	translation initiation factor Sui1	1	4	1	0,009%	0,038%	0,024%
HTSR_1945	bacterio-opsin activator	7	17	10	0,064%	0,161%	0,239%
HTSR_1946	hypothetical protein	0	1	0	0,000%	0,009%	0,000%
HTSR_1947	metalloprotease/metallo peptidase	0	1	1	0,000%	0,009%	0,024%
HTSR_1948	protein of unknown function DUF502	0	5	6	0,000%	0,047%	0,144%
HTSR_1949	lysyl-tRNA synthetase, class I	9	8	13	0,082%	0,076%	0,311%
HTSR_1950	uridylate kinase	1	4	2	0,009%	0,038%	0,048%
HTSR_1951	bifunctional molybdopterin-guanine dinucleotide biosynthesis protein MobB/MoaE	9	10	3	0,082%	0,094%	0,072%
HTSR_1952	hypothetical protein	0	0	0	0,000%	0,000%	0,000%
HTSR_1953	ADP-ribose pyrophosphatase	4	1	2	0,036%	0,009%	0,048%
HTSR_1954	queuine/archaeosine tRNA-ribosyltransferase	11	12	15	0,100%	0,113%	0,359%
HTSR_1955	archaeosine tRNA-guanine transglycosylase	10	19	22	0,091%	0,179%	0,527%
HTSR_1956	hypothetical protein	7	6	6	0,064%	0,057%	0,144%
HTSR_1957	hypothetical protein	6	4	4	0,054%	0,038%	0,096%
HTSR_1958	MoaD family protein	3	0	2	0,027%	0,000%	0,048%
HTSR_1959	hypothetical protein	1	0	0	0,009%	0,000%	0,000%
HTSR_1960	hypothetical protein	0	0	0	0,000%	0,000%	0,000%
HTSR_1961	3-methyl-2-oxobutanoate hydroxymethyltransferase	14	6	7	0,127%	0,057%	0,168%
HTSR_1962	aldehyde ferredoxin oxidoreductase	70	120	52	0,635%	1,134%	1,245%
HTSR_1963	elongator protein 3/MiaB/NifB	2	7	8	0,018%	0,066%	0,191%
HTSR_1964	hypothetical protein	0	0	0	0,000%	0,000%	0,000%
HTSR_1965	phosphoribosylformylglycinamidine synthase	7	7	6	0,064%	0,066%	0,144%
HTSR_1966	phosphoribosylformylglycinamidine synthase	6	6	6	0,054%	0,057%	0,144%
HTSR_1967	phosphoribosylaminoimidazole-succinocarboxamide synthase	9	23	9	0,082%	0,217%	0,215%
HTSR_1968	7,8-didemethyl-8-hydroxy-5-deazariboflavin synthase subunit CofH	0	0	2	0,000%	0,000%	0,048%
HTSR_1969	7,8-didemethyl-8-hydroxy-5-deazariboflavin synthase subunit CofG	0	0	0	0,000%	0,000%	0,000%
HTSR_1970	2-phospho-L-lactate guanylyltransferase	0	0	0	0,000%	0,000%	0,000%
HTSR_1971	hypothetical protein	15	9	12	0,136%	0,085%	0,287%
HTSR_1972	cell division GTPase FtsZ	15	19	30	0,136%	0,179%	0,718%
HTSR_1973	NAD-dependent epimerase/dehydratase	5	11	5	0,045%	0,104%	0,120%
HTSR_1974	dTMP kinase	2	7	5	0,018%	0,066%	0,120%
HTSR_1975	ArsR family transcriptional regulator	0	0	0	0,000%	0,000%	0,000%
HTSR_1976	AsnC family transcriptional regulator	2	2	1	0,018%	0,019%	0,024%
HTSR_1977	hypothetical protein	2	2	0	0,018%	0,019%	0,000%
HTSR_1978	universal stress protein UspA	7	10	4	0,064%	0,094%	0,096%
HTSR_1979	ATPase AAA	20	23	20	0,181%	0,217%	0,479%
HTSR_1980	NurA domain-containing protein	9	11	11	0,082%	0,104%	0,263%
HTSR_1981	hypothetical protein	0	0	0	0,000%	0,000%	0,000%
HTSR_1982	transposase, IS605 OrfB family	0	0	2	0,000%	0,000%	0,048%
HTSR_1983	transcriptional regulator	0	0	0	0,000%	0,000%	0,000%
HTSR_1984	holliday junction resolvase	1	1	1	0,009%	0,009%	0,024%
HTSR_1985	DNA polymerase sliding clamp	10	13	10	0,091%	0,123%	0,239%
HTSR_1986	23S rRNA methyltransferase J	6	5	5	0,054%	0,047%	0,120%
HTSR_1987	integral membrane protein	1	2	1	0,009%	0,019%	0,024%
HTSR_1988	CopG family transcriptional regulator	0	1	0	0,000%	0,009%	0,000%
HTSR_1989	hypothetical protein	0	0	1	0,000%	0,000%	0,024%
HTSR_1990	preprotein translocase subunit TatC	2	6	2	0,018%	0,057%	0,048%
HTSR_1991	preprotein translocase subunit TatC	0	0	1	0,000%	0,000%	0,024%
HTSR_1992	DNA mismatch repair protein MutS	9	29	26	0,082%	0,274%	0,622%
HTSR_1993	Na+-dependent transporter, SNF family	0	0	0	0,000%	0,000%	0,000%
HTSR_1994	hypothetical protein	0	0	2	0,000%	0,000%	0,048%
HTSR_1995	cell division control protein 6	2	15	9	0,018%	0,142%	0,215%
HTSR_1996	peptidyl-prolyl cis-trans isomerase A (cyclophilin A)	13	13	5	0,118%	0,123%	0,120%
HTSR_1998	hypothetical protein	0	0	0	0,000%	0,000%	0,000%
HTSR_1999	phosphoribosylamine--glycine ligase	0	12	4	0,000%	0,113%	0,096%
HTSR_2000	hypothetical protein	0	1	1	0,000%	0,009%	0,024%
HTSR_2001	DNA primase large subunit	9	10	10	0,082%	0,094%	0,239%
HTSR_2002	phosphatidylserine decarboxylase	2	4	5	0,018%	0,038%	0,120%
HTSR_2003	ribose 5-phosphate isomerase A	3	5	3	0,027%	0,047%	0,072%
HTSR_2004	DNA-binding protein	4	6	4	0,036%	0,057%	0,096%
HTSR_2005	phosphomannomutase / phosphoglucomutase	19	34	27	0,172%	0,321%	0,646%
HTSR_2006	mechanosensitive ion channel protein MscS	1	2	2	0,009%	0,019%	0,048%
HTSR_2007	acetyltransferase	0	0	0	0,000%	0,000%	0,000%
HTSR_2008	hypothetical protein / Ubiquitin-like small archaeal modifier protein SAMP2	0	0	0	0,000%	0,000%	0,000%
HTSR_2009	hypothetical protein	0	3	0	0,000%	0,028%	0,000%
HTSR_2010	replication factor C small subunit	10	11	10	0,091%	0,104%	0,239%
HTSR_2011	alanyl-tRNA synthetase	31	34	24	0,281%	0,321%	0,574%
HTSR_2012	hypothetical protein	0	0	0	0,000%	0,000%	0,000%
HTSR_2013	PHP domain-containing protein	0	0	1	0,000%	0,000%	0,024%
HTSR_2014	hypothetical protein	0	0	0	0,000%	0,000%	0,000%
HTSR_2015	hypothetical protein	0	0	0	0,000%	0,000%	0,000%
HTSR_2016	methyltransferase	0	1	2	0,000%	0,009%	0,048%
HTSR_2017	NMD3 family protein	2	4	12	0,018%	0,038%	0,287%
HTSR_2018	heat shock protein HtpX	5	2	3	0,045%	0,019%	0,072%
HTSR_2019	hypothetical protein	1	2	1	0,009%	0,019%	0,024%
HTSR_2020	DNA repair and recombination protein RadA	16	11	26	0,145%	0,104%	0,622%
HTSR_2021	hypothetical protein	3	8	3	0,027%	0,076%	0,072%
HTSR_2022	SirA family protein	1	1	0	0,009%	0,009%	0,000%
HTSR_2023	NADPH-dependent 2,4-dienoyl-CoA reductase	14	18	11	0,127%	0,170%	0,263%
HTSR_2024	hypothetical protein	0	1	1	0,000%	0,009%	0,024%
HTSR_2025	iron-sulfur cluster scaffold-like protein	2	0	2	0,018%	0,000%	0,048%
HTSR_2026	cysteine desulfurase	22	21	20	0,200%	0,198%	0,479%
HTSR_2027	hypothetical protein	4	5	7	0,036%	0,047%	0,168%
HTSR_2028	hypothetical protein	1	1	1	0,009%	0,009%	0,024%
HTSR_2029	hypothetical protein	0	0	0	0,000%	0,000%	0,000%
HTSR_2030	tetratricopeptide repeat protein	10	0	8	0,091%	0,000%	0,191%
HTSR_2031	2'-5' RNA ligase	1	3	4	0,009%	0,028%	0,096%
HTSR_2032	50S ribosomal protein L39e	0	0	2	0,000%	0,000%	0,048%
HTSR_2033	50S ribosomal protein L31e	4	7	8	0,036%	0,066%	0,191%
HTSR_2034	translation initiation factor IF-6	4	4	5	0,036%	0,038%	0,120%
HTSR_2035	prefoldin subunit alpha	7	4	0	0,064%	0,038%	0,000%
HTSR_2036	signal recognition particle-docking protein FtsY	5	3	0	0,045%	0,028%	0,000%
HTSR_2037	hypothetical protein	2	3	0	0,018%	0,028%	0,000%
HTSR_2038	hypothetical protein	0	0	0	0,000%	0,000%	0,000%
HTSR_2039	signal recognition particle protein Srp54	24	15	14	0,218%	0,142%	0,335%
HTSR_2040	MgtE integral membrane region	1	1	1	0,009%	0,009%	0,024%
HTSR_2041	divalent cation transporter	0	0	0	0,000%	0,000%	0,000%
HTSR_2042	dephospho-CoA kinase-like protein	25	23	14	0,227%	0,217%	0,335%

HTSR_2044	hypothetical protein	0	0	0	0,000%	0,000%	0,000%
HTSR_2045	hypothetical protein	0	0	0	0,000%	0,000%	0,000%
HTSR_2046	amphi-Trp domain-containing protein	5	5	4	0,045%	0,047%	0,096%
HTSR_2047	small CPxCG-related zinc finger protein	0	0	0	0,000%	0,000%	0,000%
HTSR_2048	OsmC family protein	1	2	2	0,009%	0,019%	0,048%
HTSR_2049	threonine aldolase	15	20	8	0,136%	0,189%	0,191%
HTSR_2050	phosphoesterase	0	0	8	0,000%	0,000%	0,191%
HTSR_2051	DEAD/DEAH box helicase	9	19	12	0,082%	0,179%	0,287%
HTSR_2052	phosphoesterase	4	0	2	0,036%	0,000%	0,048%
HTSR_2053	hypothetical protein	0	0	0	0,000%	0,000%	0,000%
HTSR_2054	argininosuccinate synthase	10	19	12	0,091%	0,179%	0,287%
HTSR_2055	argininosuccinate lyase	24	25	13	0,218%	0,236%	0,311%
HTSR_2056	lysine biosynthesis protein LysW	0	1	0	0,000%	0,009%	0,000%
HTSR_2057	lysine biosynthesis enzyme LysX	10	10	19	0,091%	0,094%	0,455%
HTSR_2058	threonine synthase	13	20	13	0,118%	0,189%	0,311%
HTSR_2059	homoserine O-acetyltransferase	0	1	9	0,000%	0,009%	0,215%
HTSR_2060	two-component sensor histidine kinase	0	0	4	0,000%	0,000%	0,096%
HTSR_2061	DNA ligase (NAD+)	13	26	17	0,118%	0,246%	0,407%
HTSR_2062	hypothetical protein	0	0	5	0,000%	0,000%	0,120%
HTSR_2063	excinuclease ABC subunit C	0	0	3	0,000%	0,000%	0,072%
HTSR_2064	antibiotic biosynthesis monooxygenase	0	0	0	0,000%	0,000%	0,000%
HTSR_2065	excinuclease ABC subunit B	1	2	5	0,009%	0,019%	0,120%
HTSR_2066	permease	0	0	1	0,000%	0,000%	0,024%
HTSR_2067	ABC transporter substrate-binding protein	1	4	1	0,009%	0,038%	0,024%
HTSR_2068	ABC transporter permease	0	0	0	0,000%	0,000%	0,000%
HTSR_2069	ABC transporter ATP-binding protein	0	0	2	0,000%	0,000%	0,048%
HTSR_2070	SAM-dependent methyltransferase	5	4	3	0,045%	0,038%	0,072%
HTSR_2071	ATPase involved in flagella biogenesis	6	2	3	0,054%	0,019%	0,072%
HTSR_2072	Zn-ribbon containing protein (DUF2072)	0	0	1	0,000%	0,000%	0,024%
HTSR_2073	hypothetical protein	2	0	2	0,018%	0,000%	0,048%
HTSR_2074	small GTP-binding protein	13	0	19	0,118%	0,000%	0,455%
	**TOTAL NO. PEPTIDES**	**11021**	**10585**	**4178**	**100,000%**	**100,000%**	**100,000%**
